# The Management of Irrigation and Potassium Fertilization to Mitigate the Effect of Light Frosts on the Phenolic and Volatile Compounds in Virgin Olive Oils

**DOI:** 10.3390/antiox13050559

**Published:** 2024-05-01

**Authors:** Suony Antonelli, Sebastián Pozas, Jorge Saavedra-Torrico, Mauricio Donders, Chris Bustamante, Betsabet Sepúlveda, Francisco Tapia, Diego L. García-González, Nalda Romero

**Affiliations:** 1 Departamento de Ciencia de los Alimentos y Tecnología Química, Facultad de Ciencias Químicas y Farmacéuticas, Universidad de Chile, Santiago 8380000, Chile; s_antonelli@ug.uchile.cl (S.A.); sebastian.pozas@ug.uchile.cl (S.P.); 2 Escuela de Alimentos, DataChem Analytics, Pontificia Universidad Católica de Valparaíso, Valparaíso 2340025, Chile; jorge.saavedra@pucv.cl; 3 Panel de Cata Aceite de Oliva Virgen UTEM, Facultad de Ciencias Naturales, Matemática y del Medio Ambiente, Universidad Tecnológica Metropolitana, Santiago 7800002, Chile; mdonders@utem.cl (M.D.); pacao.pes@utem.cl (C.B.); 4 Centro Para el Desarrollo de la Química—CEPEDEQ, Facultad de Ciencias Químicas y Farmacéuticas, Universidad de Chile, Santiago 8380000, Chile; bsepulveda@ciq.uchile.cl; 5 Instituto de Investigaciones Agropecuarias (INIA Intihuasi), La Serena 1700000, Chile; fatapiac@inia.cl; 6 Instituto de la Grasa (CSIC), Edificio 46, Ctra. de Utrera, km. 1, 41013 Sevilla, Spain; dlgarcia@ig.csic.es

**Keywords:** phenols, volatile compounds, antioxidant capacity, olive oil, frost, fertilization, irrigation, sensory attributes

## Abstract

The frequency of early frosts has increased in recent years, which are injurious to olive growing, causing losses in the yield and quality of virgin olive oil. In this research, it was studied how the management of agronomic factors mitigates frost damage in Arbequina olives, minimizing the loss of phenols and volatiles in virgin olive oil, at different fruit ripening stages. A Box–Behnken design and multivariate analysis were performed, with three levels of irrigation, potassium fertilization, and foliar copper application (15 treatments). Virgin olive oil was extracted from fresh and frozen olives. Light frost caused a significant decrease in the total phenols and secoiridoid compounds in and the antioxidant capacity of the frost-affected oils, which were perceived as more pungent and had the slight defect of “frostbitten olives”. According to the Box–Behnken design, an 86% reference evapotranspiration (ET_0_) or higher with 100 potassium oxide units (UK_2_O) and a 100% ET_0_ or higher with 250 UK_2_O would be required to minimize the effect of light frost on phenols and volatiles. Partial Least Squares Regression–Discriminant Analysis (PLS-DA) differentiated the virgin olive oils according to their ripening stage and fresh and frost conditions. Moreover, PLS-DA positively correlated a 75–100% ET_0_ and 0 Uk_2_O with the dialdehydic form of the decarboxymethyl ligstroside aglycone (*p*-HPEA-EDA), the dialdehydic form of the decarboxymethyl oleuropein aglycone (3,4-DHPEA-EDA), the dialdehydic form of the ligstroside aglycone (*p*-HPEA-EDA-DLA), and with fruity, pungent, and bitter attributes. Precision agronomic management based on the needs of the crop itself would avoid unnecessary stress on olive trees and oil damage.

## 1. Introduction

The planet’s climate has changed throughout the last century. Global warming has caused highly unstable, intense, and uncertain weather patterns for activities like agriculture [1]. The average temperature has increased by almost one degree, although this has also caused drops in temperatures in very specific areas, accelerating polar airflows toward mid-latitudes [2]. In general, fruit crops located in the Andean cordillera or in the interior areas are more exposed and sensitive to climate change due to their greater susceptibility to losses due to frost and a high deficit of precipitation [3]. Frost events are among the climate threats that most concern agricultural producers because they can generate important economic losses, affecting cultivated plants and causing yield and quality losses in the products harvested [4,5]. In this regard, Chile has the interest of covering a wide variety of climates along its territory [6], with a wide temperate zone with long summers (northern zone), while at higher latitudes, on the contrary, summers are shorter, with frosts from late summer onwards and intensifying in autumn, coinciding with the middle of the harvest. The phenomenon of La Niña also favors greater nocturnal cooling, increasing the temperature drop and the number of frosts, making low areas prone to its damaging effects due to the greater density of cold air that tends to move and fill these spaces [4]. There have been considerable variations in the total number of frosts between 1961 and 2020. For example, 470 events were registered in Chile in 2020, experiencing an increased incidence of frost events in recent years, especially in the central zone of the territory during the months of May to August [4,5].

Sometimes, early frosts take place before winter, which are detrimental to various crops because they can abruptly interrupt harvest processes, affecting mainly fruit trees and perennial crops such as olives in Chile. A meteorological frost is defined as the air temperature at 1.50 m from the ground dropping to a value below or equal to the freezing point of water at 0 °C, regardless of its duration or intensity [4]. Below 0 °C, the water content in the olive tissues freezes, with the formation of extracellular and/or intracellular ice crystals. This causes tissue damage by destroying the cell membranes of the olive fruits; breaking the cellular balance between the antioxidant enzymes of the fruit and the production of ROS, generating oxidative stress and causing peroxidation of the lipids of the cell membranes; and causing high oxidation of olive oil components such as phenolic compounds, therefore affecting its antioxidant properties [7,8]. The processes involved in fruit ripening are intensified in fruits that have been damaged due to freezing, making the fruits lose water and ripen earlier, becoming brown, soft, and wrinkly [7].

Phenolic compounds are the primary antioxidants inhibiting oxidation processes in virgin olive oil (VOO), which act as chain-breakers by donating radical hydrogen to alkylperoxyl radicals. A positive correlation has been reported between the total phenols, antioxidant activity, and shelf life of extra virgin olive oil. The antioxidant activity of VOO largely depends on the relative concentrations of hydroxytyrosol and oleuropein-derived compounds, in particular the dialdehydic form of the decarboxymethyl oleuropein aglycone (3,4-DHPEA-EDA), also called oleacein, and the oleuropein aglycone (3,4-DHPEA-EA), identified as the compounds with the highest antioxidant efficacy in VOO [9]. Fuentes et al. [10] presented a model of antioxidant capacity measured using an oxygen radical absorbance capacity assay with fluorescein as the target molecule (H-ORACFL), dependent on hydroxytyrosol and an oleuropein derivative. Ramos-Escudero et al. [11] reported that the antioxidant capacity measured using different assays, including the H-ORACFL assay, is highly influenced by the phenolic content, especially the dialdehydic form of elenolic acid linked to tyrosol and hydroxytyrosol.

The Arbequina cultivar is widely distributed in the world, while quality and compositional differences associated with geographical location have been observed [6]. The oils of the Arbequina cultivar are typically characterized as fruity, sweet, slightly green, moderately bitter, and pungent [12]. The quality of VOO is affected by its composition of phenolic and volatile compounds [9,13], responsible for the flavor and aroma of the olive oil. Phenolic compounds influence the perception of a bitter and astringent taste, with the main compounds responsible for the pungent sensation being the dialdehydic form of the decarboxymethyl ligstroside aglycone (*p*-HPEA-EDA), also called oleocanthal, and, to a lesser extent, 3,4-DHPEA-EDA [14,15,16]. Meanwhile, when the lipoxygenase pathway enzymes are the most active, the formation of six-carbon volatile compounds, including hexanal, (E)-2-hexenal, 1-hexanol, and (E)-2-hexenol, is favored, thus generating a balanced flavor with positive green and fruity aroma attributes [13]. 

Frost phenomena influence the composition of these compounds and therefore the sensory quality of the oils, affecting in addition its antioxidant properties. Thus, the physiological and chemical changes associated with frozen olives lead to oils that are less pungent and bitter and with a defect defined as “frostbitten olives” by the International Olive Council (IOC). In addition, Romero et al. [17] described two sensory profiles associated with the “frostbitten olives” defect; the first profile was described as having “soapy” and “strawberry-like” sensory notes, whereas the other was described as having “wood” and “humidity” notes. The different profiles are explained by the different patterns in temperature drops, with the first sensory profile explained by several freeze and thaw cycles, compared to a gradual drop in temperature for the second profile.

The phenolic profile and the activity levels of the enzymes involved in the lipoxygenase pathway also depend on agronomic factors and technological variables such as the cultivar, the state of ripeness of the harvested fruits, climatic conditions, agricultural management of the crops, and harvest processes and oil extraction [13,15]. In recent work by our research group [18], the effect of a light frost, controlled by the application of freezing to the olives before the oil extraction, on the phenolic and volatile compounds was evidenced, considering also the ripening stage as an important factor. However, agricultural management (e.g., irrigation, fertilization) also affects the extent of frost damage, and as a consequence, the effect of frost on virgin olive oil’s quality. Thus, crop management techniques that affect irrigation can reduce the damage generated by frosts [15]. Sustainable irrigation becomes crucial to reduce the water use and costs in agricultural systems, facing the present scenarios of global warming and desertification [19]. Water limitation could help to improve the quality of olive oil if it is applied at critical times, producing virgin olive oil with higher levels of phenolic compounds, which provide a pungent sensation and antioxidant properties [20]. 

Hydric stress stimulates the synthesis of phenolic compounds in olive fruits, especially those derived from secoiridoids [19,21], improving the oil’s stability [21]. On the contrary, phenolic compounds such as lignans, vanillic acid, and vanillin increase under more irrigated instead [21]. In addition, olive trees subjected to water stress conditions have a negative influence on volatile compounds related to the lipoxygenase pathway, such as (E)-2-hexenal, hexanal, and 1-hexanol [20]. 

Fertilization is another criterion that may affect the processes that damage olive fruits. Potassium (K) is an essential macronutrient in olives; it allows for optimal plant growth, an increased fruit size, a high oil content, protein synthesis, and the accumulation of carbohydrates and fats in the fruits [22]. In grapevines, high potassium concentrations enhance the concentration of metabolites such as phenolic acids, polyamines, and soluble sugars, which are related to cold tolerance. It has also been found that high potassium levels increase plant defenses, helping them to cope with cold stress by serving as an osmolyte, thereby decreasing the sap freezing point and avoiding cell dehydration [22]. 

In this context, and considering the losses in virgin olive oil production caused by frequent frost events, more studies are required for efficient management of olive groves under these circumstances. In particular, it is necessary to study the changes in antioxidants due to frost in olives, taking into account the cross effect of different agronomic factors. The aim is not only to better understand the effect of frost on the antioxidant compounds in and the quality of virgin olive oil but also to explore the potential possibility of mitigating this effect when low temperatures occur using better agronomical control adapted to possible frost conditions. This study is necessary considering that many farmers are concerned about unexpected temperature changes before harvest. Thus, in the proposed research work, for a comprehensive understanding of the frost phenomena, a response surface Box–Behnken design (BBD) was applied, subjecting Arbequina olive crops to different levels of irrigation, potassium fertilization, and foliar oxychloride copper in conjunction. The aim of this work was to study how irrigation and potassium fertilization prevent or mitigate frost damage in Arbequina olive crops at different stages of maturation, minimizing the loss of virgin olive oil’s quality, especially its antioxidant capacity and flavor-related compounds (phenols and volatiles).

## 2. Materials and Methods

### 2.1. Reagents

All the HPLC-grade (methanol, acetonitrile, hexane) and analytical-grade (orthophosphoric acid, ethanol, ethyl acetate, sodium hydroxide, sodium thiosulfate) reagents were acquired from Merck (Darmstadt, Germany). The standards used to identify and quantify the phenolic compounds (*o*-coumaric acid, *p*-hydroxyphenylacetic acid, hydroxytyrosol, tyrosol, vanillic acid, syringic acid, vanillin, hydroxytyrosol acetate, *p*-coumaric acid, ferulic acid, tyrosol acetate, pinoresinol, luteolin, apigenin, methyl-luteolin) were obtained from Sigma-Aldrich (St. Louis, MO, USA). The standards used to identify and quantify the volatile compounds (4-methyl-2-pentanol, ethanol, ethyl propanoate, pentanal, 4-methylpentan-2-one, ethyl-2-methylbutyrate, butyl acetate, hexanal, 2-methylbutan-1-ol, 3-methylbutan-1-ol, (E)-2-hexenal, 3-octanone, octanal, (E)-2-heptenal, 2-heptanol, 1-hexanol, nonanal, (E)-2-nonenal, (E)-2-hexenol, acetic acid, propanoic acid, 1-octanol, butanoic acid, heptanoic acid) were purchased from Merck. Trolox, fluorescein, and 2,20-Azobis (2-amidinopropane) dihydrochloride (AAPH) were obtained from Sigma-Aldrich (St. Louis, MO, USA).

### 2.2. Orchard Characteristics and Agricultural Aspects

The present study was carried out during the months of August 2020 and July 2021, in an area of one hectare, in a 6-hectare olive grove of Arbequina-variety olive trees, which were 22 years old, planted at a high density (1300 trees per hectare, at a spacing of 5 × 1.75 m), and located in the Huasco Experimental Center, managed by the Agricultural Research Institute (INIA), in the Atacama Region, 446 m above sea level (latitude −28°34′43.70″ S and longitude −70°47′56.76″ W). The olive grove, located in the Copiapó agroclimatic zone, benefits from a coastal Mediterranean climate. The soil belongs to the Aridisol family; is located in a high terrace position; has little evolution, with a petrocalcic horizon between 0.3 and 0.8 m deep; has a loam to clay loam texture; and is moderate to alkaline (pH 7.5–8.2). Its electrical conductivity varies between 1.7 and 7.0 dS/m. Its organic matter decreases from 1% at surface to 0.1% at depth. The soil presents a nutrient availability of N, P, and K of 39, 11, and 191 mg/kg, with a cation exchange of 12.9 meq/100 g (sum of bases). The average maximum temperatures are 32 °C in summer, and the minimum temperatures are no less than 8 °C in winter. The other climatic conditions have been described by Pino et al. [18]. The olive trees were drip-irrigated daily, using the Penman–Monteith equation to obtain the hydric demand as the reference evapotranspiration (ET_0_), which was used to determine the daily irrigation according to the experimental design applied. A total irrigation gross demand of 7.477 m^3^/ha was calculated during the study period (12 months). 

### 2.3. Sample Selection and Processing

In this research, a Box–Behnken design was applied. A BBD is a type of so-called second-order design, i.e., it has a quadratic term in the polynomial model. For three factors, BBD considers 12 runs + 3 central points (total: 15 runs per block). A comparison between BBD and other response surface designs (central composite and three-level full factorial designs) has shown that BBD is much more efficient than three-level full factorial designs [23]. The olive trees were treated in accordance with the BBD, considering three agronomic factors with three levels each, as follows: Irrigation: 75% ET_0_ (5.202 m^3^/ha), 100% ET_0_ (6.950 m^3^/ha), and 125% ET_0_ (8.718 m^3^/ha); Fertilization was applied once per season to the soil in the root zone at a depth of 5 to 10 cm as potassium sulphate and expressed as potassium oxide units (UK_2_O): 100, 175, and 250 UK_2_O (kg/ha); Foliar application of copper oxychloride: 100, 200, and 300 g/100 L of water. Both were applied in September at the beginning of spring, when there was no fruit on the trees yet. 

A total of 15 combinations of experiments/runs of experimental conditions, as shown in Table 1, were assigned to random rows of trees with a similar vigor, age, production, and agronomic management, considering four trees for the experimental conditions in duplicate. Each treatment row contained 3 randomly assigned treatments. The treatment rows were separated by two rows of olive trees of similar characteristics not subjected to treatments or evaluations.

Three harvesting times (May, June, and July) were selected, with ripening indexes between 2 and 3, 3 and 4, and 4 and 5, respectively [24]. At each harvest, approximately 10 kg of olives with an average weight of 2.18 g and 1.56 × 1.38 cm in diameter were collected from each tree, with only healthy fruits without injury selected. The olives were mixed and divided into two portions. One portion of fresh olives was subjected to oil extraction, whereas the second portion was stored at −3 ± 1 °C for 12 h in a Z300 cold chamber freezer (Fensa, Santiago, Chile), then thawed at room temperature for 2 to 4 h, and later subjected to oil extraction. In this experimental design, considering the three ripeness levels and the two processing conditions, a total of 180 samples of VOO were processed, and they facilitated studying the different factors.

### 2.4. Virgin Olive Oil Extraction

The olives were processed using the Olimaker Professional MC3 1.0 model extraction equipment (Granada, Spain). Both types of fruits, fresh and frozen olives, were ground and then slowly mixed for 30 min at 26 ± 2 °C, and the resulting paste was centrifuged at 1027× *g* for 5 min to separate the oil. All the samples were filtered through hydrophilic cotton and stored in amber glass bottles in the dark at −23 °C until analysis (within 1 month). The samples were analyzed in triplicate according to the following analyses.

### 2.5. Quality Physicochemical Parameters

A physical–chemical characterization of the oils was carried out on every sample in accordance with the standard methods (1993) [25] of the American Oil Chemists’ Society (AOCS), using Ca 5a-40 for free fatty acids, Cd 8–53 for the peroxide value, and Ch 5–91 for the specific extinctions of the oils (K232, K270). Their color was measured using a Lovibond tintometer PFXi-195 instrument (Tintometer Inc., Sarasota, FL, USA) according to Pino et al. [18]. The quality parameters and their phenolic and volatile compounds were analyzed in the Laboratory of Food Chemistry of the Department of Food Science, the Faculty of Chemistry and Pharmaceutical Sciences, the University of Chile.

### 2.6. Determination of the Phenolic Compounds

Phenolic compounds were extracted using solid-phase extraction, as described by Caipo et al. [26], and analyzed using a high-performance liquid chromatography (HPLC) Waters system, coupled to a reverse-phase Spherisorb ODS RP-18 column (4.6 mm i.d. × 250 mm; 5 µm particle size) and equipped with a diode array UV detector (model 2998), a binary pump (model 1525), and an autosampler (model 2707). Simple phenols, phenolic acids, flavones (luteolin, methyl luteolin, and apigenin), and pinoresinol were identified and quantified using standards with their respective calibration curves (in triplicate). Five different concentrations, ranging from 0.01 to 500 µg/mL, were used, obtaining determination coefficients (R^2^) ranging between 0.995 and 1.000. The secoiridoid compounds were identified and quantified according to Pino et al. [18]. For the quantification of elenolic acid at 235 nm and simple phenols, phenolic acids, and secoiridoids at 280 nm, *p*-hydroxyphenylacetic acid was used as the internal standard. In addition, *o*-coumaric acid was used as an internal standard for the quantification of flavonoids at 335 nm. The concentration values were expressed in mg/kg.

### 2.7. Hydrophilic ORAC Assay

Phenolic compounds were extracted from the VOO samples in methanol/water (80/20) using liquid/liquid extraction and quantified as described by Fuentes et al. [10]. The antioxidant capacity of the phenolic extracts was measured according to ORAC, as described by Fuentes et al. [10]. AAPH (2,20-azobis (2-amidino-propane) dihydrochloride), fluorescein, Trolox calibration solution (12.5–100 µM), and the phenolic extracts were dissolved in 0.075 M phosphate buffer (pH 7.4). The ORAC assays were performed using an FLx800-TBID fluorescence reader (BioTek, Winooski, VT, USA), and the antioxidant capacity was expressed as µmol of Trolox equivalent (TE)/g of oil.

### 2.8. Determination of the Volatile Compounds

Determination of the volatile compounds was performed according to Caipo et al.’s [26] methodology, using as an internal standard 4-methyl-2-pentanol. First, the volatiles in the samples were equilibrated using a Headspace HT280T autosampler (HTA s.r.I, Brescia, Italy), controlled using the HT-COMSOFT software 2.0 version (HTA s.r.I). Then, the volatiles were adsorbed onto a solid-phase microextraction (SPME) fiber (2 cm length and 50/30 µm film thickness) composed of a flexible and stable divinylbenzene/carboxen/polydimethylsiloxane (DVB/CAR/PDMS) stationary phase, acquired from Supelco (Bellefonte, PA, USA). Afterwards, the volatile compounds were desorbed in the injection port using the purge valve (splitless mode) of a Shimadzu GC-2010 Plus gas chromatograph (Shimadzu, Kyoto, Japan), coupled to a TR-WAX capillary column (60 m × 0.25 mm i.d., 0.25 µm coating) (Teknokroma, Barcelona, Spain) and a flame ionization detector (FID) (Shimadzu, Kyoto, Japan). The data processing was carried out on a PC using Workstation software ver. 2 (Shimadzu, Kyoto, Japan). The volatiles were identified and quantified using calibration curves (in triplicate). Five different concentrations of standard were used in a range from 6 to 1000 µg/mL, obtaining determination coefficients (R^2^) ranging between 0.976 and 0.999. All the concentrations are expressed in mg/kg.

### 2.9. Sensory Assessment of the Olive Oils

Sensory analysis of the VOO was carried out by 9 tasters trained in the methodology proposed by COI-T20-Doc.-15 [27], belonging to the Olive Oil Tasting Panel of the Sensory Evaluation Program of the Metropolitan Technological University, recognized by the IOC. The tasting panel proceeded to evaluate the sensory attributes (fruitiness, pungency, and bitterness) and defects (“frostbitten olives”, rancid, fusty/muddy sediment, musty–humid–earthy, and winey–vinegary). Each sample (15 mL) was tasted in a normalized, blue-colored glass container, maintaining the oil at 28 ± 2 °C [27]. The results are expressed as the mean intensity of the sensory perceptions of the tasters.

### 2.10. Statistical Analysis

The 15 different treatments in every harvest according to the BBD were averaged and analyzed using ANOVA and the multiple comparison test with a Least Square Distance Fisher analysis (LSD-Fisher) method, using Statgraphics V18 (The Plains, Virginia, USA, 2023), to detect significant differences between the studied groups. Then, a multivariate analysis based on the NIPALS algorithm (nonlinear iterative partial least squares) [28] was carried out. Both the principal component analysis (PCA) and Partial Least Squares Regression–Discriminant Analysis (PLS-DA) analysis were carried out using SIMCA-P + 14 (MKS Umetrics AB, Malmö, Sweden, 2016). The datasets were centered and scaled to the unit variance. To avoid the overfitting of the models, the analyses were validated using a full cross-validation routine, minimizing the Prediction Residual Sum of the Squares function (PRESS) [29].

For optimization using the BBD, the outliers in the design were detected and analyzed using residue analysis and the Grubbs test [30]. The anomalous data were modeled using Bootstrap, using the means and standard deviation of the data (*n* = 100 or 500) based on the central theorem of the de Moivre–Laplace limit and the theorem of large numbers in probability [31]. Subsequently, the data were trimmed using the Trim or Winsor (15%) methods, which prescinded the tails of the distributions, replacing them with values close to the mean.

All the raw data obtained from the different analyses are presented in Table S1 in the Supplementary Materials.

## 3. Results and Discussion

### 3.1. Effect of the Ripening Stage of the Olives and Frost Phenomena on the Quality Parameters, Phenolic and Volatile Compounds, and Sensory Perception of Arbequina VOOs

#### 3.1.1. Quality and Color Parameters

Table 2 shows the means for the quality and color parameters determined using Fisher’s LSD multiple comparison intervals at 95% confidence for the VOOs from fresh olives (Fresh) and the VOOs from frozen olives (Frost) in the 3 months of harvest.

This table shows a slight increase in the free fatty acids and K232 for the fresh oils in the month of July (*p* < 0.05), without significant differences between May and June (*p* > 0.05). However, the peroxide value did not vary with the ripening stage in these oils, while for the K270 index, a slight decrease was observed with ripening. When comparing the oils from the fresh and frozen olives, it was observed that the free fatty acids were significantly lower (*p* < 0.05) in the oils from the fresh olives compared to the oils from the frozen olives, explained by the interaction of lipases with triglycerides due to the rupture of the cellular tissue on freezing the fruit [32]. In the case of the peroxide value, a lower value (*p* < 0.05) was obtained for the oils from the frozen olives, attributed to a decrease in LOX enzymatic activity [33]. As for the K232 and K270 values, lower values of these coefficients were observed in May for the frost oils, showing a lower oxidative alteration of the oils in this month.

Regarding the color parameters, the L* values increased as the ripening stage progressed in the case of the oils from the fresh olives, showing significant differences between May and July (*p* < 0.05), while their b* values decreased significantly (*p* < 0.05), attributed to slight decreases in chlorophyll and carotenoid content [7]. The oils from the frozen olives showed a higher brightness and greener hues, especially in May and June, compared to the oils from the fresh olives, presenting also more yellow hues in May and July when frost conditions were applied to the olives.

#### 3.1.2. Phenolic Compounds

**Figure 1:**
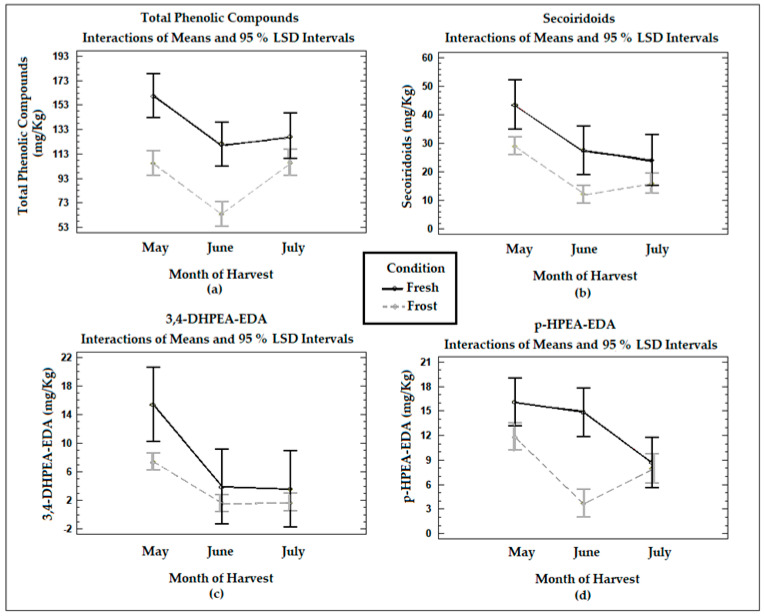
Graphs show the means and Fisher’s LSD multiple comparison intervals at 95% confidence of (**a**) total phenols, (**b**) secoiridoids, (**c**) 3,4−DHPEA−EDA (oleacein), and (**d**) *p*−HPEA−EDA (oleocanthal) in Arbequina VOOs from fresh (Fresh) and frozen olives (Frost).

Figure 1 shows the means of the treatments determined using Fisher’s LSD multiple comparison intervals at 95% confidence in terms of the total phenol and secoiridoid compounds (obtained from the sum of the content of the dialdehyde form of the ligstroside and oleuropein aglycones, the aldehydic and hydroxylic forms of the oleuropein aglycone, 3,4-DHPEA-EDA, and *p*-HPEA-EDA), 3,4-DHPEA-EDA, and *p*-HPEA-EDA in the VOO from the fresh and frozen olives during the three months of harvest. The total phenols in the VOO from the fresh olives decreased significantly (*p* < 0.05) between May and June (mean values of 160 mg/kg and 121 mg/kg, respectively), with no significant changes between June and July (*p* > 0.05) (mean values of 121 mg/kg and 128 mg/kg, respectively). The changes in the concentration of total phenols were influenced by the time at which the olives were harvested. The maximum content of phenols was found when the fruit was at veraison, and as maturity progressed, the value began to decrease. The concentration decrease observed in the total phenols between May and June can be explained by the decrease in phenylalanine ammonia-lyase (PAL) activity in olive fruits during ripening [34].

The VOO samples from the frozen olives suffered a significant (*p* < 0.05) decrease in their total phenolic content when compared to the VOOs from the fresh olives, although the difference was not significant in July (*p* > 0.05). The decrease in the total phenols in the VOOs from the frozen olives can be attributed to the formation of intra- or extracellular ice crystals in the fruits because of the freezing conditions. The damage produced in the fruit tissues will trigger a series of oxidative reactions, significantly affecting phenolic compounds, as already discussed in a publication with previous results [18].

The secoiridoid content in the VOOs from the fresh olives experienced a decrease with ripening, being statistically significant (*p* < 0.05) between May and July. The secoiridoid concentrations in the VOOs also decreased significantly (*p* < 0.05) with olive freezing, especially in May and June. In the specific cases of the main secoiridoid compounds, 3,4-DHPEA-EDA decreased with ripening in the VOOs from the fresh olives, being significant (*p* < 0.05) between May and June. This is consistent with the report of Morelló et al. [15], who described a lower amount of secoiridoid derivatives with maturity, mainly affecting 3,4-DHPEA-EDA. The concentrations of *p*-HPEA-EDA in the VOO from the fresh olives also registered a decrease with ripeness, being significant (*p* < 0.05) between May and July. A decrease in the concentrations of 3,4-DHPEA-EDA and *p*-HPEA-EDA was also observed in the oils from the frozen olives when compared to the fresh olive samples, being significant (*p* < 0.05) in May in the case of 3,4-DHPEA-EDA and in June in the case of *p*-HPEA-EDA. A reduction in secoiridoid compounds will generate a reduction in pungent and bitter tastes [3]. These results agree with previous studies, where a decrease in both types of secoiridoids with ripening was observed [18].

#### 3.1.3. Antioxidant Capacity

The efficiency of an antioxidant is generally influenced by its molecular structure, concentration, temperature, and type of oxidation substrate, among other things. The chemical structure of an antioxidant determines its reactivity towards free radicals and other ROS and therefore its antioxidant activity [35]. The presence of different substituents in phenol backbone structures modulates their antioxidant properties, in particular their hydrogen-donating capacities [36].

**Figure 2:**
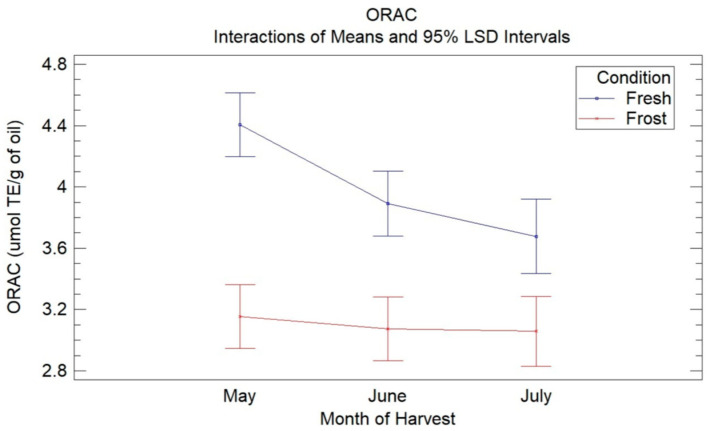
Graphic shows the means of treatments determined using Fisher’s LSD multiple comparison intervals at 95% confidence for ORAC assays in VOO from fresh and frozen olives during the three months of harvest.

Figure 2 shows the means of the treatments determined using Fisher’s LSD multiple comparison intervals at 95% confidence for the ORAC assays in the VOOs from the fresh and frozen olives during the three months of harvest. In this, a decrease in the antioxidant capacity of the VOO from the fresh olives with harvest time was observed (4.4–3.6 µmol TE/g oil), being significant between May and the months of June and July, with no significant differences observed between these latter two months. The decrease in the antioxidant capacity can be explained mainly by a decrease in phenolic compounds, specifically secoiridoid compounds, with the ripening of the olives, as already mentioned. These results are similar to those obtained in previous studies [18]. Wang et al. [37] reported a decrease in antioxidant capacity in three VOO cultivars with fruit ripening.

When comparing the antioxidant capacity of the oils from the fresh olives with the oils from the frozen olives, a significant decrease in the antioxidant capacity was observed in the latter for the three harvest months, without observing significant differences between the harvest months in the oils affected by frost. The loss of antioxidant capacity could be mainly due to the decrease in secoiridoid compounds, mainly 3,4-DHPEA-EDA, due to the oxidative processes generated by the frost.

Compounds that possess *o*-dihydroxyl functionalities in their structure have been characterized by a high antioxidant activity because of the formation of intramolecular hydrogen bonds during reactions with free radicals [36]. Thus, *o*-diphenols, hydroxytyrosol, and secoiridoids containing this compound in their molecular structure (namely 3,4-DHPEA-EDA, 3,4-DHPEA-EA) have been identified as the natural antioxidants in VOO with the highest antioxidant power [38]. On the other hand, it is expected that tyrosol and ligstroside aglycones, which are monosubstituted phenols, have a lower contribution to the antioxidant capacity of oils [36]. Both types of oils, from the fresh olives and from the frozen olives, presented low contents of hydroxytyrosol (<1 mg/kg of oil), which could suggest the low influence of this compound on the antioxidant capacity of the oils.

#### 3.1.4. Volatile Compounds

**Figure 3:**
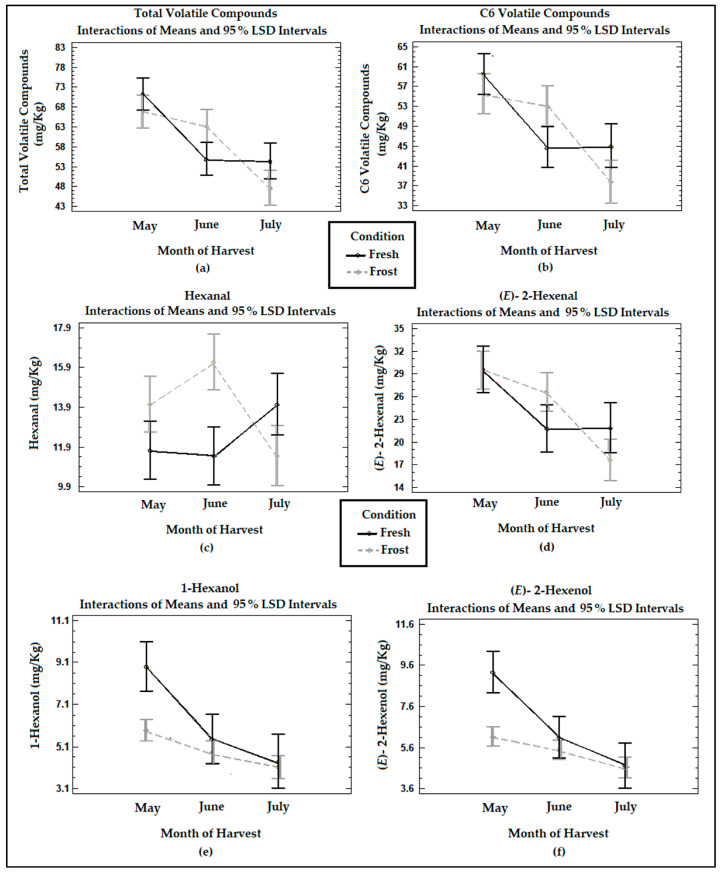
Graphs show the means and Fisher’s LSD multiple comparison intervals at 95% confidence of (**a**) total volatile compounds, (**b**) C6 volatile compounds, (**c**) hexanal, (**d**) (E)-2-hexenal, (**e**) 1-hexanol, and (**f**) (E)-2-hexenol in Arbequina VOOs from fresh (Fresh) and frozen olives (Frost).

Figure 3 shows the means of the treatments determined using Fisher’s LSD method at the 95% confidence level in terms of the total volatile compounds, six-carbon (C6) volatile compounds originating from the LOX pathway, and hexanal, (E)-2-hexenal, 1-hexanol, and (E)-2-hexenol determined in the VOOs from the fresh and frozen olives during the three harvest months. The main loss of volatile compounds was registered between May and June, maintaining their concentrations between June and July. The total volatiles and C6 compounds formed by the lipoxygenase cascade in the VOO from the fresh olives showed a significant decrease (*p* < 0.05) in their concentrations between May and June. In the study carried out by Pino et al. [18], no significant differences (*p* > 0.05) were observed in the concentration of total volatile compounds with ripening.

In the VOO from the fresh olives, the compounds (E)-2-hexenal, (E)-2-hexenol, and 1-hexanol underwent a significant (*p* < 0.05) decrease between May and June. Meanwhile, hexanal experienced a non-significant increase (*p* > 0.05) in its concentrations in the last month of harvesting. Hachicha Hbaieb et al. [39] reported a decrease in the C6 aldehydes of the LOX pathway in Arbequina with ripening, while C6 alcohols were not significantly affected (*p* > 0.05). This decrease would be due to a reduction in the enzymatic activity of the LOX enzyme since HPL maintains a high activity during the ripening period [39].

The frost produced a non-significant decrease (*p* > 0.05) in the total volatiles and C6 volatiles in the oils during the months of May and July. This is consistent with García-Vico et al. [40], who described a decrease in the enzymatic activities of LOX, HPL, ADH, and AAT (located in the seeds) in olives from the Arbequina cultivar frozen for 3 days at −18 °C, reducing the C6 and C5 volatile compounds.

The frost generated a sharp decrease in the C6 alcohols, 1-hexanol, and (E)-2-hexenol at the two earlier ripening stages, May and June, observing a minimum effect between June and July. The reduction in C6 alcohols derived from linoleic and linolenic acid in the frozen olives coincides with the decrease in ADH activities when olive fruits are frozen [40].

Contrary to what was expected, the oils from the frozen fruits underwent an increase in hexanal and (E)-2 hexenal in the month of June, of 34% and 18%, respectively. This could be associated with a slight increase in the moisture in the olives, which leads to an increment in intracellular ice crystals, damaging the fruit tissues and triggering autoxidation reactions, as was already mentioned. The increase in these compounds also was reflected in the increase, non-significant (*p* > 0.05), in total volatile and C6 volatile compounds in the oils from the frozen olives in this month. Against these last findings, Pino et al. [18] reported a lower development of volatile compounds from the LOX cascade in oils from frozen olives, such as hexanal, (E)-2-hexenal, 1-hexanol, and (E)-2-hexenol, due to the freezing of the tissue and the inactivation of the enzymes involved in the lipoxygenase cascade.

#### 3.1.5. Sensory Analysis Results

Since the data provided in the sensory test had a normal distribution, the medians were averaged. The defects fusty/muddy sediment, musty–humid–earthy, winey, and rancid were not found or were only described in a small number of samples, so they were not taken into account in the analysis.

**Figure 4:**
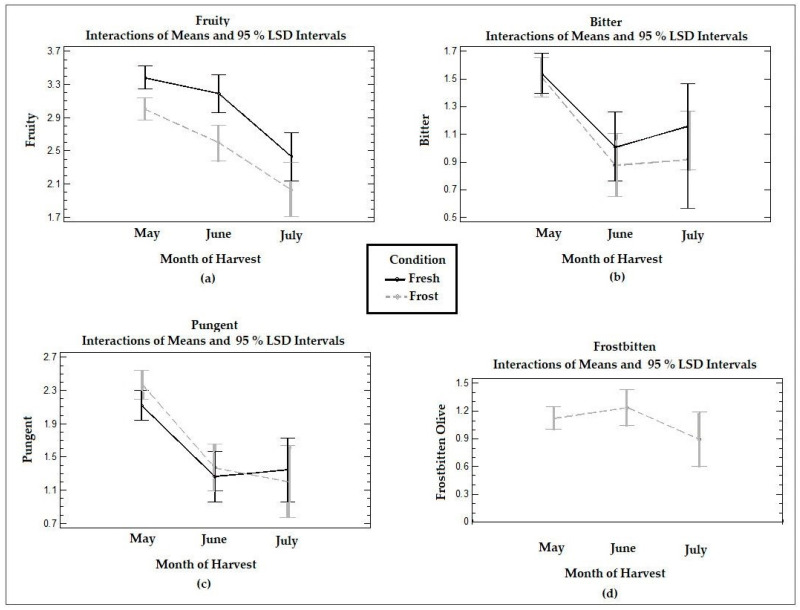
Graphs of means and LSD multiple comparison intervals at 95% confidence for the positive attributes (**a**) fruity, (**b**) bitter, and (**c**) pungency and the (**d**) “frostbitten olives” sensory defect in Arbequina VOOs from fresh (Fresh) and frozen olives (Frost). The panelists did not report “frostbitten olives” defects in the oils collected from fresh olives.

Figure 4 shows the time trends in the medians reported in the panel test for the positive attributes and defects in the VOOs from the fresh and frozen olives at the three ripening stages. The VOO samples analyzed by the tasting panel were described as moderately fruity oils, mildly bitter and pungent at all three harvest stages, with a lighter perception in July. In general, oils from the Arbequina cultivar are characterized as fruity, sweet, slightly green, and moderately bitter and pungent [12], which coincides with what was described by the trained tasting panel.

The fruity attribute was significantly (*p* < 0.05) reduced between June and July in the VOOs of the fresh olives, while both the bitter and pungency attributes decreased significantly (*p* < 0.05) between May and June. VOO tasting panels may describe a stronger sensory intensity when fruit is harvested at the incipient stages of ripeness, for example, according to a ripening index of 3–4 [41]. In this study, the highest intensity of fruity, pungent, and bitter tastes was obtained with a ripening index of 2–3, corresponding to the month of May.

When comparing the VOO from the fresh olives with the VOO from the frozen olives, a significant reduction (*p* < 0.05) in the fruity attribute was observed in May and June for the latter, whereas no significant (*p* > 0.05) differences were observed for the pungent and bitter attributes. The medians for the pungent attribute in May and June were higher in the VOOs from the frozen olives compared to those of the fresh olives, although the difference was not significant (*p* > 0.05).

The general descriptions of the sensory characteristics of the oils obtained from the frozen olives also were consistent with the characteristics of oils obtained from trees that are affected by frost phenomena. The tissue destruction produced by frost promotes degradation reactions, which causes the oils to be characterized as softer, less pungent, bitter, and fruity with the presence of the “frostbitten olives” sensory defect [18]. A study carried out using olives stored at −20 °C described alterations in the quality of the oils, including a reduction in the concentration of phenols and generation of the “frostbitten olives” sensory defect [42]. The perception of defects associated with frozen olives have also been described as wet grass or stewed fruit flavors, appearing even in olives obtained immediately after frost [43]. Other authors have reported perceptions of “soapy” and “strawberry-like” and “woody” and “wet” flavors [17]. In the VOOs from the frozen olives studied in this work, the presence of the “frostbitten olives” defect was described, although with a low score, which did not vary significantly (*p* > 0.05) with ripening. In the fresh olive VOOs, the tasting panel did not perceive the “frostbitten olives” defect.

### 3.2. Effect of the Agronomic Factors on the Content of Phenolic and Volatile Compounds in and the Sensory Perception of the VOOs from Fresh and Frozen Olives

The BBD, which included the 15 experiments using fresh and frozen olive oils across 3 months of harvest, was analyzed to study the effects and interactions associated with the agronomic factors applied. The foliar use of copper oxychloride was not considered in the analysis since it was used as a dummy factor for the use of the design.

#### 3.2.1. The Effect of Irrigation and Potassium Fertilization on the Total Phenolic Compounds and Antioxidant Capacity

Table 3 shows the optimal irrigation and potassium fertilization values for every condition and month of harvest, which allowed us to determinate the highest content of phenols in and the antioxidant capacity of the oils from the fresh and frozen olives. In this study, optimization of the agronomic factors displayed the expected values for total phenols between 111 and 251 mg/kg of oil. In addition, it was observed that at the early stages of ripening (May), higher concentrations of total phenols in VOOs are possible to obtain when the olive trees are subjected to less irrigation, with a 75% ET_0_ for the fresh olive VOOs and an 86% ET_0_ for the frozen olive VOOs. However, at the later stages of ripening, total phenols are mainly favored in the presence of more intensive irrigation treatments, with a 125% ET_0_ for the fresh olive VOOs and between a 110% and 125% ET_0_ for the frozen olive VOOs. It has been reported that water stress stimulates the synthesis of phenolic compounds in olive fruits [34], especially secoiridoids [21]. Romero et al. [44] reported higher amounts of total phenols in extra virgin olive oils from Arbequina, Koroneiki, and Arbosana crops which had received less irrigation. García et al. [45] found higher concentrations of total phenols, secoiridoids, and orthodiphenols when 30% RDI (Regulated Deficit Irrigation) was applied during three harvest seasons. Tovar et al. [46] related more irrigation with a reduction in the PAL enzyme activity and consecutively a decline in the synthesis of phenolic compounds. Sastre et al. [47], on the other hand, comparing a 40% RDI restraint with 100% irrigated olives, did not observe a significant variation in the phenol content of the oils [47].

Maybe more severe water restriction is required to visualize a significant increase in phenolic compounds [46], but excessive water stress could also negatively affect the growth and productivity of the crop [48]. The reduction in the content of phenolic compounds was associated with the presence of severe stress in the oil synthesis period, with the increase in the phenolic compounds favored when water restriction was applied only during the pit-hardening period. The former behavior can be explained by the increased oxidative enzymatic activity in the olives during the oil synthesis period. Therefore, it is assumed that increased irrigation does not always lead to a reduction in the content of phenolic compounds, and the existence of a water stress threshold is necessary to induce an increment in the secoiridoid content [49].

For potassium fertilization, in this study, it was observed that 250 UK_2_O was required to maximize the total phenols in the VOOs from the fresh olives obtained in earlier ripening states, while at the later stages of ripening, the lowest treatment of potassium fertilization generated higher phenol concentrations. However, for the VOOs obtained from the frozen olives, the application of 100 UK_2_O of potassium as fertilizer allowed for the highest concentrations of total phenols, regardless of the ripening state.

In the literature, the relationship between phenolic compounds and irrigation has been mainly described without involving potassium fertilization. Tekaya et al. [50] found that potassium fertilization had little influence on the concentration of phenolic compounds, while authors such as Pascual et al. [51], on the other hand, found a significant reduction in the stability of the oil and the phenol content with potassium fertilization. Tognetti et al. [52] associated overdoses of potassium fertilizer in the soil with a reduction in the quality of VOO, generating a decrease in the total content of phenols in the oil.

It is believed that potassium promotes the enzymatic activity of PPO, stimulating the gene expression of this enzyme, which ends up negatively affecting the content of *o*-diphenols in the oil [51]. In maize, potassium has been associated with a lower transcription of PAL in the roots and with an increase in the activity of POD and PPO as a defense mechanism [53]. The availability of water is important to determine the response of plants to mineral nutrition [54]. A nitrogen/potassium interaction has also been evidenced, where potassium can increase the nitrogen content in the leaves [50]. Nitrogen has been associated with a reduction in the total phenol content by decreasing PAL activity and increasing PPO activity [54]. Romero et al. [44] also evidenced the negative effect of high concentrations of nitrogen and potassium, caused by increased PPO activity, which catalyzes the oxidation of *o*-diphenols into quinones.

Because of the complexity of solving of the high variability of the data for the BBD, only the antioxidant capability (ORAC) of the May data was considered. In the subsequent months, the mean average error (MAE) was very high, and the determination coefficient (R2 adj) had low values.

The antioxidant capacity for the March harvest followed the same trend as that for the total phenolic compounds for the oils from the fresh olives, with optimal irrigation and potassium fertilization conditions of a 75% ET_0_ and 250 UK_2_O, respectively. Meanwhile, for the oils from the frozen olives, the optimal ORAC values were obtained at a 125% ET_0_ and 100 UK_2_O. Under these conditions, optimum antioxidant capacity values of 5.58 and 4.57 µmol of TE/g of oil, respectively, would be expected.

#### 3.2.2. The Effect of Irrigation and Potassium Fertilization on Total Volatile Compounds

Since no oils were affected by rancidity and most of the volatile compounds identified were those coming from the LOX pathway (C6), the optimal values for irrigation and potassium fertilization were determined for every condition and month of harvest, allowing the highest content of volatiles in the VOOs from the fresh and frozen olives (Table 4). In general, lower potassium fertilization levels were necessary to obtain the highest levels of total volatile compounds in the VOOs from the fresh olives. The highest level of potassium was required to obtain the highest concentration of total volatile compounds in the VOOs from the frozen olives, mainly in June and July. But at more unripe stages (May), the potassium requirement for the highest concentrations of total volatiles was lower (117 UK_2_O). Dabbaghi et al. [55] established that the simultaneous use of three foliar biofertilizers, nitrogen, phosphorus, and potassium, in Chemlali VOOs seemed to significantly improve the levels of most volatile compounds, especially the ones produced via the LOX cascade, such as hexanal. However, none of the studies related potassium fertilization directly to the formation of volatile compounds.

The maximum level of irrigation (a 125% ET_0_) was necessary to obtain the highest concentrations of total volatile compounds, especially in May and June, for both conditions. Meanwhile, medium levels of irrigation (a 100% ET_0_) were required to obtain the highest concentrations of total volatile compounds in July.

The behavior of the volatile compounds is strongly associated with the cultivar, the climatic and agronomic conditions [56], and the LOX isoform present [57] and is also affected by the water status of the tree throughout the growing season [56]. It has been described that the volatile compounds related to the LOX pathway are positively affected by higher levels of irrigation, favoring the concentrations of (E)-2-hexenol, (E)-2-hexenal, hexanal, and 1-hexanol, responsible for green/fruity sensory perceptions [34]. Romero et al. [44] reported higher concentrations of total volatile compounds in EVOOs from Arbequina, Koroneiki, and Arbosana crops which had received higher levels of irrigation compared to less irrigated varietal orchards in more arid zones. García et al. [49] described the obtainment of a high quantity of total volatiles and C6 volatiles in Arbequinas, especially of the compound (E)-2-hexenal, in the presence of complete irrigation. García-Garví et al. [58] also obtained higher concentrations of total volatiles, hexanal, and (E)-2-hexenal in fully irrigated olives.

#### 3.2.3. Effect of Irrigation and Potassium Fertilization on the Sensory Analysis Results

Table 5 shows the optimal values for irrigation and potassium fertilization for every condition in month of May, which allowed us to obtain the highest median values for the positive attributes (fruity, pungent, and bitter) in the VOOs from the fresh and frozen olive oils. The optimization of the agronomic variables for the sensory attributes was carried out only for the month of May since the highest values for the positive attributes of the oils presented in this month.

In order to obtain the highest median scores for the fruity and bitter attributes in the VOOs from the fresh olives, irrigation of a 75% ET_0_ and fertilization of 250 UK_2_O are preferred. On the other hand, in the VOOs from the frozen olives, an optimal irrigation of 75% ET_0_ was determined to obtain the maximum median values for the fruity, pungent, and bitter attributes. In the case of fertilization, the lowest level (100 UK_2_O) was associated with the highest median values for the fruity and bitter attributes, while 204 UK_2_O was necessary for the maximum pungent attribute value. With the optimized variables, the fruity, bitter, and pungency scores would be expected to be well above the values determined by the testing panel for the 15 experiments, especially for the VOOs from the fresh olives. Considering the results of other research [57], we expected that with increased irrigation, the oils would show a fruitier aroma, like that obtained from fruits at a more advanced stage of ripening due to a reduction in their pungent and bitter notes, which was not observed. We also expected that less intense irrigated treatments would improve the oil pungency due to the presence of a higher secoiridoid content, mainly associated with *p*-HPEA-EDA [12]; however, using the Box–Behnken tool, it was not possible to optimize the pungency attribute with the available dataset.

### 3.3. Multivariate Characterization of the Samples Using PCA and PLS-DA

PCA was used to find the statistical differences between the samples as a result of the frost phenomenon. Subsequently, PLS-DA applied to the dataset obtained on the composition of phenolic and volatile compounds, sensory attributes, and color parameters, with a total of 67 variables, was used to discriminate between the VOOs from the fresh and frozen olives.

**Figure 5:**
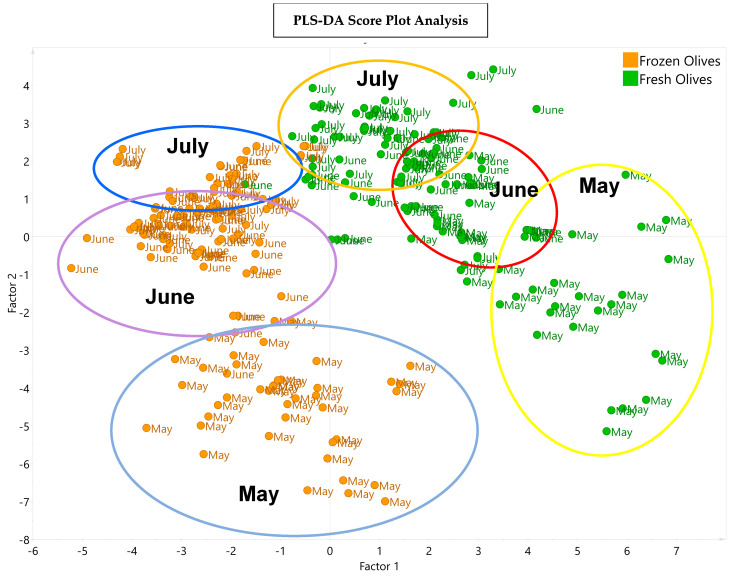
PLS−DA score plot with the distribution of the 15 experiments of Arbequina VOOs obtained from fresh olives (green points) and from frozen olives (orange points) in the corresponding harvesting months of May, June, and July. The explained variance values were 63.6% and 16.1% for factors 1 and 2, respectively.

Figure 5 presents the PLS-DA depicting the distribution of the VOOs obtained from the fresh olives and the frozen olives in the three harvesting months. Factor 1 shows the variation in the samples due to the fresh and frost conditions being applied to the olives, and factor 2 shows the variation in the samples according to harvest month.

The graphic shows that there are two groups of samples that differ according to the phenolic and volatile composition and sensory attributes of their samples; one group corresponds to the early stage of ripening (May), and the other group corresponds to the most advanced stages of ripening (June and July). In addition, a clear difference is observed between the VOOs from the fresh olives and the VOOs from the frozen olives in each harvest month.

**Figure 6:**
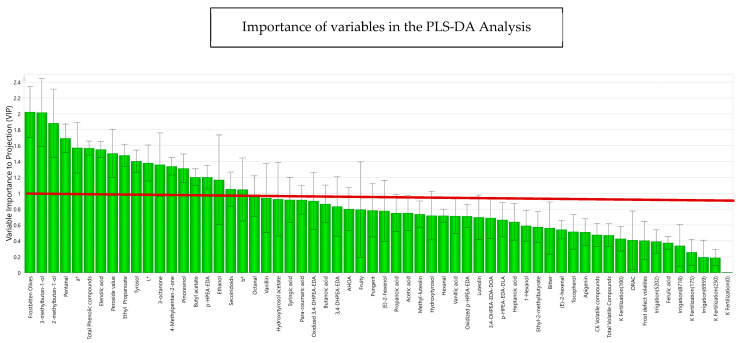
Importance of each variable to the discriminant model (R^2^VY for each compound) for phenolic compounds, volatile compounds, sensory attributes, sensory defects, and design variables, obtained in the PLS-DA. The prediction and adjustment capacity of the model was 88.4%. L*, a*, b* represent the chromatic coordinates of CIELAB colorimetric system.

The variables studied were then analyzed according to multivariate analysis with PLS-DA, classifying the samples depending on whether the oils came from fresh or frozen olives. Figure 6 shows the variables that are important for projection according to the explained variance in the phenomenon for each variable. The green bars in this figure consider the contribution of the variables (R^2^VY) to the model, explaining the degree of variance for each variable in the model. Thus, R^2^VY represents the fraction of X variation which is modeled by Y components [59], ranging from 0 to 1. A R^2^VY value > 0.8 was considered as the selection criterion to select those variables that were well explained, accounted for selecting variables for a descriptive approximation. Most of the variables made a significant contribution to the explanation of the behavior of the variables according to the frost phenomenon, highlighting with a greater strength of correlation phenolic compounds such as *p*-HPEA-EDA and 3,4-DHPEA-EDA, pinoresinol and elenolic acid, and volatile compounds such as 3-methyl-1-butanol, 2-methyl-1-butanol, and pentanal.

**Figure 7:**
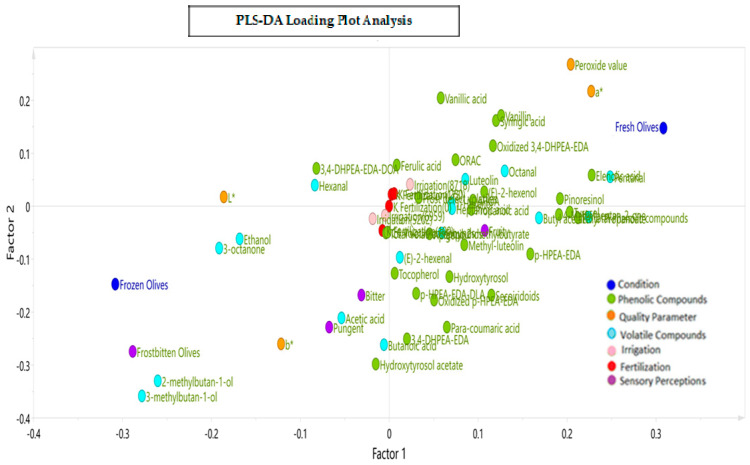
Loading plot resulting from the Partial Least Squares Regression–Discriminant Analysis (PLS−DA) for the three levels of potassium fertilization and irrigation in relation to the phenolic and volatile compounds, sensory attributes and defects, and color parameters in VOOs.

Figure 7 presents the corresponding loading plot of the PLS-DA model with the three levels for the two design variables (irrigation and potassium fertilization) in relation to the different attributes, defects, phenolic and volatile compounds, and color parameters observed across three months of harvest for the VOOs from the fresh and frozen olives. The results show that factor 1 sorts the variables according to the treatments applied and factor 2 according to the design variables of the BBD.

The discrimination and adjustment capacity of the model was 88.4%. The PLS-DA model made it possible to distinguish between the oil samples (loading) from the fresh and frozen olives. Thus, the *x*-axis explains the differentiation between the conditions applied to the olives as a function of the total phenolic compounds, and the *y*-axis explains the contribution of the design variables, fertilization and irrigation, to the dependent variables.

Figure 7 also shows that the concentrations of total phenolic compounds, elenolic acid, pentanal, syringic acid, vanillin, pinoresinol, *p*-HPEA-EDA tyrosol, (E)-2-hexenol, hydroxytyrosol, and secoiridoid compounds, among other things, were positively correlated with the VOOs from the fresh olives. On the other hand, the means of the “frostbitten olives” defect, the bitter and pungent attributes, the values of the L* and b* color parameters, and the concentration of some volatile compounds, such as ethanol, 2-methylbutan-1-ol, 3- methylbutan-1-ol, and hexanal, were positively correlated with the VOOs from the frozen olives. The color parameter a* was also correlated with the VOOs from the frozen olives since its values were negatives (−10.1 vs. −6.9), although the opposite was true for the fresh olive oils since −6.9 is considered a more positive value. Since the color parameter a* has negative values, less negative values of a* were seen for the VOOs from the fresh olives and more negative values were seen for the VOOs from the frozen olives.

Regarding the influence of the design variables on the dependent variables, it was observed that a high irrigation of a 125% ET_0_ (8718 m^3^/ha) and moderate and high fertilization levels (175 and 250 UK_2_O) highly correlated with the variables in the upper-right quadrant, with a greater preponderance being observed for the variables of luteolin, 1-hexanol, (E)-2-hexenol, ferulic and vanillic acids, and octanal. Romero et al. [21] also described increased concentrations of lignans, vanillic acid, and vanillin in oils associated with highly irrigated treatments. An intermediate to low irrigation of a 75 to 100% ET_0_ (5202 to 6950 m^3^/ha) and fertilization of 100 UK_2_O correlated positively with C6 and total volatile compounds, apigenin, ethyl-2-methylbutyrate, total phenolic compounds, butyl acetate and propanoic acid, and AHOA (aldehydic and hydroxylic oleuropein aglycone). Finally, zero fertilization with intermediate to low irrigation correlated positively with phenolic compounds, 3,4-DHPEA-EDA, hydroxytyrosol acetate, *p*-HPEA-EDA, methyl luteolin, hydroxytyrosol, secoiridoid compounds, *p*-coumaric acid, the dialdehydic form of the ligstroside aglycone (*p*-HPEA-EDA-DLA), and the volatiles (E)-2-hexenal and butanoic acid. The “frostbitten olives” defect was strongly associated with 2-methylbutan-1-ol and 3-methylbutan-1-ol. This last compound was described by Romero et al. [60] as a wet wood defect in conjunction with pentanal, octanal, (E)-2-heptenal, acetic acid, and ethyl-2-methylbutyrate. Medium and lower levels of irrigation (a 100 and 75% ET_0_) were also associated with greater fruity, bitter, and pungent sensations.

The bitter attribute was associated with the phenolic compounds *p*-HPEA-EDA-DLA and 3,4-DHPEA-EDA, which, in the literature, have been mainly described as contributors to a bitter taste [9], and to a lesser degree, to pungent attributes. The pungent attribute was associated with the phenolic hydroxytyrosol acetate and the volatiles acetic acid and butanoic acid.

## 4. Conclusions

The “frostbitten olives” defect is perceived in some oils from olives subjected to light frost, being classified as virgin olive oils.

The results of this study showed that a reduced irrigation of an 86% ET_0_ in May and a 100% ET_0_ or higher in July, both associated with fertilization of 100 UK_2_O, could mitigate frost damage, reducing the loss of total phenolic compounds in olive fruits and VOO. However, a 125% ET_0_ irrigation would be required to mitigate the effect of frost damage on the antioxidant capacity at an early stage of ripening. On the other hand, an ET_0_ of 100% or a higher irrigation rate, associated with fertilization of 250 UK_2_O, would be required to minimize the effects of potential frost on the content of total volatile compounds in virgin olive oil. Regarding the volatile compounds, there is still a lack of understanding on the effect that potassium fertilization has on these compounds, and it would be interesting to deepen our knowledge to understand how this affects olive tree fruits.

The PLS-DA allowed us to differentiate between the VOOs according to the ripening stage and the olives’ condition prior to milling (fresh and frozen). The PLS-DA showed the correlation between lower to moderate irrigation (75–100% ET_0_) and fertilization of 100 UK_2_O and a higher concentration of C6 and total volatile compounds and total phenolic compounds. On the other hand, this study also showed that zero fertilization and lower to intermediate irrigation was associated positively with secoiridoid compounds such as *p*-HPEA-EDA, 3,4-DHPEA-EDA, and *p*-HPEA-EDA-DLA and fruity, pungent, and bitter attributes.

When subjecting the olive trees to different agronomic management conditions, complex adaptive physiological responses are triggered, and these responses also depend on climatic factors, causing the results obtained to be highly complex to analyze. Comparison of a univariate approach, such as the BBD, with a multivariate methodology, such as PLS-DA, offered complementary perspectives on analysis of the results for the same problem, so it was interesting to pair them for a comprehensive analysis, improving our understanding of the frost phenomenon, the stage of ripening, irrigation, and potassium fertilization.

However, it is important to note that this type of study is limited by the impossibility of studying all the agronomic factors at the same time or of corroborating the results in other areas or for other olive cultivars, which may lead to slightly different results. On the other hand, frost in olive trees may trigger physiological processes different from those experienced under artificial frost conditions, although, at the same time, natural frost is difficult to reproduce or make predictions for in a complete study in which many variables are studied. All these considerations were taken into account in the design of the experiments.

Due to the adaptive physiological responses that are generated in plant tissues caused by different stimuli and agroclimatic phenomena, it is difficult to predict an optimal irrigation and fertilization treatment that is applicable throughout the phenological period of fruit. It is necessary to adapt these treatments to the specific physiological responses of the crop, especially in periods of rapid growth and fruit development, to guarantee optimum-quality virgin olive oil. In this sense, the recommendation would be to apply precision agronomic management based on the needs of the crop itself, considering geographical and climatic variables, to avoid exerting unnecessary stress on the plants, resulting in damage to the crops and the oils.

## Figures and Tables

**Table 1:** Irrigation, potassium fertilization, and copper oxychloride treatments of each sample.

Box–Behnken Sample	Irrigation (% ET_0_)	Potassium Fertilization (UK_2_O)	Copper Oxychloride (g/100 L of Water)
1	75	100	200
2	125	100	200
3	75	250	200
4	125	250	200
5	75	175	100
6	125	175	100
7	75	175	300
8	125	175	300
9	100	100	100
10	100	250	100
11	100	100	300
12	100	250	300
13	100	175	200
14	100	175	200
15	100	175	200

**Table 2:** Means of quality and color parameters by condition and harvest month.

	Condition	May	June	July
Free fatty acids (% of oleic acid)	Fresh	0.12 ^aA^ ± 0.01	0.10 ^aA^ ± 0.01	0.15 ^bA^ ± 0.01
	Frost	0.15 ^bB^ ± 0.01	0.16 ^bB^ ± 0.01	0.17 ^bcB^ ± 0.01
Peroxide value (mEq O_2_/kg of oil)	Fresh	3.79 ^cA^ ± 0.27	4.20 ^cA^ ± 0.27	3.78 ^cA^ ± 0.29
	Frost	1.96 ^aB^ ± 0.13	3.22 ^bB^ ± 0.14	3.30 ^bB^ ± 0.15
K232	Fresh	2.06 ^bA^ ± 0.04	2.06 ^bB^ ± 0.04	2.22 ^cA^ ± 0.04
	Frost	1.87 ^aB^ ± 0.04	2.18 ^cA^ ± 0.04	2.14 ^bcB^ ± 0.04
K270	Fresh	0.09 ^bA^ ± 0.00	0.09 ^bA^ ± 0.00	0.08 ^aA^ ± 0.00
	Frost	0.08 ^aB^ ± 0.00	0.09 ^bA^ ± 0.00	0.08 ^aA^ ± 0.00
L *	Fresh	86.9 ^aA^ ± 3.46	91.28 ^abA^ ± 3.46	97.65 ^bcA^ ± 3.58
	Frost	98.74 ^cB^ ± 1.19	99.51 ^cB^ ± 1.19	98.41 ^cA^ ± 1.28
a *	Fresh	−7.77 ^aA^ ± 0.65	−8.17 ^aA^ ± 0.65	−7.60 ^aA^ ± 0.67
	Frost	−11.09 ^cB^ ± 0.40	−9.32 ^bB^ ± 0.40	−9.67 ^bB^ ± 0.43
b *	Fresh	34.61 ^bA^ ± 1.74	30.64 ^aA^ ± 1.74	28.06 ^aA^ ± 1.80
	Frost	38.47 ^bcB^ ± 1.68	29.80 ^aA^ ± 1.48	34.87 ^bB^ ± 1.59

* Note: Means were determined using Fisher’s LSD method at the 95% confidence level. Different lowercase letters in the same row indicate significant differences (*p* < 0.05) between harvest months; different capital letters indicate significant differences (*p* < 0.05) between treatments. Abbreviations: Fresh, VOOs from fresh olives; Frost, VOOs from frozen olives.

**Table 3:** Optimal values of irrigation and potassium fertilization in VOOs from fresh and frozen olives per month of harvest, which determine the maximum concentrations of total phenolic compounds and ORAC values.

Total Phenolic Compounds						
Month of Harvest	Condition	Optimal Irrigation (% ET_0_)	Optimal Potassium Fertilization (UK_2_O or kg/ha)	Optimal Phenolic Concentration (mg/kg)	R^2^_adj_% *	MAE **
**May**	Fresh	75	250	251	75.97%	10.35
	Frost	86	100	154	85.31%	3.41
**June**	Fresh	125	100	188	85.99%	5.20
	Frost	125	100	111	83.92%	3.67
**July**	Fresh	125	100	232	88.55%	6.60
	Frost	110	107	156	87.45%	4.93
			**RAC Assay**			
**Month of Harvest**	**Condition**	**Optimal Irrigation (% ET_0_)**	**Optimal Potassium Fertilization (UK_2_O or kg/ha)**	**Optimal ORAC Value (µmol TE/g)**	**R** **^2^** **_adj_** % *	MAE **
**May**	Fresh	75	250	5.58	83.56	0.1702
	Frost	125	100	4.57	88.32	0.0830

Abbreviations: Fresh: VOOs from fresh olives; Frost: VOOs from frozen olives. The predicted optimal concentration of phenols in mg/kg was obtained by applying the optimal values of irrigation and potassium fertilization described in each row. * R^2^_adj_: adjusted R^2^ by degree of freedom, which allows us to see how well the model fits the data; a high percentage is expected. ** MAE: mean absolute error; represents the error of the goodness of fit. An accepted value must be at least 10 times smaller in magnitude than the response variable data.

**Table 4:** Values of irrigation and potassium fertilization in VOOs from fresh and frozen olives per month of harvest, which allow for maximum concentrations of total volatile compounds.

Total Volatile Compounds						
Month of Harvest	Condition	Optimal Irrigation (% ET_0_)	Optimal Potassium Fertilization (UK_2_O)	Volatile Concentration (mg/kg)	R^2^_adj_% *	MAE **
**May**	**Fresh**	121	131	91	90.80	1.96
	**Frost**	125	117	72	71.98	0.96
**June**	**Fresh**	125	250	76	76.34	1.90
	**Frost**	125	250	75	74.98	1.57
**July**	**Fresh**	99	130	63	63.31	0.83
	**Frost**	103	250	72	72.27	1.88

Abbreviations: see Table 3. The predicted highest concentration of volatiles in mg/kg was obtained by applying the optimal values of irrigation and potassium fertilization described in each row. * R^2^_adj_: adjusted R^2^ by degree of freedom, which allows us to see how well the model fits the data; a high percentage is expected. ** MAE: mean absolute error; represents the error of the goodness of fit. An accepted value must be at least 10 times smaller in magnitude than the response variable data.

**Table 5:** Optimal values of irrigation and potassium fertilization in VOOs from fresh and frozen olives for the month of May, which generated maximum values for positive sensory attributes (fruity, pungent, and bitter attributes).

Sensory Attributes for the Month of May						
Descriptor	Condition	Optimal Irrigation (% ET_0_)	Optimal Potassium Fertilization (UK_2_O)	Optimal Sensory Attributes (Means)	R^2^_adj_% *	MAE **
Fruity	Fresh	75	250	4.6	62.16	0.34
	Frost	75	100	3.0	57.08	0.12
Pungency	Fresh ***	-	-	-	-	-
	Frost	75	204	3.4	62.64	0.45
Bitter	Fresh	75	250	3.0	59.64	0.34
	Frost	75	100	2.3	64.50	0.11

Abbreviations: see Table 3. The predicted optimal median level for attributes was obtained by applying the optimal values for irrigation and potassium fertilization described in each row. * R^2^_adj_: adjusted R^2^ by degree of freedom, which allowed us to see how well the model fits the data; a high percentage is expected. ** MAE: mean absolute error; represents the error of the goodness of fit. An accepted value must be at least 10 times smaller in magnitude than the response variable data. *** The model did not optimize the pungent attribute in VOOs from fresh olives.

## Data Availability

The data are contained within the article and Supplementary Materials.

## Supplementary Materials

The following supporting information can be downloaded at https://www.mdpi.com/article/10.3390/antiox13050559/s1. Table S1: Physicochemical and sensory data on virgin olive oils obtained from fresh and frozen olives.
