# Supplementary material for: The Management of Irrigation and Potassium Fertilization to Mitigate the Effect of Light Frosts on the Phenolic and Volatile Compounds in Virgin Olive Oils
Source: Antioxidants (Basel). 2024 May 1;13(5):559. doi: 10.3390/antiox13050559 (PMC11117877; doi:10.3390/antiox13050559)
Supplement: Supplementary file 1 [file antioxidants-13-00559-s001.zip › antioxidants-2909169-supplementary.pdf]

Primary ID	Trat	Date	K Fertilization	Irrigation	Free Acidity	Peroxide value	K232	K270	L*	a*	b*	ORAC	Tocopherol	Elenolic acid	Hydroxytyrosol	Tyrosol	Vanillic acid	Syringic acid	Vanillin	roxytyrosol ace	ra-coumaric ac	Ferulic acid	Tyrosol acetate	3,4-DHPEA-EDA	ized 3,4-DHPEA	-DHPEA-EDA-D	p-HPEA-EDA	dized p-HPEA-E	Pinoresinol	-HPEA-EDA-DL	AHOA	Luteolin	Apigenin	Methyl-luteolin	Phenolic comp	Secoiridoids	Ethanol	thyl Propanoat	Pentanal	ethylpentan-2-
1	Frozen Olive	May	100	5202	0.17923	2.61819	2.31354	0.0919031	95.02	-2.865	38.175	2.66904	148.179	65.3641	0.641688	1.17511	0.375783	0.00588013	0.122647	1.21088	0.488255	0.253301	0.0936064	8.78017	0.315944	0.262614	15.6987	2.07027	26.4671	4.43423	3.30136	2.88733	1.15974	1.10663	136.215	32.4771	0.474597	0.0749059	0.307173	0.126514
2	Frozen Olive	May	100	5202	0.170461	1.51923	1.88169	0.0772762	93.315	-7.7	34.63	3.60676	140.021	65.3641	0.641688	1.17511	0.375783	0.00588013	0.122647	1.21088	0.488255	0.253301	0.0936064	8.78017	0.315944	0.262614	15.6987	2.07027	26.4671	4.43423	3.30136	2.88733	1.15974	1.10663	136.215	32.4771	0.474597	0.0749059	0.307173	0.126514
3	Frozen Olive	May	100	5202	0.133094	1.38778	1.98168	0.0778936	100.205	-9.905	32.21	5.10744	150.593	65.3641	0.641688	1.17511	0.375783	0.00588013	0.122647	1.21088	0.488255	0.253301	0.0936064	8.78017	0.315944	0.262614	15.6987	2.07027	26.4671	4.43423	3.30136	2.88733	1.15974	1.10663	136.215	32.4771	0.474597	0.0749059	0.307173	0.126514
4	Frozen Olive	May	100	5202	0.175483	1.46848	1.89492	0.0934358	99.875	-13.725	46.74	7.43256	183.558	65.3641	0.641688	1.17511	0.375783	0.00588013	0.122647	1.21088	0.488255	0.253301	0.0936064	8.78017	0.315944	0.262614	15.6987	2.07027	26.4671	4.43423	3.30136	2.88733	1.15974	1.10663	136.215	32.4771	0.474597	0.0749059	0.307173	0.126514
5	Frozen Olive	May	175	5202	0.156604	1.85669	1.8564	0.0878735	100.4	-11.68	39.3	2.86397	194.996	60.5626	0.346469	1.09394	0.317029	0.0150979	0.179276	0.784698	0.283549	0.248466	0.0839342	4.46787	0.229305	0.150945	17.8437	2.42709	24.0992	3.99226	0.549056	2.00709	0.929097	0.891723	121.502	27.0038	0.475969	0.109629	0.412114	0.197042
6	Frozen Olive	May	175	5202	0.128431	1.84768	1.93158	0.0913343	100.61	-15.02	53.42	3.69767	159.82	60.5626	0.346469	1.09394	0.317029	0.0150979	0.179276	0.784698	0.283549	0.248466	0.0839342	4.46787	0.229305	0.150945	17.8437	2.42709	24.0992	3.99226	0.549056	2.00709	0.929097	0.891723	121.502	27.0038	0.475969	0.109629	0.412114	0.197042
7	Frozen Olive	May	175	5202	0.142518	1.85219	1.89399	0.0896039	100.505	-13.35	46.36	3.28082	177.408	60.5626	0.346469	1.09394	0.317029	0.0150979	0.179276	0.784698	0.283549	0.248466	0.0839342	4.46787	0.229305	0.150945	17.8437	2.42709	24.0992	3.99226	0.549056	2.00709	0.929097	0.891723	121.502	27.0038	0.475969	0.109629	0.412114	0.197042
8	Frozen Olive	May	175	5202	0.156604	1.85669	1.8564	0.0878735	100.4	-11.68	39.3	2.86397	194.996	60.5626	0.346469	1.09394	0.317029	0.0150979	0.179276	0.784698	0.283549	0.248466	0.0839342	4.46787	0.229305	0.150945	17.8437	2.42709	24.0992	3.99226	0.549056	2.00709	0.929097	0.891723	121.502	27.0038	0.475969	0.109629	0.412114	0.197042
9	Frozen Olive	May	175	5202	0.14685	1.48836	1.74058	0.0748697	100.58	-10.835	36.365	3.35296	152.766	77.7205	0.711781	1.58269	0.310263	0.0072132	0.129674	2.55627	0.337343	0.238951	0.127635	34.4082	1.62601	0.0648649	30.3583	0.709113	25.8048	4.12328	17.7932	3.8392	1.33811	1.18027	204.968	86.7478	0.665664	0.074632	0.206645	0.102407
10	Frozen Olive	May	175	5202	0.148229	1.50541	1.81621	0.0725809	101.645	-10.34	33.185	2.71268	145.645	77.7205	0.711781	1.58269	0.310263	0.0072132	0.129674	2.55627	0.337343	0.238951	0.127635	34.4082	1.62601	0.0648649	30.3583	0.709113	25.8048	4.12328	17.7932	3.8392	1.33811	1.18027	204.968	86.7478	0.665664	0.074632	0.206645	0.102407
11	Frozen Olive	May	175	5202	0.147539	1.49688	1.7784	0.0737253	101.113	-10.5875	34.775	3.03282	149.206	77.7205	0.711781	1.58269	0.310263	0.0072132	0.129674	2.55627	0.337343	0.238951	0.127635	34.4082	1.62601	0.0648649	30.3583	0.709113	25.8048	4.12328	17.7932	3.8392	1.33811	1.18027	204.968	86.7478	0.665664	0.074632	0.206645	0.102407
12	Frozen Olive	May	175	5202	0.147884	1.50115	1.7973	0.0731531	101.379	-10.4637	33.98	2.87275	147.425	77.7205	0.711781	1.58269	0.310263	0.0072132	0.129674	2.55627	0.337343	0.238951	0.127635	34.4082	1.62601	0.0648649	30.3583	0.709113	25.8048	4.12328	17.7932	3.8392	1.33811	1.18027	204.968	86.7478	0.665664	0.074632	0.206645	0.102407
13	Frozen Olive	May	250	5202	0.179403	4.7078	2.43596	0.0872282	88.715	-7.805	39.995	2.69589	127.533	17.0561	0.626867	1.80384	0.662796	0.00348092	0.132887	1.19002	0.276349	0.377048	0.13537	5.51072	0.101212	0.458192	11.3456	1.10369	24.6852	4.13046	2.82695	2.14555	0.59698	0.84107	76.0103	24.2719	1.0579	0.0782213	0.464305	0.177782
14	Frozen Olive	May	250	5202	0.13958	0.856573	1.94147	0.0779368	100.125	-10.165	34.665	3.15748	167.913	17.0561	0.626867	1.80384	0.662796	0.00348092	0.132887	1.19002	0.276349	0.377048	0.13537	5.51072	0.101212	0.458192	11.3456	1.10369	24.6852	4.13046	2.82695	2.14555	0.59698	0.84107	76.0103	24.2719	1.0579	0.0782213	0.464305	0.177782
15	Frozen Olive	May	250	5202	0.159491	2.78219	2.18871	0.0825825	94.42	-8.985	37.33	2.92669	147.723	17.0561	0.626867	1.80384	0.662796	0.00348092	0.132887	1.19002	0.276349	0.377048	0.13537	5.51072	0.101212	0.458192	11.3456	1.10369	24.6852	4.13046	2.82695	2.14555	0.59698	0.84107	76.0103	24.2719	1.0579	0.0782213	0.464305	0.177782
16	Frozen Olive	May	0	6959	0.133874	1.1982	1.89773	0.0744427	100.33	-9.68	32.59	5.10744	148.451	54.8987	1.0053	1.93909	0.181766	0.0146462	0.133815	9.25807	0.442954	0.272902	0.497374	126.313	5.82412	0.92667	55.6757	1.55659	26.3395	3.42088	23.5913	6.07655	1.74718	1.86343	321.98	209.928	0.635676	0.0684703	0.191349	0.103169
17	Frozen Olive	May	0	6959	0.132314	1.57735	2.06564	0.0813445	100.08	-10.13	31.83		152.735	54.8987	1.0053	1.93909	0.181766	0.0146462	0.133815	9.25807	0.442954	0.272902	0.497374	126.313	5.82412	0.92667	55.6757	1.55659	26.3395	3.42088	23.5913	6.07655	1.74718	1.86343	321.98	209.928	0.635676	0.0684703	0.191349	0.103169
18	Frozen Olive	May	0	6959	0.165854	1.53889	1.66571	0.0929544	98.78	-13.64	46.91	7.43256	185.724	54.8987	1.0053	1.93909	0.181766	0.0146462	0.133815	9.25807	0.442954	0.272902	0.497374	126.313	5.82412	0.92667	55.6757	1.55659	26.3395	3.42088	23.5913	6.07655	1.74718	1.86343	321.98	209.928	0.635676	0.0684703	0.191349	0.103169
19	Frozen Olive	May	0	6959	0.185112	1.39807	2.12414	0.0939172	100.97	-13.81	46.57		181.391	54.8987	1.0053	1.93909	0.181766	0.0146462	0.133815	9.25807	0.442954	0.272902	0.497374	126.313	5.82412	0.92667	55.6757	1.55659	26.3395	3.42088	23.5913	6.07655	1.74718	1.86343	321.98	209.928	0.635676	0.0684703	0.191349	0.103169
20	Frozen Olive	May	100	6959	0.154608	1.47621	1.71569	0.070783	102.19	-9.66	29.11	2.80047	128.097	41.0856	0.51231	1.43789	0.173849	0.0211233	0.107603	0.852997	0.254973	0.241566	0.117378	3.53622	0.226786	0.0730555	12.7075	0.600203	22.6745	4.09923	5.49934	3.62529	1.04807	1.15821	100.054	25.9154	0.619153	0.0667013	0.213893	0.0887179
21	Frozen Olive	May	100	6959	0.122073	1.97905	1.77232	0.0790038	103.07	-11.7	39.305	4.53735	132.241	41.0856	0.51231	1.43789	0.173849	0.0211233	0.107603	0.852997	0.254973	0.241566	0.117378	3.53622	0.226786	0.0730555	12.7075	0.600203	22.6745	4.09923	5.49934	3.62529	1.04807	1.15821	100.054	25.9154	0.619153	0.0667013	0.213893	0.0887179
22	Frozen Olive	May	100	6959	0.138341	1.72763	1.744	0.0748934	102.63	-10.68	34.2075	3.66891	130.169	41.0856	0.51231	1.43789	0.173849	0.0211233	0.107603	0.852997	0.254973	0.241566	0.117378	3.53622	0.226786	0.0730555	12.7075	0.600203	22.6745	4.09923	5.49934	3.62529	1.04807	1.15821	100.054	25.9154	0.619153	0.0667013	0.213893	0.0887179
23	Frozen Olive	May	100	6959	0.130207	1.85334	1.75816	0.0769486	102.85	-11.19	36.7562	4.10313	131.205	41.0856	0.51231	1.43789	0.173849	0.0211233	0.107603	0.852997	0.254973	0.241566	0.117378	3.53622	0.226786	0.0730555	12.7075	0.600203	22.6745	4.09923	5.49934	3.62529	1.04807	1.15821	100.054	25.9154	0.619153	0.0667013	0.213893	0.0887179
24	Frozen Olive	May	100	6959	0.120728	2.12963	1.78812	0.0784807	99.805	-11.385	39.32	3.0019	196.882	74.4987	0.417857	1.28939	0.402861	0.00706712	0.137031	1.48581	0.43167	0.274636	0.0365013	9.6009	0.219918	0.240187	14.177	1.93	28.3437	5.19382	3.34495	2.63507	1.17172	1.04868	146.887	32.5568	0.338161	0.0941832	0.424323	0.197155
25	Frozen Olive	May	100	6959	0.151798	1.84831	1.80913	0.085717	98.785	-13.05	45.84	2.68699	209.565	74.4987	0.417857	1.28939	0.402861	0.00706712	0.137031	1.48581	0.43167	0.274636	0.0365013	9.6009	0.219918	0.240187	14.177	1.93	28.3437	5.19382	3.34495	2.63507	1.17172	1.04868	146.887	32.5568	0.338161	0.0941832	0.424323	0.197155
26	Frozen Olive	May	100	6959	0.136263	1.98897	1.79862	0.0820988	99.295	-12.2175	42.58	2.84444	203.223	74.4987	0.417857	1.28939	0.402861	0.00706712	0.137031	1.48581	0.43167	0.274636	0.0365013	9.6009	0.219918	0.240187	14.177	1.93	28.3437	5.19382	3.34495	2.63507	1.17172	1.04868	146.887	32.5568	0.338161	0.0941832	0.424323	0.197155
27	Frozen Olive	May	100	6959	0.14403	1.91864	1.80388	0.0839079	99.04	-12.6337	44.21	2.76572	206.394	74.4987	0.417857	1.28939	0.402861	0.00706712	0.137031	1.48581	0.43167	0.274636	0.0365013	9.6009	0.219918	0.240187	14.177	1.93	28.3437	5.19382	3.34495	2.63507	1.17172	1.04868	146.887	32.5568	0.338161	0.0941832	0.424323	0.197155
28	Frozen Olive	May	175	6959	0.177213	2.08818	2.07754	0.0806883	81.68	-7.6	41.235	2.6279	206.504	49.4302	0.492757	1.1838	0.592417	0.0281057	0.121202	0.829401	0.278189	0.287478	0.223074	4.34399	0.211711	0.526322	9.06553	1.11037	25.3277	3.90631	3.67286	1.4204	0.451385	0.523153	104.026	21.515	0.716917	0.0738508	0.329834	0.111409
29	Frozen Olive	May	175	6959	0.199888	1.88826	2.03856	0.0820236	100.395	-11.145	39.2	2.89287	168.578	49.4302	0.492757	1.1838	0.592417	0.0281057	0.121202	0.829401	0.278189	0.287478	0.223074	4.34399	0.211711	0.526322	9.06553	1.11037	25.3277	3.90631	3.67286	1.4204	0.451385	0.523153	104.026	21.515	0.716917	0.0738508	0.329834	0.111409
30	Frozen Olive	May	175	6959	0.163331	2.46573	1.87475	0.0781488	100.84	-12.565	43.465	2.27409	165.499	49.4302	0.492757	1.1838	0.592417	0.0281057	0.121202	0.829401	0.278189	0.287478	0.223074	4.34399	0.211711	0.526322	9.06553	1.11037	25.3277	3.90631	3.67286	1.4204	0.451385	0.523153	104.026	21.515	0.716917	0.0738508	0.329834	0.111409
31	Frozen Olive	May	175	6959	0.149647	2.24784	1.59301	0.0798509	100.385	-10.87	38.575	2.95413	179.08	49.4302	0.492757	1.1838	0.592417	0.0281057	0.121202	0.829401	0.278189	0.287478	0.223074	4.34399	0.211711	0.526322	9.06553	1.11037	25.3277	3.90631	3.67286	1.4204	0.451385	0.523153	104.026	21.515	0.716917	0.0738508	0.329834	0.111409
32	Frozen Olive	May	175	6959	0.136286	1.46686	1.88502	0.0848468	98.255	-10.84	35.05	4.873	189.164	49.4302	0.492757	1.1838	0.592417	0.0281057	0.121202	0.829401	0.278189	0.287478	0.223074	4.34399	0.211711	0.526322	9.06553	1.11037	25.3277	3.90631	3.67286	1.4204	0.451385	0.523153	104.026	21.515	0.716917	0.0738508	0.329834	0.111409
33	Frozen Olive	May	175	6959	0.163141	1.78438	1.86539	0.0791128	100.8	-10.73	34.215	4.46303	161.95	49.4302	0.492757	1.1838	0.592417	0.0281057	0.121202	0.829401	0.278189	0.287478	0.223074	4.34399	0.211711	0.526322	9.06553	1.11037	25.3277	3.90631	3.67286	1.4204	0.451385	0.523153	104.026	21.515	0.716917	0.0738508	0.329834	0.111409
34	Frozen Olive	May	250	6959	0.154568	2.61547	2.18037	0.0810581	92.085	-8.755	33.4	2.79587	166.246	50.9529	0.370214	1.67868	0.276837	0.0168416	0.113885	0.853576	0.396589	0.250826	0.203168	3.58691	1.11949	0.140569	12.7317	0.851421	23.8961	3.95093	1.79146	3.51265	1.10346	0.972635	108.771	22.2016	1.38251	0.0689638	0.250856	0.110589
35	Frozen Olive	May	250	6959	0.136069	1.89789	1.91469	0.0830426	98.885	-12.355	46.165	2.91922	176.21	50.9529	0.370214	1.67868	0.276837	0.0168416	0.113885	0.853576	0.396589	0.250826	0.203168	3.58691	1.11949	0.140569	12.7317	0.851421	23.8961	3.95093	1.79146	3.51265	1.10346	0.972635	108.771	22.2016	1.38251	0.0689638	0.250856	0.110589
36	Frozen Olive	May	250	6959	0.145318	2.25668	2.04753	0.0820504	95.485	-10.555	39.7825	2.85754	171.228	50.9529	0.370214	1.67868	0.276837	0.0168416	0.113885	0.853576	0.396589	0.250826	0.203168	3.58691	1.11949	0.140569	12.7317	0.851421	23.8961	3.95093	1.79146	3.51265	1.10346	0.972635	108.771	22.2016	1.38251	0.0689638	0.250856	0.110589
37	Frozen Olive	May	250	6959	0.140694	2.07729	1.98111	0.0825465	97.185	-11.455	42.9738	2.88838	173.719	50.9529	0.370214	1.67868	0.276837	0.0168416	0.113885	0.853576	0.396589	0.250826	0.203168	3.58691	1.11949	0.140569	12.7317	0.851421	23.8961	3.95093	1.79146	3.51265	1.10346	0.972635	108.771	22.2016	1.38251	0.0689638	0.250856	0.110589
38	Frozen Olive	May	250	6959	0.129009	1.52943	1.80998	0.0707012	100.05	-10.3975	36.0925	2.64371	213.655	33.6026	0.77418	1.98088	0.625493	0.00552303	0.107476	1.20531	0.42992	0.336657	0.0185467	4.38782	0.335161	0.446725	5.72067	0.848263	27.3124	5.07773	1.87224	2.93322	0.924267	0.944912	89.89	17.5052	0.560193	0.0832284	0.273597	0.17658
39	Frozen Olive	May	250	6959	0.125138	1.48355	1.75568	0.0685802	97.0485	-10.0856	35.0097	2.5644	207.246	33.6026	0.77418	1.98088	0.625493	0.00552303	0.107476	1.20531	0.42992	0.336657	0.0185467	4.38782	0.335161	0.446725	5.72067	0.848263	27.3124	5.07773	1.87224	2.93322	0.924267	0.944912	89.89	17.5052	0.560193	0.0832284	0.273597	0.17658
40	Frozen Olive	May	250	6959	0.133524	1.58296	1.87333	0.0731757	103.552	-10.7614	37.3557	2.73624	221.133	33.6026	0.77418	1.98088	0.625493	0.00552303	0.107476	1.20531	0.42992	0.336657	0.0185467	4.38782	0.335161	0.446725	5.72067	0.848263	27.3124	5.07773	1.87224	2.93322	0.924267	0.944912	89.89	17.5052	0.560193	0.0832284	0.273597	0.17658
41	Frozen Olive	May	250	6959	0.129518	1.53547	1.81713	0.0709805	100.445	-10.4386	36.2351	2.65416	214.499	33.6026	0.77418	1.98088	0.625493	0.00552303	0.107476	1.20531	0.42992	0.336657	0.0185467	4.38782	0.335161	0.446725	5.72067	0.848263	27.3124	5.07773	1.87224	2.93322	0.924267	0.944912	89.89	17.5052	0.560193	0.0832284	0.273597	0.17658
42	Frozen Olive	May	100	8718	0.117605	1.63993	1.66906	0.071947	100.12	-11.175	39.18	3.83051	145.566	42.8201	0.555658	1.36128	0.163496	0.00458928	0.0965508	1.14185	0.31302	0.229458	0.0512894	16.8062	0.598096	0.152817	30.6694	4.20296	25.0852	2.24464	6.68204	4.00988	2.0903	1.44157	140.72	56.5551	0.458026	0.0765134	0.275811	0.116668
43	Frozen Olive	May	100	8718	0.141226	2.10924	1.88879	0.0719928	99.17	-12.435	45.795	3.11447	175.278	42.8201	0.555658	1.36128	0.163496	0.00458928	0.0965508	1.14185	0.31302	0.229458	0.0512894	16.8062	0.598096	0.152817	30.6694	4.20296	25.0852	2.24464	6.68204	4.00988	2.0903	1.44157	140.72	56.5551	0.458026	0.0765134	0.275811	0.116668
44	Frozen Olive	May	100	8718	0.129415	1.87459	1.77892	0.0719699	99.645	-11.805	42.4875	3.47249	160.422	42.8201	0.555658	1.36128	0.163496	0.00458928	0.0965508	1.14185	0.31302	0.229458	0.0512894	16.8062	0.598096	0.152817	30.6694	4.20296	25.0852	2.24464	6.68204	4.00988	2.0903	1.44157	140.72	56.5551	0.458026	0.0765134	0.275811	0.116668
45	Frozen Olive	May	100	8718	0.135321	1.99191	1.83386	0.0719813	99.4075	-12.12	44.1413	3.29348	167.85	42.8201	0.555658	1.36128	0.163496	0.00458928	0.0965508	1.14185	0.31302	0.229458	0.0512894	16.8062	0.598096	0.152817	30.6694	4.20296	25.0852	2.24464	6.68204	4.00988	2.0903	1.44157	140.72	56.5551	0.458026	0.0765134	0.275811	0.116668
46	Frozen Olive	May	175	8718	0.113255	2.53825	1.7106	0.0713427	100.71	-12.28	43.86	3.93632	183.117	34.7581	0.285296	1.52713	0.856553	0.00935901	0.152622	1.14635	0.287119	0.377342	0.0484062	4.86565	0.24842	0.433251	6.3337	1.97839	23.9854	5.80456	0.978256	1.44763	0.610635	0.748465	86.8826	18.4154	1.45878	0.0957581	0.769708	0.176985
47	Frozen Olive	May	175	8718	0.204247	1.46778	1.62797	0.0752515	100.715	-10.38	34.065	4.17547	174.078	34.7581	0.285296	1.52713	0.856553	0.00935901	0.152622	1.14635	0.287119	0.377342	0.0484062	4.86565	0.24842	0.433251	6.3337	1.97839	23.9854	5.80456	0.978256	1.44763	0.610635	0.748465	86.8826	18.4154	1.45878	0.0957581	0.769708	0.176985
48	Frozen Olive	May	175	8718	0.158751	2.00302	1.66929	0.0732971	100.713	-11.33	38.9625	4.05589	178.598	34.7581	0.285296	1.52713	0.856553	0.00935901	0.152622	1.14635	0.287119	0.377342	0.0484062	4.86565	0.24842	0.433251	6.3337	1.97839	23.9854	5.80456	0.978256	1.44763	0.610635	0.748465	86.8826	18.4154	1.45878	0.0957581	0.769708	0.176985
49	Frozen Olive	May	175	8718	0.181499	1.7354	1.64863	0.0742743	100.714	-10.855	36.5137	4.11568	176.338	34.7581	0.285296	1.52713	0.856553	0.00935901	0.152622	1.14635	0.287119	0.377342	0.0484062	4.86565	0.24842	0.433251	6.3337	1.97839	23.9854	5.80456	0.978256	1.44763	0.610635	0.748465	86.8826	18.4154	1.45878	0.0957581	0.769708	0.176985
50	Frozen Olive	May	175	8718	0.126567	1.88617	1.63505	0.0837189	101.72	-12.6	39.9	2.41892	190.551	18.3363	0.325531	1.29012	1.10113	0.00782402	0.159291	0.70063	0.160691	0.307632	0.114314	2.8727	0.0962335	0.389641	3.69011	0.477243	23.4432	6.45476	1.47332	1.50762	0.735318	0.812665	64.4563	14.8805	0.457503	0.0928246	0.37543	0.252255
51	Frozen Olive	May	175	8718	0.168409	1.777	1.66779	0.0768911	99.535	-13.255	47.73	2.45635	173.194	18.3363	0.325531	1.29012	1.10113	0.00782402	0.159291	0.70063	0.160691	0.307632	0.114314	2.8727	0.0962335	0.389641	3.69011	0.477243	23.4432	6.45476	1.47332	1.50762	0.735318	0.812665	64.4563	14.8805	0.457503	0.0928246	0.37543	0.252255
52	Frozen Olive	May	175	8718	0.147488	1.83159	1.65142	0.080305	100.628	-12.9275	43.815	2.43763	181.872	18.3363	0.325531	1.29012	1.10113	0.00782402	0.159291	0.70063	0.160691	0.307632	0.114314	2.8727	0.0962335	0.389641	3.69011	0.477243	23.4432	6.45476	1.47332	1.50762	0.735318	0.812665	64.4563	14.8805	0.457503	0.0928246	0.37543	0.252255
53	Frozen Olive	May	175	8718	0.157948	1.80429	1.65961	0.0785981	100.081	-13.0913	45.7725	2.44699	177.533	18.3363	0.325531	1.29012	1.10113	0.00782402	0.159291	0.70063	0.160691	0.307632	0.114314	2.8727	0.0962335	0.389641	3.69011	0.477243	23.4432	6.45476	1.47332	1.50762	0.735318	0.812665	64.4563	14.8805	0.457503	0.0928246	0.37543	0.252255
54	Frozen Olive	May	250	8718	0.152174	2.16314	1.737	0.0829074	99.96	-11.58	38.865	2.83048	179.526	24.965	0.546484	1.36226	0.879839	0.00937283	0.192857	0.888758	0.388444	0.397338	0.234622	5.28536	0.119617	0.592784	10.6771	2.43634	26.4739	5.38943	0.937725	1.53184	0.474481	0.708197	84.4917	22.8824	1.36698	0.0939765	0.528928	0.305238
55	Frozen Olive	May	250	8718	0.181196	3.51032	1.88823	0.0835349	101.805	-10.355	33.92	2.99457	185.589	24.965	0.546484	1.36226	0.879839	0.00937283	0.192857	0.888758	0.388444	0.397338	0.234622	5.28536	0.119617	0.592784	10.6771	2.43634	26.4739	5.38943	0.937725	1.53184	0.474481	0.708197	84.4917	22.8824	1.36698	0.0939765	0.528928	0.305238
56	Frozen Olive	May	250	8718	0.166685	2.83673	1.81262	0.0832212	100.882	-10.9675	36.3925	2.91252	182.557	24.965	0.546484	1.36226	0.879839	0.00937283	0.192857	0.888758	0.388444	0.397338	0.234622	5.28536	0.119617	0.592784	10.6771	2.43634	26.4739	5.38943	0.937725	1.53184	0.474481	0.708197	84.4917	22.8824	1.36698	0.0939765	0.528928	0.305238
57	Frozen Olive	May	250	8718	0.17394	3.17353	1.85042	0.083378	101.344	-10.6613	35.1563	2.95355	184.073	24.965	0.546484	1.36226	0.879839	0.00937283	0.192857	0.888758	0.388444	0.397338	0.234622	5.28536	0.119617	0.592784	10.6771	2.43634	26.4739	5.38943	0.937725	1.53184	0.474481	0.708197	84.4917	22.8824	1.36698	0.0939765	0.528928	0.305238
58	Frozen Olive	June	100	5202	0.131766	1.77803	2.20722	0.0896248	101.685	-9.45	30.085	3.88206	154.787	29.0474	0.310377	0.971236	0.503917	0.0112916	0.12422	0.543963	0.0945898	0.232261	0.16024	1.05751	0.316622	0.194242	4.38391	0.0716419	20.3502	2.89473	2.32033	1.65028	0.542413	0.593632	66.3751	10.8507	0.374531	0.0664437	0.120739	0.10153
59	Frozen Olive	June	100	5202	0.0765414	1.10891	1.82923	0.077531	101.73	-8.725	27.135	4.00576	155.761	29.0474	0.310377	0.971236	0.503917	0.0112916	0.12422	0.543963	0.0945898	0.232261	0.16024	1.05751	0.316622	0.194242	4.38391	0.0716419	20.3502	2.89473	2.32033	1.65028	0.542413	0.593632	66.3751	10.8507	0.374531	0.0664437	0.120739	0.10153
60	Frozen Olive	June	100	5202	0.104154	1.44347	2.01822	0.0835779	101.707	-9.0875	28.61	3.94391	155.274	29.0474	0.310377	0.971236	0.503917	0.0112916	0.12422	0.543963	0.0945898	0.232261	0.16024	1.05751	0.316622	0.194242	4.38391	0.0716419	20.3502	2.89473	2.32033	1.65028	0.542413	0.593632	66.3751	10.8507	0.374531	0.0664437	0.120739	0.10153
61	Frozen Olive	June	100	5202	0.0903476	1.27619	1.92373	0.0805544	101.719	-8.90625	27.8725	3.97483	155.518	29.0474	0.310377	0.971236	0.503917	0.0112916	0.12422	0.543963	0.0945898	0.232261	0.16024	1.05751	0.316622	0.194242	4.38391	0.0716419	20.3502	2.89473	2.32033	1.65028	0.542413	0.593632	66.3751	10.8507	0.374531	0.0664437	0.120739	0.10153
62	Frozen Olive	June	175	5202	0.167738	3.79859	2.39719	0.094849	97.515	-7.94	24.715	3.94285	141.376	24.2559	0.293827	0.86274	0.218457	0.0118193	0.107203	0.532283	0.0615559	0.293307	0.0166014	0.710427	0.118902	0.283382	1.63875	0.133902	20.9558	1.00527	1.79141	3.67524	1.26616	1.12029	59.3532	5.42924	0.754193	0.0617344	0.148017	0.10159
63	Frozen Olive	June	175	5202	0.263465	3.45668	2.18326	0.0938946	101.545	-7.7	21.92	3.85697	166.402	24.2559	0.293827	0.86274	0.218457	0.0118193	0.107203	0.532283	0.0615559	0.293307	0.0166014	0.710427	0.118902	0.283382	1.63875	0.133902	20.9558	1.00527	1.79141	3.67524	1.26616	1.12029	59.3532	5.42924	0.754193	0.0617344	0.148017	0.10159
64	Frozen Olive	June	175	5202	0.215602	3.62764	2.29023	0.0943718	99.53	-7.82	23.3175	3.89991	153.889	24.2559	0.293827	0.86274	0.218457	0.0118193	0.107203	0.532283	0.0615559	0.293307	0.0166014	0.710427	0.118902	0.283382	1.63875	0.133902	20.9558	1.00527	1.79141	3.67524	1.26616	1.12029	59.3532	5.42924	0.754193	0.0617344	0.148017	0.10159
65	Frozen Olive	June	175	5202	0.239533	3.54216	2.23674	0.0941332	100.537	-7.76	22.6187	3.87844	160.145	24.2559	0.293827	0.86274	0.218457	0.0118193	0.107203	0.532283	0.0615559	0.293307	0.0166014	0.710427	0.118902	0.283382	1.63875	0.133902	20.9558	1.00527	1.79141	3.67524	1.26616	1.12029	59.3532	5.42924	0.754193	0.0617344	0.148017	0.10159
66	Frozen Olive	June	175	5202	0.204815	3.25491	2.24704	0.079014	97.69	-9.75	32.96	2.93518	131.707	18.7978	0.461129	1.05362	0.287708	0.0159245	0.1156	0.541142	0.160421	0.241648	0.145526	1.36388	0.509355	0.29383	5.22795	0.0611074	21.7311	2.84576	5.04538	4.75324	1.56308	1.19296	66.4081	14.7768	0.439227	0.0695428	0.162947	0.132674
67	Frozen Olive	June	175	5202	0.222174	3.39853	2.24189	0.0865736	99.1137	-8.755	27.7894	3.40681	145.926	18.7978	0.461129	1.05362	0.287708	0.0159245	0.1156	0.541142	0.160421	0.241648	0.145526	1.36388	0.509355	0.29383	5.22795	0.0611074	21.7311	2.84576	5.04538	4.75324	1.56308	1.19296	66.4081	14.7768	0.439227	0.0695428	0.162947	0.132674
68	Frozen Olive	June	175	5202	0.213495	3.32672	2.24447	0.0827938	98.4019	-9.2525	30.3747	3.17099	138.817	18.7978	0.461129	1.05362	0.287708	0.0159245	0.1156	0.541142	0.160421	0.241648	0.145526	1.36388	0.509355	0.29383	5.22795	0.0611074	21.7311	2.84576	5.04538	4.75324	1.56308	1.19296	66.4081	14.7768	0.439227	0.0695428	0.162947	0.132674
69	Frozen Olive	June	175	5202	0.13357	3.22173	2.23622	0.0765962	97.66	-9.795	28.19	3.21856	199.237	18.7978	0.461129	1.05362	0.287708	0.0159245	0.1156	0.541142	0.160421	0.241648	0.145526	1.36388	0.509355	0.29383	5.22795	0.0611074	21.7311	2.84576	5.04538	4.75324	1.56308	1.19296	66.4081	14.7768	0.439227	0.0695428	0.162947	0.132674
70	Frozen Olive	June	250	5202	0.133381	2.83776	2.1251	0.0768505	100.51	-8.515	26.265	2.49667	136.694																											
71	Frozen Olive	June	0	6959	0.110139	2.3591	1.82624	0.0890356	102.52	-11.96	35.68	5.04303	124.185	55.6438	0.416733	1.0503	0.160668	0.0162862	0.100077	0.611163	0.203472	0.233613	0.422841	1.13406	0.0810719	0.211485	11.2463	0.137046	21.7867	2.69141	6.54272	5.75359	1.78467	1.52109	111.749	21.826	0.647842	0.0717756	0.244926	0.162335
72	Frozen Olive	June	0	6959	0.101915	2.45602	1.9771	0.0960586	98.52	-10.89	34.91		121.191	55.6438	0.416733	1.0503	0.160668	0.0162862	0.100077	0.611163	0.203472	0.233613	0.422841	1.13406	0.0810719	0.211485	11.2463	0.137046	21.7867	2.69141	6.54272	5.75359	1.78467	1.52109	111.749	21.826	0.647842	0.0717756	0.244926	0.162335
73	Frozen Olive	June	0	6959	0.138742	2.2383	2.04	0.0961003	102.89	-11.3	35.18	5.1701	131.18	55.6438	0.416733	1.0503	0.160668	0.0162862	0.100077	0.611163	0.203472	0.233613	0.422841	1.13406	0.0810719	0.211485	11.2463	0.137046	21.7867	2.69141	6.54272	5.75359	1.78467	1.52109	111.749	21.826	0.647842	0.0717756	0.244926	0.162335
74	Frozen Olive	June	0	6959	0.12736	2.35468	2.09434	0.0871205	99.31	-10.44	34.98		121.02	55.6438	0.416733	1.0503	0.160668	0.0162862	0.100077	0.611163	0.203472	0.233613	0.422841	1.13406	0.0810719	0.211485	11.2463	0.137046	21.7867	2.69141	6.54272	5.75359	1.78467	1.52109	111.749	21.826	0.647842	0.0717756	0.244926	0.162335
75	Frozen Olive	June	0	6959	0.133051	2.29649	2.06717	0.0916104	101.1	-10.87	35.08	5.1701	126.1	55.6438	0.416733	1.0503	0.160668	0.0162862	0.100077	0.611163	0.203472	0.233613	0.422841	1.13406	0.0810719	0.211485	11.2463	0.137046	21.7867	2.69141	6.54272	5.75359	1.78467	1.52109	111.749	21.826	0.647842	0.0717756	0.244926	0.162335
76	Frozen Olive	June	100	6959	0.151814	3.77238	2.33529	0.0944489	99.54	-11.415	32.385	3.09244	150.96	23.7816	0.579934	1.17437	0.414797	0.0183368	0.13451	0.899888	0.23139	0.243344	0.0301932	2.72331	0.880628	0.25724	6.27991	0.14853	21.8038	3.31711	2.71098	3.36836	1.40988	1.00056	71.4086	15.2885	0.400957	0.0830664	0.138959	0.172236
77	Frozen Olive	June	100	6959	0.138413	3.69442	2.34619	0.0878107	100.768	-11.74	36.4125	3.17006	145.583	23.7816	0.579934	1.17437	0.414797	0.0183368	0.13451	0.899888	0.23139	0.243344	0.0301932	2.72331	0.880628	0.25724	6.27991	0.14853	21.8038	3.31711	2.71098	3.36836	1.40988	1.00056	71.4086	15.2885	0.400957	0.0830664	0.138959	0.172236
78	Frozen Olive	June	100	6959	0.131712	3.65543	2.35164	0.0844916	101.381	-11.9025	38.4263	3.20888	142.895	23.7816	0.579934	1.17437	0.414797	0.0183368	0.13451	0.899888	0.23139	0.243344	0.0301932	2.72331	0.880628	0.25724	6.27991	0.14853	21.8038	3.31711	2.71098	3.36836	1.40988	1.00056	71.4086	15.2885	0.400957	0.0830664	0.138959	0.172236
79	Frozen Olive	June	100	6959	0.125012	3.61645	2.35709	0.0811725	101.995	-12.065	40.44	3.24769	140.206	23.7816	0.579934	1.17437	0.414797	0.0183368	0.13451	0.899888	0.23139	0.243344	0.0301932	2.72331	0.880628	0.25724	6.27991	0.14853	21.8038	3.31711	2.71098	3.36836	1.40988	1.00056	71.4086	15.2885	0.400957	0.0830664	0.138959	0.172236
80	Frozen Olive	June	100	6959	0.14254	2.9373	1.8994	0.0780543	101.615	-7.18	20.805	5.61768	127.244	5.18989	0.441324	0.997378	0.782897	0.00973978	0.143973	0.815455	0.0488707	0.30455	0.0307456	2.88339	0.202688	0.398328	1.64094	0.07421	20.8898	3.06731	2.06406	1.47674	0.606734	0.610863	42.6798	10.054	0.408569	0.0659103	0.136122	0.109094
81	Frozen Olive	June	100	6959	0.148586	3.88103	2.05847	0.0910107	100.845	-7.765	24.0175	5.94147	128.753	5.18989	0.441324	0.997378	0.782897	0.00973978	0.143973	0.815455	0.0488707	0.30455	0.0307456	2.88339	0.202688	0.398328	1.64094	0.07421	20.8898	3.06731	2.06406	1.47674	0.606734	0.610863	42.6798	10.054	0.408569	0.0659103	0.136122	0.109094
82	Frozen Olive	June	100	6959	0.145563	3.40916	1.97893	0.0845325	101.23	-7.4725	22.4112	5.77958	127.998	5.18989	0.441324	0.997378	0.782897	0.00973978	0.143973	0.815455	0.0488707	0.30455	0.0307456	2.88339	0.202688	0.398328	1.64094	0.07421	20.8898	3.06731	2.06406	1.47674	0.606734	0.610863	42.6798	10.054	0.408569	0.0659103	0.136122	0.109094
83	Frozen Olive	June	100	6959	0.154632	4.82476	2.21753	0.103967	100.075	-8.35	27.23	6.26527	130.262	5.18989	0.441324	0.997378	0.782897	0.00973978	0.143973	0.815455	0.0488707	0.30455	0.0307456	2.88339	0.202688	0.398328	1.64094	0.07421	20.8898	3.06731	2.06406	1.47674	0.606734	0.610863	42.6798	10.054	0.408569	0.0659103	0.136122	0.109094
84	Frozen Olive	June	175	6959	0.244585	3.41918	2.26336	0.109494	97.42	-8.885	30.155	3.36652	178.464	11.2528	0.228747	0.739376	0.260379	0.00445176	0.0992365	0.533319	0.0108327	0.368508	0.016347	0.937282	0.156588	0.343043	0.810051	0.170352	21.3017	2.68691	2.60453	1.2538	0.401203	0.665196	44.8447	7.38182	0.81231	0.0572942	0.106602	0.0591562
85	Frozen Olive	June	175	6959	0.164103	2.18473	2.03244	0.0930977	99.975	-9.35	28.575	3.80184	144.614	8.86923	0.314389	0.775504	0.261745	0.00835267	0.103807	0.800241	0.0242814	0.594159	0.0219799	0.461767	0.0930746	0.255262	0.908488	0.325016	19.5772	3.39276	2.70473	1.45049	0.457008	0.536753	41.9362	7.723	1.01677	0.0572605	0.138666	0.0757227
86	Frozen Olive	June	175	6959	0.214237	3.82414	2.16088	0.0837056	104.89	-7.635	22.65	4.45196	148.829	48.3522	0.262826	0.874227	0.187342	0.0105852	0.112973	0.534908	0.0443752	0.27985	0.0531305	0.962239	0.047649	0.0904961	3.83111	0.32783	26.9457	2.17527	2.29321	1.30403	0.355341	0.631077	89.6764	9.35233	0.7335	0.064363	0.163517	0.0870671
87	Frozen Olive	June	175	6959	0.218192	3.18339	2.05691	0.0802566	105.475	-8.495	24.545	4.00191	148.76	33.1957	0.277265	0.956867	0.209785	0.0075489	0.111843	0.526094	0.0348535	0.259589	0.0267622	0.824656	1.12221	0.221873	3.66487	0.0965693	24.0106	2.75039	3.07665	2.54728	0.66358	1.00009	75.5851	10.5384	0.465677	0.067101	0.166904	0.0912395
88	Frozen Olive	June	175	6959	0.12891	3.47705	2.31401	0.0901781	99.77	-8.14	25.035	2.84281	151.649	7.30234	0.497247	1.27108	1.00632	0.0137286	0.136785	0.750982	0.170849	0.372846	0.0845987	2.34854	0.0649245	0.384888	2.62075	0.29768	21.0778	3.17025	2.32056	1.99321	0.693589	0.684419	47.2634	10.845	0.407807	0.0692483	0.214835	0.165695
89	Frozen Olive	June	175	6959	0.10406	3.0588	2.17065	0.0900672	101.005	-11.3	37.93	2.88191	141.816	7.43015	0.683784	1.33434	1.09035	0.00521893	0.153181	0.838789	0.181841	0.312126	0.119232	2.8843	0.122925	0.365587	2.9023	0.274635	23.264	3.40195	1.26555	0.816511	0.306845	0.457983	48.2116	10.8197	0.32882	0.066506	0.20569	0.178612
90	Frozen Olive	June	250	6959	0.113874	3.00262	2.17181	0.0783849	101.785	-13.395	45.29	5.4103	120.774	17.4531	0.263044	0.806951	0.210846	0.00525246	0.0946126	0.535645	0.0343571	0.254167	0.0184528	0.4664	0.353554	0.205932	2.47682	0.064185	22.885	2.83663	2.43783	2.07535	0.515651	0.785141	54.7789	8.42361	0.3899	0.0612602	0.165589	0.0795378
91	Frozen Olive	June	250	6959	0.154446	3.23635	2.21746	0.0791567	100.27	-9.685	32.285	4.86827	129.133	17.4531	0.263044	0.806951	0.210846	0.00525246	0.0946126	0.535645	0.0343571	0.254167	0.0184528	0.4664	0.353554	0.205932	2.47682	0.064185	22.885	2.83663	2.43783	2.07535	0.515651	0.785141	54.7789	8.42361	0.3899	0.0612602	0.165589	0.0795378
92	Frozen Olive	June	250	6959	0.13416	3.11948	2.19463	0.0787708	101.027	-11.54	38.7875	5.13929	124.953	17.4531	0.263044	0.806951	0.210846	0.00525246	0.0946126	0.535645	0.0343571	0.254167	0.0184528	0.4664	0.353554	0.205932	2.47682	0.064185	22.885	2.83663	2.43783	2.07535	0.515651	0.785141	54.7789	8.42361	0.3899	0.0612602	0.165589	0.0795378
93	Frozen Olive	June	250	6959	0.144303	3.17792	2.20605	0.0789637	100.649	-10.6125	35.5363	5.00378	127.043	17.4531	0.263044	0.806951	0.210846	0.00525246	0.0946126	0.535645	0.0343571	0.254167	0.0184528	0.4664	0.353554	0.205932	2.47682	0.064185	22.885	2.83663	2.43783	2.07535	0.515651	0.785141	54.7789	8.42361	0.3899	0.0612602	0.165589	0.0795378
94	Frozen Olive	June	250	6959	0.106337	2.50577	1.88933	0.0856663	99.79	-10.52	34.48	6.54216	170.208	10.7372	0.249307	0.833797	0.583625	0.0110203	0.10414	0.54491	0.0498488	0.398223	0.0256896	1.89512	0.0914399	0.364011	0.90434	0.263099	19.6479	3.27202	2.76679	2.39342	0.685878	0.978643	46.8004	9.20228	1.144	0.0610256	0.148567	0.111881
95	Frozen Olive	June	250	6959	0.205732	1.40545	1.09723	0.195397	50.0476	-5.10744	17.3926	3.42364	85.2566	10.7372	0.249307	0.833797	0.583625	0.0110203	0.10414	0.54491	0.0498488	0.398223	0.0256896	1.89512	0.0914399	0.364011	0.90434	0.263099	19.6479	3.27202	2.76679	2.39342	0.685878	0.978643	46.8004	9.20228	1.144	0.0610256	0.148567	0.111881
96	Frozen Olive	June	250	6959	0.25543	0.855289	0.701178	0.250263	25.1763	-2.40115	8.84885	1.86439	42.7808	10.7372	0.249307	0.833797	0.583625	0.0110203	0.10414	0.54491	0.0498488	0.398223	0.0256896	1.89512	0.0914399	0.364011	0.90434	0.263099	19.6479	3.27202	2.76679	2.39342	0.685878	0.978643	46.8004	9.20228	1.144	0.0610256	0.148567	0.111881
97	Frozen Olive	June	250	6959	0.305128	0.305128	0.305128	0.305128	0.305128	0.305128	0.305128	0.305128	0.305128	10.7372	0.249307	0.833797	0.583625	0.0110203	0.10414	0.54491	0.0498488	0.398223	0.0256896	1.89512	0.0914399	0.364011	0.90434	0.263099	19.6479	3.27202	2.76679	2.39342	0.685878	0.978643	46.8004	9.20228	1.144	0.0610256	0.148567	0.111881
98	Frozen Olive	June	100	8718	0.180288	2.9455	2.12904	0.0895074	101.35	-9.12	27.86	3.94082	154.18	35.7089	0.317012	0.894084	0.608348	0.0109066	0.106691	0.551694	0.0798831	0.314026	0.0219289	1.10769	0.0426152	0.288714	1.17997	0.219999	22.5348	3.31369	1.67298	1.44201	0.46222	0.64346	71.5216	7.56303	0.37481	0.0731255	0.133057	0.137338
99	Frozen Olive	June	100	8718	0.119343	3.09429	2.17341	0.0844641	101.88	-9.875	32.285	2.84192	129.397	35.7089	0.317012	0.894084	0.608348	0.0109066	0.106691	0.551694	0.0798831	0.314026	0.0219289	1.10769	0.0426152	0.288714	1.17997	0.219999	22.5348	3.31369	1.67298	1.44201	0.46222	0.64346	71.5216	7.56303	0.37481	0.0731255	0.133057	0.137338
100	Frozen Olive	June	100	8718	0.149815	3.0199	2.15123	0.0869858	101.615	-9.4975	30.0725	3.39137	141.788	35.7089	0.317012	0.894084	0.608348	0.0109066	0.106691	0.551694	0.0798831	0.314026	0.0219289	1.10769	0.0426152	0.288714	1.17997	0.219999	22.5348	3.31369	1.67298	1.44201	0.46222	0.64346	71.5216	7.56303	0.37481	0.0731255	0.133057	0.137338
101	Frozen Olive	June	100	8718	0.134579	3.05709	2.16232	0.0857249	101.747	-9.68625	31.1787	3.11665	135.593	35.7089	0.317012	0.894084	0.608348	0.0109066	0.106691	0.551694	0.0798831	0.314026	0.0219289	1.10769	0.0426152	0.288714	1.17997	0.219999	22.5348	3.31369	1.67298	1.44201	0.46222	0.64346	71.5216	7.56303	0.37481	0.0731255	0.133057	0.137338
102	Frozen Olive	June	100	8718	0.106027	2.40756	1.90167	0.0925471	100.52	-11.425	35.295	5.04303	122.688	35.7089	0.317012	0.894084	0.608348	0.0109066	0.106691	0.551694	0.0798831	0.314026	0.0219289	1.10769	0.0426152	0.288714	1.17997	0.219999	22.5348	3.31369	1.67298	1.44201	0.46222	0.64346	71.5216	7.56303	0.37481	0.0731255	0.133057	0.137338
103	Frozen Olive	June	100	8718	0.133051	2.29649	2.06717	0.0916104	101.1	-10.87	35.08	5.1701	126.1	35.7089	0.317012	0.894084	0.608348	0.0109066	0.106691	0.551694	0.0798831	0.314026	0.0219289	1.10769	0.0426152	0.288714	1.17997	0.219999	22.5348	3.31369	1.67298	1.44201	0.46222	0.64346	71.5216	7.56303	0.37481	0.0731255	0.133057	0.137338
104	Frozen Olive	June	100	8718	0.119539	2.35203	1.98442	0.0920788	100.81	-11.1475	35.1875	5.10656	124.394	35.7089	0.317012	0.894084	0.608348	0.0109066	0.106691	0.551694	0.0798831	0.314026	0.0219289	1.10769	0.0426152	0.288714	1.17997	0.219999	22.5348	3.31369	1.67298	1.44201	0.46222	0.64346	71.5216	7.56303	0.37481	0.0731255	0.133057	0.137338
105	Frozen Olive	June	100	8718	0.126295	2.32426	2.02579	0.0918446	100.955	-11.0087	35.1338	5.13833	125.247	35.7089	0.317012	0.894084	0.608348	0.0109066	0.106691	0.551694	0.0798831	0.314026	0.0219289	1.10769	0.0426152	0.288714	1.17997	0.219999	22.5348	3.31369	1.67298	1.44201	0.46222	0.64346	71.5216	7.56303	0.37481	0.0731255	0.133057	0.137338
106	Frozen Olive	June	175	8718	0.198676	2.48831	2.08816	0.0735526	100.785	-10.56	34.98	2.79694	134.886	20.5552	0.31705	0.994041	0.493929	0.0128474	0.136038	0.583219	0.149376	0.280647	0.0638851	1.08481	0.0820457	0.381664	2.62516	0.0567138	21.5114	3.30766	3.82162	2.6552	1.04467	0.80105	60.9583	11.2209	0.596328	0.0684996	0.155535	0.116724
107	Frozen Olive	June	175	8718	0.181178	3.42501	2.24498	0.0837008	99.515	-10.555	33.725	2.7351	137.982	20.5552	0.31705	0.994041	0.493929	0.0128474	0.136038	0.583219	0.149376	0.280647	0.0638851	1.08481	0.0820457	0.381664	2.62516	0.0567138	21.5114	3.30766	3.82162	2.6552	1.04467	0.80105	60.9583	11.2209	0.596328	0.0684996	0.155535	0.116724
108	Frozen Olive	June	175	8718	0.189927	2.95666	2.16657	0.0786267	100.15	-10.5575	34.3525	2.76602	136.434	20.5552	0.31705	0.994041	0.493929	0.0128474	0.136038	0.583219	0.149376	0.280647	0.0638851	1.08481	0.0820457	0.381664	2.62516	0.0567138	21.5114	3.30766	3.82162	2.6552	1.04467	0.80105	60.9583	11.2209	0.596328	0.0684996	0.155535	0.116724
109	Frozen Olive	June	175	8718	0.185552	3.19083	2.20578	0.0811637	99.8325	-10.5562	34.0387	2.75056	137.208	20.5552	0.31705	0.994041	0.493929	0.0128474	0.136038	0.583219	0.149376	0.280647	0.0638851	1.08481	0.0820457	0.381664	2.62516	0.0567138	21.5114	3.30766	3.82162	2.6552	1.04467	0.80105	60.9583	11.2209	0.596328	0.0684996	0.155535	0.116724
110	Frozen Olive	June	175	8718	0.159052	2.76745	2.16058	0.0758417	99.035	-8.01	25.175	3.31584	118.976	42.0897	0.470324	1.06239	0.280206	0.0096664	0.142078	0.534847	0.0568443	0.232324	0.0507926	0.945407	0.557319	0.192755	5.27673	0.242584	21.8099	2.44307	3.30264	2.34061	0.670002	0.832251	83.5424	12.1606	0.50508	0.0718232	0.133544	0.130392
111	Frozen Olive	June	175	8718	0.133539	4.79767	2.33164	0.0987113	97.63	-10.095	37.505	3.52043	140.954	42.0897	0.470324	1.06239	0.280206	0.0096664	0.142078	0.534847	0.0568443	0.232324	0.0507926	0.945407	0.557319	0.192755	5.27673	0.242584	21.8099	2.44307	3.30264	2.34061	0.670002	0.832251	83.5424	12.1606	0.50508	0.0718232	0.133544	0.130392
112	Frozen Olive	June	175	8718	0.146296	3.78256	2.24611	0.0872765	98.3325	-9.0525	31.34	3.41813	129.965	42.0897	0.470324	1.06239	0.280206	0.0096664	0.142078	0.534847	0.0568443	0.232324	0.0507926	0.945407	0.557319	0.192755	5.27673	0.242584	21.8099	2.44307	3.30264	2.34061	0.670002	0.832251	83.5424	12.1606	0.50508	0.0718232	0.133544	0.130392
113	Frozen Olive	June	175	8718	0.139917	4.29011	2.28888	0.0929939	97.9812	-9.57375	34.4225	3.46928	135.46	42.0897	0.470324	1.06239	0.280206	0.0096664	0.142078	0.534847	0.0568443	0.232324	0.0507926	0.945407	0.557319	0.192755	5.27673	0.242584	21.8099	2.44307	3.30264	2.34061	0.670002	0.832251	83.5424	12.1606	0.50508	0.0718232	0.133544	0.130392
114	Frozen Olive	June	250	8718	0.215601	3.62764	2.29023	0.0943718	99.53	-7.82	23.3175	3.8999	157.798	34.3685	0.334454	1.14927	0.403573	0.106869	0.122066	0.535951	0.188134	0.353572	0.069779	1.46732	0.243412	0.348001	3.82189	1.69904	25.4052	3.40439	2.26635	1.7729	0.718413	0.672393	79.4515	11.308	0.776721	0.063435	0.206973	0.118586
115	Frozen Olive	June	250	8718	0.209133	3.51881	2.22152	0.0915407	96.5441	-7.5854	22.618	3.7829	153.064	34.3685	0.334454	1.14927	0.403573	0.106869	0.122066	0.535951	0.188134	0.353572	0.069779	1.46732	0.243412	0.348001	3.82189	1.69904	25.4052	3.40439	2.26635	1.7729	0.718413	0.672393	79.4515	11.308	0.776721	0.063435	0.206973	0.118586

116	Frozen Olive	June	250	8718	0.223147	3.7546	2.37039	0.0976748	103.014	-8.0937	24.1336	4.0364	163.321	34.3685	0.334454	1.14927	0.403573	0.106869	0.122066	0.535951	0.188134	0.353572	0.069779	1.46732	0.243412	0.348001	3.82189	1.69904	25.4052	3.40439	2.26635	1.7729	0.718413	0.672393	79.4515	11.308	0.776721	0.063435	0.206973	0.118586
117	Frozen Olive	June	250	8718	0.216453	3.64196	2.29927	0.0947446	99.9231	-7.85089	23.4096	3.9153	158.421	34.3685	0.334454	1.14927	0.403573	0.106869	0.122066	0.535951	0.188134	0.353572	0.069779	1.46732	0.243412	0.348001	3.82189	1.69904	25.4052	3.40439	2.26635	1.7729	0.718413	0.672393	79.4515	11.308	0.776721	0.063435	0.206973	0.118586
118	Frozen Olive	July	100	5202	0.216247	4.19584	2.00377	0.0773849	100.573	-9.42	31.87	17.5416	126.064	49.002	0.436592	1.26304	0.468925	0.0328704	0.117314	0.545245	0.0802997	0.280027	0.0416262	0.0872468	1.31019	0.12488	15.9721	0.550135	23.2559	2.14378	0.644316	4.23346	1.24227	0.947231	102.779	18.9723	0.557065	0.053194	0.110789	0.0343473
119	Frozen Olive	July	100	5202	0.209759	4.06996	1.94366	0.0750634	97.5553	-9.1374	30.9139	17.0153	122.282	49.002	0.436592	1.26304	0.468925	0.0328704	0.117314	0.545245	0.0802997	0.280027	0.0416262	0.0872468	1.31019	0.12488	15.9721	0.550135	23.2559	2.14378	0.644316	4.23346	1.24227	0.947231	102.779	18.9723	0.557065	0.053194	0.110789	0.0343473
120	Frozen Olive	July	100	5202	0.223815	4.34269	2.07391	0.0800934	104.093	-9.7497	32.9855	18.1555	130.476	49.002	0.436592	1.26304	0.468925	0.0328704	0.117314	0.545245	0.0802997	0.280027	0.0416262	0.0872468	1.31019	0.12488	15.9721	0.550135	23.2559	2.14378	0.644316	4.23346	1.24227	0.947231	102.779	18.9723	0.557065	0.053194	0.110789	0.0343473
121	Frozen Olive	July	100	5202	0.217101	4.21241	2.01169	0.0776906	100.97	-9.45721	31.9959	17.6109	126.562	49.002	0.436592	1.26304	0.468925	0.0328704	0.117314	0.545245	0.0802997	0.280027	0.0416262	0.0872468	1.31019	0.12488	15.9721	0.550135	23.2559	2.14378	0.644316	4.23346	1.24227	0.947231	102.779	18.9723	0.557065	0.053194	0.110789	0.0343473
122	Frozen Olive	July	175	5202	0.192639	3.18326	1.99831	0.0738131	96.745	-12.485	46.255		124.763	35.711	0.281353	1.19302	0.893588	0.0384937	0.120053	0.561303	0.142988	0.332806	0.122325	1.36429	0.201167	0.407892	5.73781	0.431132	24.2668	3.23421	0.641005	2.20232	0.706675	0.718085	79.3083	11.3852	0.799587	0.0565507	0.138505	0.0439192
123	Frozen Olive	July	175	5202	0.18686	3.08776	1.93836	0.0715988	93.8427	-12.1104	44.8674		121.02	35.711	0.281353	1.19302	0.893588	0.0384937	0.120053	0.561303	0.142988	0.332806	0.122325	1.36429	0.201167	0.407892	5.73781	0.431132	24.2668	3.23421	0.641005	2.20232	0.706675	0.718085	79.3083	11.3852	0.799587	0.0565507	0.138505	0.0439192
124	Frozen Olive	July	175	5202	0.199381	3.29467	2.06825	0.0763966	100.131	-12.922	47.8739		129.13	35.711	0.281353	1.19302	0.893588	0.0384937	0.120053	0.561303	0.142988	0.332806	0.122325	1.36429	0.201167	0.407892	5.73781	0.431132	24.2668	3.23421	0.641005	2.20232	0.706675	0.718085	79.3083	11.3852	0.799587	0.0565507	0.138505	0.0439192
125	Frozen Olive	July	175	5202	0.1934	3.19583	2.00621	0.0741047	97.1271	-12.5343	46.4377		125.256	35.711	0.281353	1.19302	0.893588	0.0384937	0.120053	0.561303	0.142988	0.332806	0.122325	1.36429	0.201167	0.407892	5.73781	0.431132	24.2668	3.23421	0.641005	2.20232	0.706675	0.718085	79.3083	11.3852	0.799587	0.0565507	0.138505	0.0439192
126	Frozen Olive	July	175	5202	0.192639	3.18326	1.99831	0.0738131	96.745	-12.485	46.255	3.50038	124.15	62.3749	0.666907	1.44119	0.897273	0.266319	0.423593	0.945447	0.251831	0.328955	0.0602015	3.5483	1.17132	0.317623	12.1672	0.927998	24.5352	3.25758	1.57115	4.19275	1.24084	1.01752	121.604	20.8618	0.451032	0.0530873	0.204878	0.0440365
127	Frozen Olive	July	175	5202	0.18686	3.08776	1.93836	0.0715987	93.8427	-12.1104	44.8674	3.39537	120.425	62.3749	0.666907	1.44119	0.897273	0.266319	0.423593	0.945447	0.251831	0.328955	0.0602015	3.5483	1.17132	0.317623	12.1672	0.927998	24.5352	3.25758	1.57115	4.19275	1.24084	1.01752	121.604	20.8618	0.451032	0.0530873	0.204878	0.0440365
128	Frozen Olive	July	175	5202	0.199381	3.29467	2.06825	0.0763966	100.131	-12.922	47.8739	3.62289	128.495	62.3749	0.666907	1.44119	0.897273	0.266319	0.423593	0.945447	0.251831	0.328955	0.0602015	3.5483	1.17132	0.317623	12.1672	0.927998	24.5352	3.25758	1.57115	4.19275	1.24084	1.01752	121.604	20.8618	0.451032	0.0530873	0.204878	0.0440365
129	Frozen Olive	July	175	5202	0.1934	3.19583	2.0062	0.0741047	97.1271	-12.5343	46.4377	3.51421	124.64	62.3749	0.666907	1.44119	0.897273	0.266319	0.423593	0.945447	0.251831	0.328955	0.0602015	3.5483	1.17132	0.317623	12.1672	0.927998	24.5352	3.25758	1.57115	4.19275	1.24084	1.01752	121.604	20.8618	0.451032	0.0530873	0.204878	0.0440365
130	Frozen Olive	July	250	5202	0.23133	5.07395	2.13843	0.0842849	101.76	-9.455	32.39	3.95804	105.953																											
131	Frozen Olive	July	250	5202	0.201164	3.31773	1.86912	0.070485	99.385	-9.385	31.35	4.54798	145.652																											
132	Frozen Olive	July	250	5202	0.216247	4.19584	2.00377	0.0773849	100.573	-9.42	31.87	4.25301	125.802																											
133	Frozen Olive	July	250	5202	0.208705	3.75678	1.93645	0.073935	99.9788	-9.4025	31.61	4.4005	135.727																											
134	Frozen Olive	July	0	6959	0.203937	2.35534	2.42909	0.084793	102.26	-5.26	15.52	6.0684	96.7376	64.0859	0.448848	1.07076	0.234304	0.0355799	0.109358	0.708135	0.0379433	0.2415	0.0407085	1.57792	0.702744	0.112622	15.8441	0.301069	19.8613	1.46134	0.774037	4.68521	1.07766	1.04892	114.46	19.77	0.577017	0.0472158	0.0577991	0.0213699
135	Frozen Olive	July	0	6959	0.200701	2.4158	2.15162	0.0889065	102.18	-5.24	16.11		102.863	64.0859	0.448848	1.07076	0.234304	0.0355799	0.109358	0.708135	0.0379433	0.2415	0.0407085	1.57792	0.702744	0.112622	15.8441	0.301069	19.8613	1.46134	0.774037	4.68521	1.07766	1.04892	114.46	19.77	0.577017	0.0472158	0.0577991	0.0213699
136	Frozen Olive	July	0	6959	0.218293	2.49641	1.72059	0.069712	103.06	-5.34	14.08	6.81726	131.823	64.0859	0.448848	1.07076	0.234304	0.0355799	0.109358	0.708135	0.0379433	0.2415	0.0407085	1.57792	0.702744	0.112622	15.8441	0.301069	19.8613	1.46134	0.774037	4.68521	1.07766	1.04892	114.46	19.77	0.577017	0.0472158	0.0577991	0.0213699
137	Frozen Olive	July	0	6959	0.203898	1.69718	1.6537	0.0714286	103.74	-5.26	14.54		132.711	64.0859	0.448848	1.07076	0.234304	0.0355799	0.109358	0.708135	0.0379433	0.2415	0.0407085	1.57792	0.702744	0.112622	15.8441	0.301069	19.8613	1.46134	0.774037	4.68521	1.07766	1.04892	114.46	19.77	0.577017	0.0472158	0.0577991	0.0213699
138	Frozen Olive	July	100	6959	0.174446	3.25375	2.21827	0.0749239	98.06	-10.35	37.07	3.62717	116.138	139.812	0.391135	1.0852	0.497083	0.392688	0.295554	0.558458	0.173188	0.268346	0.0216885	0.409108	0.574085	0.156839	7.38001	0.245498	23.5385	0.740327	1.25191	5.65011	1.53015	1.27768	186.25	9.9382	0.546997	0.0537369	0.185792	0.0435219
139	Frozen Olive	July	100	6959	0.159927	3.02733	1.98028	0.083727	98.725	-10.065	38.245	2.83123	119.871	139.812	0.391135	1.0852	0.497083	0.392688	0.295554	0.558458	0.173188	0.268346	0.0216885	0.409108	0.574085	0.156839	7.38001	0.245498	23.5385	0.740327	1.25191	5.65011	1.53015	1.27768	186.25	9.9382	0.546997	0.0537369	0.185792	0.0435219
140	Frozen Olive	July	100	6959	0.167186	3.14054	2.09928	0.0793255	98.3925	-10.2075	37.6575	3.2292	118.005	139.812	0.391135	1.0852	0.497083	0.392688	0.295554	0.558458	0.173188	0.268346	0.0216885	0.409108	0.574085	0.156839	7.38001	0.245498	23.5385	0.740327	1.25191	5.65011	1.53015	1.27768	186.25	9.9382	0.546997	0.0537369	0.185792	0.0435219
141	Frozen Olive	July	100	6959	0.163557	3.08394	2.03978	0.0815262	98.5587	-10.1362	37.9512	3.03022	118.938	139.812	0.391135	1.0852	0.497083	0.392688	0.295554	0.558458	0.173188	0.268346	0.0216885	0.409108	0.574085	0.156839	7.38001	0.245498	23.5385	0.740327	1.25191	5.65011	1.53015	1.27768	186.25	9.9382	0.546997	0.0537369	0.185792	0.0435219
142	Frozen Olive	July	100	6959	0.0706683	3.95146	2.13506	0.0766606	101.69	-8.655	28.51	2.89619	145.041	34.668	0.347707	1.16196	1.02055	0.167373	0.545056	0.567787	0.131089	0.30372	0.0319937	1.16303	0.956784	0.386243	4.91861	0.71198	24.2239	1.61078	0.913997	3.95655	1.49762	1.13603	80.4208	8.99266	0.68177	0.058972	0.158183	0.0959194
143	Frozen Olive	July	100	6959	0.0933632	3.51799	2.05123	0.0747693	104.58	-9.07	31.05	3.16455	138.619	34.668	0.347707	1.16196	1.02055	0.167373	0.545056	0.567787	0.131089	0.30372	0.0319937	1.16303	0.956784	0.386243	4.91861	0.71198	24.2239	1.61078	0.913997	3.95655	1.49762	1.13603	80.4208	8.99266	0.68177	0.058972	0.158183	0.0959194
144	Frozen Olive	July	100	6959	0.0820158	3.73472	2.09314	0.075715	103.135	-8.8625	29.78	3.03037	141.83	34.668	0.347707	1.16196	1.02055	0.167373	0.545056	0.567787	0.131089	0.30372	0.0319937	1.16303	0.956784	0.386243	4.91861	0.71198	24.2239	1.61078	0.913997	3.95655	1.49762	1.13603	80.4208	8.99266	0.68177	0.058972	0.158183	0.0959194
145	Frozen Olive	July	100	6959	0.0876895	3.62636	2.07219	0.0752421	103.857	-8.96625	30.415	3.09746	140.225	34.668	0.347707	1.16196	1.02055	0.167373	0.545056	0.567787	0.131089	0.30372	0.0319937	1.16303	0.956784	0.386243	4.91861	0.71198	24.2239	1.61078	0.913997	3.95655	1.49762	1.13603	80.4208	8.99266	0.68177	0.058972	0.158183	0.0959194
146	Frozen Olive	July	175	6959	0.104412	3	2	0.075	90.685	-8.475	39.95	3	155																											
147	Frozen Olive	July	175	6959	0.230539	3.02692	2.29776	0.0844309	101.75	-11.45	38.875	3.14738	157.23																											
148	Frozen Olive	July	175	6959	0.167476	3.01346	2.14888	0.0797155	96.2175	-9.9625	39.4125	3.07369	156.115	87.0262	0.422226	1.58607	0.947136	0.162491	0.475935	0.918989	0.189527	0.323217	0.0269669	2.07623	0.715839	0.345071	8.31627	0.288358	27.8349	3.25212	1.41984	2.0099	0.507792	0.699672	139.545	15.4095	0.410914	0.053647	0.234457	0.0462354
149	Frozen Olive	July	175	6959	0.199007	3.02019	2.22332	0.0820732	98.9837	-10.7063	39.1437	3.11053	156.673																											
150	Frozen Olive	July	175	6959	0.354007	3.72719	2.09678	0.0877493	99.825	-10.625	38.03	3.93608	153.761	57.2196	0.497858	1.2752	1.18989	0.0829004	0.201621	0.585468	0.277305	0.34902	0.0193233	0.760945	0.37184	0.314393	2.58691	2.56544	22.8183	3.24514	1.03132	3.77515	1.75641	1.13711	102.061	7.93871	0.470416	0.0587604	0.171962	0.0272407
151	Frozen Olive	July	175	6959	0.181265	4.22451	2.16407	0.0847	99.75	-12.13	43.77	2.6206	154.415	24.3264	0.291853	1.24777	1.65798	0.0298204	0.23263	0.581501	0.244599	0.351693	0.148639	1.59022	0.106632	0.583917	3.439	0.642163	24.1587	2.47171	0.744698	3.29132	1.48105	0.919323	68.5416	8.82955	0.404151	0.0582145	0.159322	0.0808376
152	Frozen Olive	July	175	6959	0.267636	3.97585	2.13042	0.0862247	99.7875	-11.3775	40.9	3.27834	154.088																											
153	Frozen Olive	July	175	6959	0.22445	4.10018	2.14725	0.0854623	99.7688	-11.7537	42.335	2.94947	154.251																											
154	Frozen Olive	July	250	6959	0.275246	2.32253	1.98167	0.0685641	101.625	-13.575	49.27	4.43835	163.534	58.4247	0.440205	1.41975	0.523075	0.109028	0.150468	0.815781	0.0991781	0.275318	0.106606	3.52495	0.196212	0.207606	14.3922	0.502272	25.1978	2.342	8.51512	3.99708	0.863241	0.908216	123.011	28.9819	0.842602	0.0493903	0.19361	0.034259
155	Frozen Olive	July	250	6959	0.235215	2.80568	2.05128	0.0660572	101.255	-11.64	41.695	3.47159	154.33	58.4247	0.440205	1.41975	0.523075	0.109028	0.150468	0.815781	0.0991781	0.275318	0.106606	3.52495	0.196212	0.207606	14.3922	0.502272	25.1978	2.342	8.51512	3.99708	0.863241	0.908216	123.011	28.9819	0.842602	0.0493903	0.19361	0.034259
156	Frozen Olive	July	250	6959	0.25523	2.56411	2.01647	0.0673107	101.44	-12.6075	45.4825	3.95497	158.932	58.4247	0.440205	1.41975	0.523075	0.109028	0.150468	0.815781	0.0991781	0.275318	0.106606	3.52495	0.196212	0.207606	14.3922	0.502272	25.1978	2.342	8.51512	3.99708	0.863241	0.908216	123.011	28.9819	0.842602	0.0493903	0.19361	0.034259
157	Frozen Olive	July	250	6959	0.245223	2.68489	2.03388	0.0666839	101.348	-12.1237	43.5887	3.71328	156.631	58.4247	0.440205	1.41975	0.523075	0.109028	0.150468	0.815781	0.0991781	0.275318	0.106606	3.52495	0.196212	0.207606	14.3922	0.502272	25.1978	2.342	8.51512	3.99708	0.863241	0.908216	123.011	28.9819	0.842602	0.0493903	0.19361	0.034259
158	Frozen Olive	July	250	6959	0.193386	4.20034	2.35257	0.0894923	101.4	-5.9	20.195	3.24241	130.147	9.15832	0.288043	1.1198	1.40801	0.0443662	0.133107	0.650026	0.110232	0.413443	0.0261778	2.03728	0.126358	0.724654	0.810025	0.0824283	24.8255	3.2869	5.62827	2.80782	0.943742	0.740247	55.3648	12.4871	1.32963	0.051867	0.11043	0.0316861
159	Frozen Olive	July	250	6959	0.209658	3.58627	2.35992	0.0891327	99.605	-6.335	21.755	2.31019	158.509	9.15832	0.288043	1.1198	1.40801	0.0443662	0.133107	0.650026	0.110232	0.413443	0.0261778	2.03728	0.126358	0.724654	0.810025	0.0824283	24.8255	3.2869	5.62827	2.80782	0.943742	0.740247	55.3648	12.4871	1.32963	0.051867	0.11043	0.0316861
160	Frozen Olive	July	250	6959	0.201522	3.89331	2.35625	0.0893125	100.503	-6.1175	20.975	2.7763	144.328	9.15832	0.288043	1.1198	1.40801	0.0443662	0.133107	0.650026	0.110232	0.413443	0.0261778	2.03728	0.126358	0.724654	0.810025	0.0824283	24.8255	3.2869	5.62827	2.80782	0.943742	0.740247	55.3648	12.4871	1.32963	0.051867	0.11043	0.0316861
161	Frozen Olive	July	250	6959	0.20559	3.73979	2.35808	0.0892226	100.054	-6.22625	21.365	2.54325	151.419	9.15832	0.288043	1.1198	1.40801	0.0443662	0.133107	0.650026	0.110232	0.413443	0.0261778	2.03728	0.126358	0.724654	0.810025	0.0824283	24.8255	3.2869	5.62827	2.80782	0.943742	0.740247	55.3648	12.4871	1.32963	0.051867	0.11043	0.0316861
162	Frozen Olive	July	100	8718										13.6356	0.399724	1.09754	1.13521	0.0116959	0.122797	0.528223	0.0790909	0.3673	0.0392652	1.18598	0.13406	0.300422	2.70259	0.623959	20.581	3.09645	3.13806	1.90135	0.542805	0.500361	52.1235	10.4235	1.07739	0.141985	0.0416701	0.0195105
163	Frozen Olive	July	175	8718	0.241972	2.7573	2.36635	0.0861558	103.86	-8.345	24.69	4.34037	176.177	13.6356	0.399724	1.09754	1.13521	0.0116959	0.122797	0.528223	0.0790909	0.3673	0.0392652	1.18598	0.13406	0.300422	2.70259	0.623959	20.581	3.09645	3.13806	1.90135	0.542805	0.500361	52.1235	10.4235	1.07739	0.141985	0.0416701	0.0195105
164	Frozen Olive	July	175	8718	0.106056	2.51822	2.42619	0.0815063	98.98	-12.125	40.37	3.55834	127.343	13.6356	0.399724	1.09754	1.13521	0.0116959	0.122797	0.528223	0.0790909	0.3673	0.0392652	1.18598	0.13406	0.300422	2.70259	0.623959	20.581	3.09645	3.13806	1.90135	0.542805	0.500361	52.1235	10.4235	1.07739	0.141985	0.0416701	0.0195105
165	Frozen Olive	July	175	8718	0.174014	2.63776	2.39627	0.083831	101.42	-10.235	32.53	3.94935	151.76	13.6356	0.399724	1.09754	1.13521	0.0116959	0.122797	0.528223	0.0790909	0.3673	0.0392652	1.18598	0.13406	0.300422	2.70259	0.623959	20.581	3.09645	3.13806	1.90135	0.542805	0.500361	52.1235	10.4235	1.07739	0.141985	0.0416701	0.0195105
166	Frozen Olive	July	175	8718	0.140035	2.57799	2.41123	0.0826687	100.2	-11.18	36.45	3.75385	139.551	13.6356	0.399724	1.09754	1.13521	0.0116959	0.122797	0.528223	0.0790909	0.3673	0.0392652	1.18598	0.13406	0.300422	2.70259	0.623959	20.581	3.09645	3.13806	1.90135	0.542805	0.500361	52.1235	10.4235	1.07739	0.141985	0.0416701	0.0195105
167	Frozen Olive	July	175	8718	0.0863064	2.99638	2.00019	0.0723773	101.99	-11.55	41.165	3.18973	148.196	81.328	0.581671	1.173	0.592395	0.0760505	0.117115	0.547263	0.0935545	0.286206	0.0252997	0.827314	0.735753	0.273152	9.07671	0.476832	22.6099	2.69782	0.328629	4.53321	1.68579	1.01042	129.076	13.2036	0.748599	0.0473161	0.0228153	0.0189675
168	Frozen Olive	July	175	8718	0.172269	3.43815	2.0843	0.0756066	103.405	-8.085	24.345	3.63157	145.048	81.328	0.581671	1.173	0.592395	0.0760505	0.117115	0.547263	0.0935545	0.286206	0.0252997	0.827314	0.735753	0.273152	9.07671	0.476832	22.6099	2.69782	0.328629	4.53321	1.68579	1.01042	129.076	13.2036	0.748599	0.0473161	0.0228153	0.0189675
169	Frozen Olive	July	175	8718	0.129288	3.21726	2.04224	0.0739919	102.698	-9.8175	32.755	3.41065	146.622	81.328	0.581671	1.173	0.592395	0.0760505	0.117115	0.547263	0.0935545	0.286206	0.0252997	0.827314	0.735753	0.273152	9.07671	0.476832	22.6099	2.69782	0.328629	4.53321	1.68579	1.01042	129.076	13.2036	0.748599	0.0473161	0.0228153	0.0189675
170	Frozen Olive	July	175	8718	0.150778	3.32771	2.06327	0.0747993	103.051	-8.95125	28.55	3.52111	145.835	81.328	0.581671	1.173	0.592395	0.0760505	0.117115	0.547263	0.0935545	0.286206	0.0252997	0.827314	0.735753	0.273152	9.07671	0.476832	22.6099	2.69782	0.328629	4.53321	1.68579	1.01042	129.076	13.2036	0.748599	0.0473161	0.0228153	0.0189675
171	Frozen Olive	July	250	8718	0.121291	2.37786	2.18	0.05	100.65	-8.25	25.48	4.31671	137	19.6537	0.459193	1.23129	1.05988	0.0921241	0.650129	0.61834	0.157444	0.296416	0.0419467	0.0903824	1.47016	0.863328	4.25516	0.519044	22.4652	3.54968	1.53692	1.21716	0.624875	0.522332	61.3747	10.2955	0.681439	0.049708	0.127684	0.0416197
172	Frozen Olive	July	250	8718	0.220355	2.787	2.24923	0.0690989	98.375	-12.7	45.24	3.50911	140.198	19.6537	0.459193	1.23129	1.05988	0.0921241	0.650129	0.61834	0.157444	0.296416	0.0419467	0.0903824	1.47016	0.863328	4.25516	0.519044	22.4652	3.54968	1.53692	1.21716	0.624875	0.522332	61.3747	10.2955	0.681439	0.049708	0.127684	0.0416197
173	Frozen Olive	July	250	8718	0.170823	2.58243	2.21462	0.0595495	99.5125	-10.475	35.36	3.91291	138.599	19.6537	0.459193	1.23129	1.05988	0.0921241	0.650129	0.61834	0.157444	0.296416	0.0419467	0.0903824	1.47016	0.863328	4.25516	0.519044	22.4652	3.54968	1.53692	1.21716	0.624875	0.522332	61.3747	10.2955	0.681439	0.049708	0.127684	0.0416197
174	Frozen Olive	July	250	8718	0.195589	2.68472	2.23192	0.0643242	98.9437	-11.5875	40.3	3.71101	139.398	19.6537	0.459193	1.23129	1.05988	0.0921241	0.650129	0.61834	0.157444	0.296416	0.0419467	0.0903824	1.47016	0.863328	4.25516	0.519044	22.4652	3.54968	1.53692	1.21716	0.624875	0.522332	61.3747	10.2955	0.681439	0.049708	0.127684	0.0416197
175	Fresh Olive	May	100	5202	0.128351	5.0463	2.3125	0.0924799	40.395	-2.675	18.62	4.29877	130.419	103.396	0.770865	1.91689	0.438232	0.0231105	0.212788	1.14262	0.298441	0.327999	0.19605	8.44081	0.251023	0.297009	36.4167	1.45752	26.721	3.34689	4.57914	3.02382	1.14603	0.937072	195.34	53.0806	0.424075	0.0988912	0.401454	0.203439
176	Fresh Olive	May	100	5202	0.106346	3.36747	2.08369	0.0829715	77.65	-5.53	34.935	5.14855	147.487	103.396	0.770865	1.91689	0.438232	0.0231105	0.212788	1.14262	0.298441	0.327999	0.19605	8.44081	0.251023	0.297009	36.4167	1.45752	26.721	3.34689	4.57914	3.02382	1.14603	0.937072	195.34	53.0806	0.424075	0.0988912	0.401454	0.203439
177	Fresh Olive	May	100	5202	0.117348	4.20688	2.1981	0.0877257	59.0225	-4.1025	26.7775	4.72366	138.953	103.396	0.770865	1.91689	0.438232	0.0231105	0.212788	1.14262	0.298441	0.327999	0.19605	8.44081	0.251023	0.297009	36.4167	1.45752	26.721	3.34689	4.57914	3.02382	1.14603	0.937072	195.34	53.0806	0.424075	0.0988912	0.401454	0.203439
178	Fresh Olive	May	100	5202	0.111847	3.78718	2.14089	0.0853486	68.3363	-4.81625	30.8563	4.9361	143.22	103.396	0.770865	1.91689	0.438232	0.0231105	0.212788	1.14262	0.298441	0.327999	0.19605	8.44081	0.251023	0.297009	36.4167	1.45752	26.721	3.34689	4.57914	3.02382	1.14603	0.937072	195.34	53.0806	0.424075	0.0988912	0.401454	0.203439
179	Fresh Olive	May	175	5202	0.0823873	5.65216	2.05528	0.0877953	48.52	-1.845	24.48	4.23126	155.631	96.0971	0.350489	1.66117	0.591182	0.016361	0.228603	1.25382	0.435758	0.293005	0.0204453	11.7427	0.121443	0.156069	27.2565	2.75994	28.5568	5.87392	4.07223	2.97258	1.27109	1.12632	186.857	49.1014	0.318567	0.113639	0.537194	0.307228
180	Fresh Olive	May	175	5202	0.12468	5.07868	2.02405	0.0906316	97.795	-10.82	49.37	5.20424	180.335	96.0971	0.350489	1.66117	0.591182	0.016361	0.228603	1.25382	0.435758	0.293005	0.0204453	11.7427	0.121443	0.156069	27.2565	2.75994	28.5568	5.87392	4.07223	2.97258	1.27109	1.12632	186.857	49.1014	0.318567	0.113639	0.537194	0.307228
181	Fresh Olive	May	175	5202	0.103534	5.36542	2.03966	0.0892135	73.1575	-6.3325	36.925	4.71775	167.983	96.0971	0.350489	1.66117	0.591182	0.016361	0.228603	1.25382	0.435758	0.293005	0.0204453	11.7427	0.121443	0.156069	27.2565	2.75994	28.5568	5.87392	4.07223	2.97258	1.27109	1.12632	186.857	49.1014	0.318567	0.113639	0.537194	0.307228
182	Fresh Olive	May	175	5202	0.114107	5.22205	2.03186	0.0899225	85.4762	-8.57625	43.1475	4.961	174.159	96.0971	0.350489	1.66117	0.591182	0.016361	0.228603	1.25382	0.435758	0.293005	0.0204453	11.7427	0.121443	0.156069	27.2565	2.75994	28.5568	5.87392	4.07223	2.97258	1.27109	1.12632	186.857	49.1014	0.318567	0.113639	0.537194	0.307228
183	Fresh Olive	May	175	5202	0.10882	5.29374	2.03576	0.089568	79.3169	-7.45437	40.0363	4.83937	171.071	96.0971	0.350489	1.66117	0.591182	0.016361	0.228603	1.25382	0.435758	0.293005	0.0204453	11.7427	0.121443	0.156069	27.2565	2.75994	28.5568	5.87392	4.07223	2.97258	1.27109	1.12632	186.857	49.1014	0.318567	0.113639	0.537194	0.307228
184	Fresh Olive	May	175	5202	0.153793	2.30767	1.97344	0.100798	42.275	-0.86	24.58	6.36497	137.347	265.528	2.22508	1.26909	0.529823	0.0566094	0.353061	2.29741	0.665233	0.26742	0.123656	158.282	0.463772	1.89782	48.7638	2.4455	29.9624	7.09603	14.0773	5.82584	1.55257	1.41214	545.095	230.117	0.342404	0.116652	0.62241	0.170813
185	Fresh Olive	May	175	5202	0.170879	2.8077	1.95892	0.093801	79.38	-4.55	33.945	4.36326	122.795	265.528	2.22508	1.26909	0.529823	0.0566094	0.353061	2.29741	0.665233	0.26742	0.123656	158.282	0.463772	1.89782	48.7638	2.4455	29.9624	7.09603	14.0773	5.82584	1.55257	1.41214	545.095	230.117	0.342404	0.116652	0.62241	0.170813
186	Fresh Olive	May	175	5202	0.162336	2.55768	1.96618	0.0972995	60.8275	-2.705	29.2625	5.36412	130.071	265.528	2.22508	1.26909	0.529823	0.0566094	0.353061	2.29741	0.665233	0.26742	0.123656	158.282	0.463772	1.89782	48.7638	2.4455	29.9624	7.09603	14.0773	5.82584	1.55257	1.41214	545.095	230.117	0.342404	0.116652	0.62241	0.170813
187	Fresh Olive	May	175	5202	0.166607	2.68269	1.96255	0.0955502	70.1038	-3.6275	31.6038	4.86369	126.433	265.528	2.22508	1.26909	0.529823	0.0566094	0.353061	2.29741	0.665233	0.26742	0.123656	158.282	0.463772	1.89782	48.7638	2.4455	29.9624	7.09603	14.0773	5.82584	1.55257	1.41214	545.095	230.117	0.342404	0.116652	0.62241	0.170813
188	Fresh Olive	May	250	5202	0.137115	4.57553	2.23828	0.0958162	53.275	-2.035	25.865	5.4873	135.618	46.9501	0.323424	2.07823	0.95807	0.00468875	0.125835	0.728799	0.307755	0.324971	0.898544	2.79425	0.234607	0.399888	12.6763	0.446332	26.527	4.69297	4.27863	2.84842	0.670414	0.906482	109.176	24.842	0.463069	0.0791334	0.35685	0.159598
189	Fresh Olive	May	250	5202	0.0998672	5.08751	2.22934	0.0895214	58.965	-1.78	25.26	5.3029	150.13	46.9501	0.323424	2.07823	0.95807	0.00468875	0.125835	0.728799	0.307755	0.324971	0.898544	2.79425	0.234607	0.399888	12.6763	0.446332	26.527	4.69297	4.27863	2.84842	0.670414	0.906482	109.176	24.842	0.463069	0.0791334	0.35685	0.159598
190	Fresh Olive	May	250	5202	0.118491	4.83152	2.23381	0.0926688	56.12	-1.9075	25.5625	5.3951	142.874	46.9501	0.323424	2.07823	0.95807	0.00468875	0.125835	0.728799	0.307755	0.324971	0.898544	2.79425	0.234607	0.399888	12.6763	0.446332	26.527	4.69297	4.27863	2.84842	0.670414	0.906482	109.176	24.842	0.463069	0.0791334	0.35685	0.159598
191	Fresh Olive	May	250	5202	0.109179	4.95952	2.23158	0.0910951	57.5425	-1.84375	25.4112	5.349	146.502	46.9501	0.323424	2.07823	0.95807	0.00468875	0.125835	0.728799	0.307755	0.324971	0.898544	2.79425	0.234607	0.399888	12.6763	0.446332	26.527	4.69297	4.27863	2.84842	0.670414	0.906482	109.176	24.842	0.463069	0.0791334	0.35685	0.159598
192	Fresh Olive	May	0	6959	0.172981	3.63949	2.01792	0.0980198	92.33	-7.63	32.67	5.33431	157.76	71.4066	0.929967	1.73543	0.211692	0.0176147	0.130194	0.796045	0.23215	0.216102	0.163819	20.2998	2.18408	0.213037	55.8672	2.95616	26.6738	4.06149	17.6052	4.22941	1.65694	1.69074	213.278	98.0468	0.292778	0.109138	0.603503	0.196839
193	Fresh Olive	May	0	6959	0.139992	3.61162	1.97611	0.0957741	91.78	-7.71	31.66		155.209	71.4066	0.929967	1.73543	0.211692	0.0176147	0.130194	0.796045	0.23215	0.216102	0.163819	20.2998	2.18408	0.213037	55.8672	2.95616	26.6738	4.06149	17.6052	4.22941	1.65694	1.69074	213.278	98.0468	0.292778	0.109138	0.603503	0.196839
194	Fresh Olive	May	0	6959	0.117469	2.35491	1.98901	0.0913537	99.44	-11.68	40.29	4.93113	175.222	71.4066	0.929967	1.73543	0.211692	0.0176147	0.130194	0.796045	0.23215	0.216102	0.163819	20.2998	2.18408	0.213037	55.8672	2.95616	26.6738	4.06149	17.6052	4.22941	1.65694	1.69074	213.278	98.0468	0.292778	0.109138	0.603503	0.196839
195	Fresh Olive	May	0	6959	0.0838368	2.73273	2.07317	0.0900757	95.39	-11.15	39.29		173.344	71.4066	0.929967	1.73543	0.211692	0.0176147	0.130194	0.796045	0.23215	0.216102	0.163819	20.2998	2.18408	0.213037	55.8672	2.95616	26.6738	4.06149	17.6052	4.22941	1.65694	1.69074	213.278	98.0468	0.292778	0.109138	0.603503	0.196839
196	Fresh Olive	May	100	6959	0.109943	4.81137	2.16179	0.0896863	21.425	-2.08	13.51	3.84072	121.383	178.06	0.29225	2.22092	0.414122	0.0116936	0.0989223	0.573683	0.215647	0.262292	0.110826	0.571851	0.284754	0.250779	24.5765	0.297128	26.7874	3.44319	5.14199	5.20448	1.25404	1.23609	251.309	33.9843	1.14884	0.107031	0.953445	0.175789
197	Fresh Olive	May	100	6959	0.0975848	2.64605	2.02889	0.0922158	49.745	-2.615	25.455	4.18288	132.975	178.06	0.29225	2.22092	0.414122	0.0116936	0.0989223	0.573683	0.215647	0.262292	0.110826	0.571851	0.284754	0.250779	24.5765	0.297128	26.7874	3.44319	5.14199	5.20448	1.25404	1.23609	251.309	33.9843	1.14884	0.107031	0.953445	0.175789
198	Fresh Olive	May	100	6959	0.103764	3.72871	2.09534	0.090951	35.585	-2.3475	19.4825	4.0118	127.179	178.06	0.29225	2.22092	0.414122	0.0116936	0.0989223	0.573683	0.215647	0.262292	0.110826	0.571851	0.284754	0.250779	24.5765	0.297128	26.7874	3.44319	5.14199	5.20448	1.25404	1.23609	251.309	33.9843	1.14884	0.107031	0.953445	0.175789
199	Fresh Olive	May	100	6959	0.100674	3.18738	2.06211	0.0915834	42.665	-2.48125	22.4688	4.09734	130.077	178.06	0.29225	2.22092	0.414122	0.0116936	0.0989223	0.573683	0.215647	0.262292	0.110826	0.571851	0.284754	0.250779	24.5765	0.297128	26.7874	3.44319	5.14199	5.20448	1.25404	1.23609	251.309	33.9843	1.14884	0.107031	0.953445	0.175789
200	Fresh Olive	May	100	6959	0.0923869	2.62634	1.94303	0.0849882	93.825	-11.12	46.9	4.25051	182.535	100.542	0.384027	1.71067	0.337888	0.0189057	0.114358	0.65199	0.0886024	0.216145	0.05761	1.78486	0.442117	0.250351	9.35558	1.51705	25.6975	4.52489	4.40944	1.45783	1.06447	0.844803	155.471	20.3251	0.511872	0.105018	0.547729	0.27539
201	Fresh Olive	May	100	6959	0.153879	2.58956	1.99877	0.0834423	64.735	-2.975	29.22	4.09573	204.608	100.542	0.384027	1.71067	0.337888	0.0189057	0.114358	0.65199	0.0886024	0.216145	0.05761	1.78486	0.442117	0.250351	9.35558	1.51705	25.6975	4.52489	4.40944	1.45783	1.06447	0.844803	155.471	20.3251	0.511872	0.105018	0.547729	0.27539
202	Fresh Olive	May	100	6959	0.123133	2.60795	1.9709	0.0842153	79.28	-7.0475	38.06	4.17312	193.572	100.542	0.384027	1.71067	0.337888	0.0189057	0.114358	0.65199	0.0886024	0.216145	0.05761	1.78486	0.442117	0.250351	9.35558	1.51705	25.6975	4.52489	4.40944	1.45783	1.06447	0.844803	155.471	20.3251	0.511872	0.105018	0.547729	0.27539
203	Fresh Olive	May	100	6959	0.138506	2.59875	1.98484	0.0838288	72.0075	-5.01125	33.64	4.13443	199.09	100.542	0.384027	1.71067	0.337888	0.0189057	0.114358	0.65199	0.0886024	0.216145	0.05761	1.78486	0.442117	0.250351	9.35558	1.51705	25.6975	4.52489	4.40944	1.45783	1.06447	0.844803	155.471	20.3251	0.511872	0.105018	0.547729	0.27539
204	Fresh Olive	May	175	6959	0.261129	4.0557	2.02884	0.0919278	69.25	-3.845	35.915	5.63142	205.76	33.8063	0.479154	1.92076	1.48818	0.0180792	0.135032	1.27722	0.27434	0.405117	0.0895138	3.64537	0.261187	0.235464	5.43083	1.27785	24.8627	5.56221	2.729	1.33479	0.554294	0.656762	86.4442	17.6029	0.58053	0.112791	1.85485	0.336637
205	Fresh Olive	May	175	6959	0.0923377	2.64732	1.98359	0.0888213	77.785	-6.09	33.635	6.04849	174.494	51.4392	0.759351	1.3584	0.597062	0.0188944	0.12445	2.02584	0.429477	0.332197	0.0347549	33.2086	0.951335	0.236094	26.4794	2.84461	26.4387	5.17598	5.95886	4.85096	1.70969	1.67577	166.65	71.059	0.491684	0.0937521	0.48418	0.215553
206	Fresh Olive	May	175	6959	0.103196	5.47175	2.17326	0.0969885	28.66	-2.165	17.205	2.89517	193.403	60.1732	0.322524	3.6363	0.670731	0.020621	0.118979	0.533382	0.160562	0.303036	0.0443888	0.554819	0.14856	0.303733	9.45393	0.83896	30.1502	3.57359	1.99344	1.90625	0.471843	0.974916	116.354	15.8795	1.18447	0.144902	1.33462	0.188013
207	Fresh Olive	May	175	6959	0.105869	5.77675	2.07206	0.0907359	25.095	-2.415	17.135	3.20393	170.696	63.2229	0.414827	2.76893	0.654769	0.0115771	0.0788726	0.533445	0.0876791	0.259384	0.0227295	0.833363	0.108378	0.481469	7.54341	1.53847	30.5644	4.33161	2.49238	1.62419	0.57786	0.983349	119.134	15.6822	1.21521	0.165037	1.87197	0.160316
208	Fresh Olive	May	175	6959	0.127183	3.62382	1.86769	0.0809703	92.525	-8.24	34.26	2.68817	213.631	34.938	0.468056	1.69801	0.990915	0.00868975	0.132399	1.01398	0.332505	0.38071	0.151184	3.87235	0.0795052	0.348739	5.37797	0.151751	19.9732	2.86231	1.12634	2.1253	0.658335	0.695136	77.3854	13.5877	0.316738	0.0989947	0.64695	0.224247
209	Fresh Olive	May	175	6959	0.123338	4.56886	2.00481	0.084967	73.97	-3.745	29.98	2.69114	132.024	70.4362	0.358785	1.81907	0.65929	0.0171589	0.216532	1.39014	0.366499	0.322177	0.0447645	9.7356	0.266753	0.528935	17.1701	2.89046	29.2939	5.57631	3.40964	3.73369	1.06338	1.23262	150.532	36.4206	0.271475	0.104824	0.575188	0.207411
210	Fresh Olive	May	250	6959	0.120103	2.22876	1.8562	0.0861751	78.41	-4.82	36.025	5.0993	198.877	82.0156	0.791338	1.58737	0.448848	0.0314387	0.129122	3.3378	0.448763	0.290166	0.100298	53.8455	1.06261	0.161682	30.9197	1.24994	26.289	4.54856	15.1018	3.54211	1.13355	1.08236	228.117	104.577	0.287475	0.100553	0.638448	0.196568
211	Fresh Olive	May	250	6959	0.117598	3.32288	2.04841	0.100105	18.445	-2.42	14.9	3.65363	191.123	82.0156	0.791338	1.58737	0.448848	0.0314387	0.129122	3.3378	0.448763	0.290166	0.100298	53.8455	1.06261	0.161682	30.9197	1.24994	26.289	4.54856	15.1018	3.54211	1.13355	1.08236	228.117	104.577	0.287475	0.100553	0.638448	0.196568
212	Fresh Olive	May	250	6959	0.11885	2.77582	1.9523	0.0931401	48.4275	-3.62	25.4625	4.37646	195	82.0156	0.791338	1.58737	0.448848	0.0314387	0.129122	3.3378	0.448763	0.290166	0.100298	53.8455	1.06261	0.161682	30.9197	1.24994	26.289	4.54856	15.1018	3.54211	1.13355	1.08236	228.117	104.577	0.287475	0.100553	0.638448	0.196568
213	Fresh Olive	May	250	6959	0.118224	3.04935	2.00036	0.0966225	33.4362	-3.02	20.1812	4.01505	193.061	82.0156	0.791338	1.58737	0.448848	0.0314387	0.129122	3.3378	0.448763	0.290166	0.100298	53.8455	1.06261	0.161682	30.9197	1.24994	26.289	4.54856	15.1018	3.54211	1.13355	1.08236	228.117	104.577	0.287475	0.100553	0.638448	0.196568
214	Fresh Olive	May	250	6959	0.145499	2.90928	2.24385	0.10599	57.54	-2.785	27.095	3.56941	205.035	59.4156	0.624271	1.63664	0.5591	0.0060403	0.134665	0.99523	0.327208	0.31395	0.109009	9.88776	0.187238	0.300606	14.2399	1.18797	25.8223	7.50075	2.68833	2.10803	0.817139	0.82811	129.69	34.6173	0.315512	0.0989071	0.681984	0.219324
215	Fresh Olive	May	250	6959	0.0991519	2.12744	2.08522	0.0820006	96.445	-7.815	28.785	3.06385	191.225	59.4156	0.624271	1.63664	0.5591	0.0060403	0.134665	0.99523	0.327208	0.31395	0.109009	9.88776	0.187238	0.300606	14.2399	1.18797	25.8223	7.50075	2.68833	2.10803	0.817139	0.82811	129.69	34.6173	0.315512	0.0989071	0.681984	0.219324
216	Fresh Olive	May	250	6959	0.122325	2.51836	2.16454	0.0939953	76.9925	-5.3	27.94	3.31663	198.13	59.4156	0.624271	1.63664	0.5591	0.0060403	0.134665	0.99523	0.327208	0.31395	0.109009	9.88776	0.187238	0.300606	14.2399	1.18797	25.8223	7.50075	2.68833	2.10803	0.817139	0.82811	129.69	34.6173	0.315512	0.0989071	0.681984	0.219324
217	Fresh Olive	May	250	6959	0.110739	2.3229	2.12488	0.087998	86.7188	-6.5575	28.3625	3.19024	194.678	59.4156	0.624271	1.63664	0.5591	0.0060403	0.134665	0.99523	0.327208	0.31395	0.109009	9.88776	0.187238	0.300606	14.2399	1.18797	25.8223	7.50075	2.68833	2.10803	0.817139	0.82811	129.69	34.6173	0.315512	0.0989071	0.681984	0.219324
218	Fresh Olive	May	100	8718	0.132989	2.00746	2.00117	0.0993477	91.375	-9.815	43.63	6.11508	150.669	123.572	0.390021	1.43921	0.225799	0.00928612	0.13211	2.13593	0.366186	0.221586	0.0611019	76.9996	3.81243	0.114115	43.9761	7.68685	27.1341	2.58901	14.5224	5.8907	2.16311	1.44148	314.883	138.201	0.368926	0.108627	0.410445	0.227267
219	Fresh Olive	May	100	8718	0.148958	3.79643	2.10591	0.0928369	58.255	-2.505	29.08	4.32861	152.447	123.572	0.390021	1.43921	0.225799	0.00928612	0.13211	2.13593	0.366186	0.221586	0.0611019	76.9996	3.81243	0.114115	43.9761	7.68685	27.1341	2.58901	14.5224	5.8907	2.16311	1.44148	314.883	138.201	0.368926	0.108627	0.410445	0.227267
220	Fresh Olive	May	100	8718	0.140973	2.90195	2.05354	0.0960923	74.815	-6.16	36.355	5.22185	151.558	123.572	0.390021	1.43921	0.225799	0.00928612	0.13211	2.13593	0.366186	0.221586	0.0611019	76.9996	3.81243	0.114115	43.9761	7.68685	27.1341	2.58901	14.5224	5.8907	2.16311	1.44148	314.883	138.201	0.368926	0.108627	0.410445	0.227267
221	Fresh Olive	May	100	8718	0.144966	3.34919	2.07973	0.0944646	66.535	-4.3325	32.7175	4.77523	152.003	123.572	0.390021	1.43921	0.225799	0.00928612	0.13211	2.13593	0.366186	0.221586	0.0611019	76.9996	3.81243	0.114115	43.9761	7.68685	27.1341	2.58901	14.5224	5.8907	2.16311	1.44148	314.883	138.201	0.368926	0.108627	0.410445	0.227267
222	Fresh Olive	May	175	8718	0.0769813	4.27668	2.17742	0.0939661	75.95	-6.12	41.07	4.35667	179.374	28.4427	0.384618	1.40614	0.819717	0.00296231	0.137162	0.942069	0.216331	0.318261	0.0729331	2.24839	0.125134	0.317239	4.10308	1.11164	23.708	4.85475	0.537881	1.57623	0.572712	0.725769	72.6237	12.0613	0.307426	0.0962402	0.607206	0.208794
223	Fresh Olive	May	175	8718	0.0933038	3.88686	2.09828	0.0907321	80.24	-6.715	25.775	4.78396	185.164	28.4427	0.384618	1.40614	0.819717	0.00296231	0.137162	0.942069	0.216331	0.318261	0.0729331	2.24839	0.125134	0.317239	4.10308	1.11164	23.708	4.85475	0.537881	1.57623	0.572712	0.725769	72.6237	12.0613	0.307426	0.0962402	0.607206	0.208794
224	Fresh Olive	May	175	8718	0.0851426	4.08177	2.13785	0.0923491	78.095	-6.4175	33.4225	4.57031	182.269	28.4427	0.384618	1.40614	0.819717	0.00296231	0.137162	0.942069	0.216331	0.318261	0.0729331	2.24839	0.125134	0.317239	4.10308	1.11164	23.708	4.85475	0.537881	1.57623	0.572712	0.725769	72.6237	12.0613	0.307426	0.0962402	0.607206	0.208794
225	Fresh Olive	May	175	8718	0.0892232	3.98431	2.11807	0.0915406	79.1675	-6.56625	29.5987	4.67714	183.717	28.4427	0.384618	1.40614	0.819717	0.00296231	0.137162	0.942069	0.216331	0.318261	0.0729331	2.24839	0.125134	0.317239	4.10308	1.11164	23.708	4.85475	0.537881	1.57623	0.572712	0.725769	72.6237	12.0613	0.307426	0.0962402	0.607206	0.208794
226	Fresh Olive	May	175	8718	0.0938941	3.65822	1.69159	0.0875723	97.765	-10.435	41.515	4.00769	201.482	64.941	0.462669	1.33025	0.492536	0.0109252	0.122213	0.777667	0.0996768	0.252272	0.0584123	3.09544	0.0956911	0.161233	7.48226	0.278464	23.4397	3.2089	0.656051	1.07744	0.747266	0.726537	109.517	14.6039	0.298867	0.110983	0.725479	0.238925
227	Fresh Olive	May	175	8718	0.114557	3.03728	2.07771	0.0871238	92.47	-9.86	43.595	4.12659	166.859	64.941	0.462669	1.33025	0.492536	0.0109252	0.122213	0.777667	0.0996768	0.252272	0.0584123	3.09544	0.0956911	0.161233	7.48226	0.278464	23.4397	3.2089	0.656051	1.07744	0.747266	0.726537	109.517	14.6039	0.298867	0.110983	0.725479	0.238925
228	Fresh Olive	May	175	8718	0.104226	3.34775	1.88465	0.0873481	95.1175	-10.1475	42.555	4.06714	184.171	64.941	0.462669	1.33025	0.492536	0.0109252	0.122213	0.777667	0.0996768	0.252272	0.0584123	3.09544	0.0956911	0.161233	7.48226	0.278464	23.4397	3.2089	0.656051	1.07744	0.747266	0.726537	109.517	14.6039	0.298867	0.110983	0.725479	0.238925
229	Fresh Olive	May	175	8718	0.109391	3.19251	1.98118	0.0872359	93.7937	-10.0037	43.075	4.09687	175.515	64.941	0.462669	1.33025	0.492536	0.0109252	0.122213	0.777667	0.0996768	0.252272	0.0584123	3.09544	0.0956911	0.161233	7.48226	0.278464	23.4397	3.2089	0.656051	1.07744	0.747266	0.726537	109.517	14.6039	0.298867	0.110983	0.725479	0.238925
230	Fresh Olive	May	250	8718	0.139368	5.0011	2.24524	0.0972092	53.775	-2.555	25.68	4.73894	153.497	88.8236	0.708918	2.36864	0.724087	0.0171948	0.105248	0.896017	0.365081	0.39598	0.175453	8.11254	0.0673503	0.260396	12.0048	0.209	28.8521	4.99303	4.17126	2.64751	0.697269	0.94769	157.543	29.542	0.454208	0.103536	1.13221	0.222638
231	Fresh Olive	May	250	8718	0.135187	4.85107	2.17788	0.0942929	52.1618	-2.47835	24.9096	4.59677	148.892	86.1589	0.68765	2.29758	0.702364	0.016679	0.10209	0.869136	0.354129	0.3841	0.17019	7.86916	0.0653298	0.252584	11.6447	0.20273	27.9866	4.84324	4.04612	2.56808	0.676351	0.919259	152.817	28.6558	0.440582	0.10043	1.09825	0.215959

232	Fresh Olive	May	250	8718	0.144246	5.17614	2.32382	0.100612	55.6571	-2.64442	26.5788	4.9048	158.869	91.9324	0.73373	2.45154	0.74943	0.0177967	0.108931	0.927377	0.377859	0.409839	0.181594	8.39647	0.0697076	0.26951	12.425	0.216315	29.862	5.16779	4.31726	2.74017	0.721673	0.980859	163.057	30.576	0.470105	0.107159	1.17184	0.23043
233	Fresh Olive	June	250	8718	0.139919	5.02085	2.25411	0.0975932	53.9874	-2.56509	25.7814	4.75766	154.103	37.1844	0.711718	2.37799	0.726947	0.0172628	0.105663	0.899556	0.366523	0.397544	0.176146	8.14458	0.0676163	0.261424	12.0522	0.209825	28.9661	5.01275	4.18774	2.65797	0.700023	0.951433	158.165	29.6587	0.456002	0.103945	1.13668	0.223517
234	Fresh Olive	June	250	8718	0.135721	4.87023	2.18649	0.0946654	52.3678	-2.48814	25.008	4.61493	149.48	36.0688	0.690367	2.30665	0.705138	0.0167449	0.102493	0.872569	0.355528	0.385617	0.170862	7.90024	0.0655878	0.253582	11.6907	0.203531	28.0971	4.86237	4.06211	2.57823	0.679022	0.92289	153.421	28.769	0.442322	0.100826	1.10258	0.216812
235	Fresh Olive	June	250	8718	0.144816	5.19658	2.333	0.101009	55.877	-2.65487	26.6838	4.92418	159.497	38.4858	0.736628	2.46122	0.75239	0.017867	0.109362	0.93104	0.379352	0.411458	0.182311	8.42964	0.0699829	0.270574	12.474	0.217169	29.9799	5.1882	4.33431	2.75099	0.724524	0.984733	163.701	30.6968	0.471962	0.107583	1.17647	0.23134
236	Fresh Olive	June	250	8718	0.140471	5.04069	2.26301	0.0979787	54.2007	-2.57522	25.8833	4.77645	154.712	37.3312	0.71453	2.38739	0.729818	0.017331	0.106081	0.903109	0.367971	0.399114	0.176842	8.17675	0.0678834	0.262457	12.0998	0.210654	29.0805	5.03255	4.20428	2.66846	0.702788	0.955191	158.79	29.7759	0.457803	0.104355	1.14117	0.2244
237	Fresh Olive	June	100	5202	0.149884	4.27697	1.9085	0.096835	101.345	-8.07	25.955	2.55285	151.935	38.2463	0.570617	1.1784	0.424382	0.0168465	0.117872	0.549258	0.138802	0.253168	0.0227108	1.74216	1.46543	0.270637	12.287	0.413098	23.0732	3.02512	5.24319	4.11652	1.32017	0.912201	95.387	22.5681	0.535381	0.066121	0.19792	0.104262
238	Fresh Olive	June	100	5202	0.106247	4.20704	2.08748	0.0957298	100.29	-9.825	32.645	3.32005	133.253	38.2463	0.570617	1.1784	0.424382	0.0168465	0.117872	0.549258	0.138802	0.253168	0.0227108	1.74216	1.46543	0.270637	12.287	0.413098	23.0732	3.02512	5.24319	4.11652	1.32017	0.912201	95.387	22.5681	0.535381	0.066121	0.19792	0.104262
239	Fresh Olive	June	100	5202	0.128066	4.242	1.99799	0.0962824	100.817	-8.9475	29.3	2.93645	142.594	38.2463	0.570617	1.1784	0.424382	0.0168465	0.117872	0.549258	0.138802	0.253168	0.0227108	1.74216	1.46543	0.270637	12.287	0.413098	23.0732	3.02512	5.24319	4.11652	1.32017	0.912201	95.387	22.5681	0.535381	0.066121	0.19792	0.104262
240	Fresh Olive	June	100	5202	0.117156	4.22452	2.04274	0.0960061	100.554	-9.38625	30.9725	3.12825	137.923	38.2463	0.570617	1.1784	0.424382	0.0168465	0.117872	0.549258	0.138802	0.253168	0.0227108	1.74216	1.46543	0.270637	12.287	0.413098	23.0732	3.02512	5.24319	4.11652	1.32017	0.912201	95.387	22.5681	0.535381	0.066121	0.19792	0.104262
241	Fresh Olive	June	175	5202	0.105337		2.36561	0.102304	89.585	-4.72	23.93	3.61462	133.382	86.7235	0.395136	1.49666	0.361955	0.0182119	0.101878	0.652178	0.233063	0.297158	0.0435565	0.548628	0.47352	0.248126	12.5923	0.312263	23.8219	2.64973	5.51916	4.92347	1.31597	1.2631	143.991	21.5579	0.511766	0.0663406	0.281116	0.110086
242	Fresh Olive	June	175	5202	0.102177	0	2.29464	0.0992349	86.8975	-4.5784	23.2121	3.50618	129.381	86.7235	0.395136	1.49666	0.361955	0.0182119	0.101878	0.652178	0.233063	0.297158	0.0435565	0.548628	0.47352	0.248126	12.5923	0.312263	23.8219	2.64973	5.51916	4.92347	1.31597	1.2631	143.991	21.5579	0.511766	0.0663406	0.281116	0.110086
243	Fresh Olive	June	175	5202	0.109024	0	2.44841	0.105885	92.7205	-4.8852	24.7675	3.74113	138.05	86.7235	0.395136	1.49666	0.361955	0.0182119	0.101878	0.652178	0.233063	0.297158	0.0435565	0.548628	0.47352	0.248126	12.5923	0.312263	23.8219	2.64973	5.51916	4.92347	1.31597	1.2631	143.991	21.5579	0.511766	0.0663406	0.281116	0.110086
244	Fresh Olive	June	175	5202	0.105753	0	2.37495	0.102708	89.9389	-4.73864	24.0245	3.6289	133.909	86.7235	0.395136	1.49666	0.361955	0.0182119	0.101878	0.652178	0.233063	0.297158	0.0435565	0.548628	0.47352	0.248126	12.5923	0.312263	23.8219	2.64973	5.51916	4.92347	1.31597	1.2631	143.991	21.5579	0.511766	0.0663406	0.281116	0.110086
245	Fresh Olive	June	175	5202	0.0809613	3.79689	1.96986	0.0802982	95.53	-9.295	33.145	3.29089	126.756	116.162	0.570974	1.52927	0.81378	0.0171628	0.124526	0.60915	0.377909	0.319603	0.0439373	1.91987	1.16437	0.296268	12.7078	0.208843	26.8121	3.50339	3.12871	3.38074	0.780743	0.889408	175.361	21.5561	0.355272	0.0794494	0.283011	0.137173
246	Fresh Olive	June	175	5202	0.0926052	3.03721	1.75048	0.0750676	99.5	-12.285	43.9	4.18295	148.653	116.162	0.570974	1.52927	0.81378	0.0171628	0.124526	0.60915	0.377909	0.319603	0.0439373	1.91987	1.16437	0.296268	12.7078	0.208843	26.8121	3.50339	3.12871	3.38074	0.780743	0.889408	175.361	21.5561	0.355272	0.0794494	0.283011	0.137173
247	Fresh Olive	June	175	5202	0.089827	2.94609	1.69797	0.0728156	96.515	-11.9164	42.583	4.05746	144.193	116.162	0.570974	1.52927	0.81378	0.0171628	0.124526	0.60915	0.377909	0.319603	0.0439373	1.91987	1.16437	0.296268	12.7078	0.208843	26.8121	3.50339	3.12871	3.38074	0.780743	0.889408	175.361	21.5561	0.355272	0.0794494	0.283011	0.137173
248	Fresh Olive	June	175	5202	0.0958464	3.14351	1.81175	0.077695	102.982	-12.715	45.4365	4.32935	153.856	116.162	0.570974	1.52927	0.81378	0.0171628	0.124526	0.60915	0.377909	0.319603	0.0439373	1.91987	1.16437	0.296268	12.7078	0.208843	26.8121	3.50339	3.12871	3.38074	0.780743	0.889408	175.361	21.5561	0.355272	0.0794494	0.283011	0.137173
249	Fresh Olive	June	250	5202	0.105768	5.75965	2.17597	0.104299	20.625	-1.73	10.99	2.26656	154.957	16.9062	0.448265	2.70367	1.06265	0.0189533	0.189254	0.85975	0.172174	0.492456	0.15425	0.875855	0.129736	0.649675	5.65109	0.284105	25.0321	3.68719	2.28558	2.03046	0.524731	0.772445	64.9306	13.1494	0.385673	0.101887	1.48341	0.12405
250	Fresh Olive	June	250	5202	0.102595	5.58686	2.11069	0.10117	20.0063	-1.6781	10.6603	2.19856	150.308	16.9062	0.448265	2.70367	1.06265	0.0189533	0.189254	0.85975	0.172174	0.492456	0.15425	0.875855	0.129736	0.649675	5.65109	0.284105	25.0321	3.68719	2.28558	2.03046	0.524731	0.772445	64.9306	13.1494	0.385673	0.101887	1.48341	0.12405
251	Fresh Olive	June	250	5202	0.10947	5.96124	2.25213	0.107949	21.3469	-1.79055	11.3747	2.34589	160.38	16.9062	0.448265	2.70367	1.06265	0.0189533	0.189254	0.85975	0.172174	0.492456	0.15425	0.875855	0.129736	0.649675	5.65109	0.284105	25.0321	3.68719	2.28558	2.03046	0.524731	0.772445	64.9306	13.1494	0.385673	0.101887	1.48341	0.12405
252	Fresh Olive	June	250	5202	0.106186	5.7824	2.18457	0.104711	20.7065	-1.73683	11.0334	2.27551	155.569	16.9062	0.448265	2.70367	1.06265	0.0189533	0.189254	0.85975	0.172174	0.492456	0.15425	0.875855	0.129736	0.649675	5.65109	0.284105	25.0321	3.68719	2.28558	2.03046	0.524731	0.772445	64.9306	13.1494	0.385673	0.101887	1.48341	0.12405
253	Fresh Olive	June	0	6959	0.118103	1.59942	1.93103	0.0884903	102.44	-7.34	22.74	5.46882	125.538	88.0187	0.845209	1.66378	0.221803	0.0303826	0.132486	1.04514	0.266967	0.238016	1.02856	33.687	2.70181	0.0936165	54.1048	1.59142	23.2957	2.44686	15.0353	6.42407	1.25313	1.6004	235.725	105.368	0.485026	0.0841638	0.320388	0.140114
254	Fresh Olive	June	0	6959	0.127328	1.6177	2.05078	0.094451	100.13	-7.09	22.16		123.032	88.0187	0.845209	1.66378	0.221803	0.0303826	0.132486	1.04514	0.266967	0.238016	1.02856	33.687	2.70181	0.0936165	54.1048	1.59142	23.2957	2.44686	15.0353	6.42407	1.25313	1.6004	235.725	105.368	0.485026	0.0841638	0.320388	0.140114
255	Fresh Olive	June	0	6959	0.0817044	1.29943	2.028	0.0990352	102.33	-9.63	30.73	5.2009	114.97	88.0187	0.845209	1.66378	0.221803	0.0303826	0.132486	1.04514	0.266967	0.238016	1.02856	33.687	2.70181	0.0936165	54.1048	1.59142	23.2957	2.44686	15.0353	6.42407	1.25313	1.6004	235.725	105.368	0.485026	0.0841638	0.320388	0.140114
256	Fresh Olive	June	0	6959	0.0792747	1.4394	1.90909	0.100527	100.47	-9.28	29.44		116.804	88.0187	0.845209	1.66378	0.221803	0.0303826	0.132486	1.04514	0.266967	0.238016	1.02856	33.687	2.70181	0.0936165	54.1048	1.59142	23.2957	2.44686	15.0353	6.42407	1.25313	1.6004	235.725	105.368	0.485026	0.0841638	0.320388	0.140114
257	Fresh Olive	June	0	6959	0.0804896	1.36941	1.96855	0.0997811	101.4	-9.455	30.085	5.2009	115.887	88.0187	0.845209	1.66378	0.221803	0.0303826	0.132486	1.04514	0.266967	0.238016	1.02856	33.687	2.70181	0.0936165	54.1048	1.59142	23.2957	2.44686	15.0353	6.42407	1.25313	1.6004	235.725	105.368	0.485026	0.0841638	0.320388	0.140114
258	Fresh Olive	June	100	6959	0.0834995	2.65468	1.96057	0.079982	94.135	-9.5	33.22	3.69027	137.279	128.363	0.889787	1.51226	0.388236	0.0324082	0.14639	0.740479	0.514017	0.275514	0.0175759	15.8686	2.3979	0.197659	27.1025	1.04989	27.3075	3.50369	3.43111	5.99349	1.42692	1.48287	222.642	50.1035	0.257349	0.0869424	0.309658	0.15194
259	Fresh Olive	June	100	6959	0.127397	2.85523	1.85357	0.081799	101.05	-10.36	35.575	3.76078	117.822	128.363	0.889787	1.51226	0.388236	0.0324082	0.14639	0.740479	0.514017	0.275514	0.0175759	15.8686	2.3979	0.197659	27.1025	1.04989	27.3075	3.50369	3.43111	5.99349	1.42692	1.48287	222.642	50.1035	0.257349	0.0869424	0.309658	0.15194
260	Fresh Olive	June	100	6959	0.105448	2.75496	1.90707	0.0808905	97.5925	-9.93	34.3975	3.72552	127.55	128.363	0.889787	1.51226	0.388236	0.0324082	0.14639	0.740479	0.514017	0.275514	0.0175759	15.8686	2.3979	0.197659	27.1025	1.04989	27.3075	3.50369	3.43111	5.99349	1.42692	1.48287	222.642	50.1035	0.257349	0.0869424	0.309658	0.15194
261	Fresh Olive	June	100	6959	0.116423	2.80509	1.88032	0.0813448	99.3213	-10.145	34.9863	3.74315	122.686	128.363	0.889787	1.51226	0.388236	0.0324082	0.14639	0.740479	0.514017	0.275514	0.0175759	15.8686	2.3979	0.197659	27.1025	1.04989	27.3075	3.50369	3.43111	5.99349	1.42692	1.48287	222.642	50.1035	0.257349	0.0869424	0.309658	0.15194
262	Fresh Olive	June	100	6959	0.100465	4.4475	2.26658	0.091919	101.145	-9.95	35	2.98134	122.9	81.3948	0.546492	1.46898	0.493948	0.334209	0.565356	0.852828	0.249347	0.275881	0.143215	3.15349	1.78346	0.18786	23.4441	1.54788	24.4671	3.13487	2.91225	3.25586	0.9164	0.790428	151.919	32.8325	0.505929	0.0777285	0.223973	0.121467
263	Fresh Olive	June	100	6959	0.0977957	4.31719	1.97978	0.0903771	98.62	-11.435	42.105	2.65954	145.041	81.3948	0.546492	1.46898	0.493948	0.334209	0.565356	0.852828	0.249347	0.275881	0.143215	3.15349	1.78346	0.18786	23.4441	1.54788	24.4671	3.13487	2.91225	3.25586	0.9164	0.790428	151.919	32.8325	0.505929	0.0777285	0.223973	0.121467
264	Fresh Olive	June	100	6959	0.0991303	4.38235	2.12318	0.091148	99.8825	-10.6925	38.5525	2.82044	133.971	81.3948	0.546492	1.46898	0.493948	0.334209	0.565356	0.852828	0.249347	0.275881	0.143215	3.15349	1.78346	0.18786	23.4441	1.54788	24.4671	3.13487	2.91225	3.25586	0.9164	0.790428	151.919	32.8325	0.505929	0.0777285	0.223973	0.121467
265	Fresh Olive	June	100	6959	0.098463	4.34977	2.05148	0.0907626	99.2513	-11.0638	40.3288	2.73999	139.506	81.3948	0.546492	1.46898	0.493948	0.334209	0.565356	0.852828	0.249347	0.275881	0.143215	3.15349	1.78346	0.18786	23.4441	1.54788	24.4671	3.13487	2.91225	3.25586	0.9164	0.790428	151.919	32.8325	0.505929	0.0777285	0.223973	0.121467
266	Fresh Olive	June	175	6959	0.115986	5.62405	2.35843	0.0968916	91.06	-5.975	25.805	3.30822	139.013	29.9287	0.45103	1.48874	0.78183	0.00949733	0.13116	0.548888	0.109613	0.291519	0.0869185	1.13234	0.760089	0.400846	11.9758	0.134373	27.6277	3.45602	3.84794	3.16363	0.674081	0.906301	87.9071	20.813	0.436129	0.0736024	0.288999	0.104669
267	Fresh Olive	June	175	6959	0.109527	4.88873	2.25984	0.12273	97.725	-10.875	41.82	2.98414	144.5	26.7314	0.454395	1.48971	0.772993	0.0137988	0.135546	0.537165	0.159758	0.275692	0.121212	1.91219	1.49296	0.39049	8.88154	0.206385	24.2004	3.19952	4.03166	6.50155	1.77194	1.64671	84.927	18.4154	0.417171	0.0811995	0.249543	0.142048
268	Fresh Olive	June	175	6959	0.0726718	6.88344	2.18526	0.0739581	72.09	-5.58	31.025	3.33842	132.395	93.1276	0.519599	2.06859	0.413486	0.0230326	0.177337	0.750225	0.203739	0.263909	0.0746193	2.70444	3.03796	0.21368	32.8925	0.679002	28.9883	3.36215	3.30523	2.41327	0.428513	0.73101	176.378	42.478	0.460634	0.072397	0.37811	0.0937118
269	Fresh Olive	June	175	6959	0.0697368	5.1622	2.07447	0.0739052	97.935	-10.58	37.885	2.80687	153.303	73.598	0.685169	1.40792	0.391361	0.0204968	0.162776	0.873172	0.190546	0.262609	0.100297	6.24938	0.115521	0.310507	22.0521	0.916515	25.3963	2.96176	3.56074	2.68569	0.639529	0.912642	143.493	35.1345	0.431754	0.0801211	0.389416	0.109854
270	Fresh Olive	June	175	6959	0.125944	2.79935	2.05625	0.108622	98.71	-10.21	36.005	2.61487	154.021	30.5385	0.3522	1.29724	0.970489	0.188432	0.411802	0.542936	0.130036	0.391401	0.0656003	1.52144	0.101357	0.439598	3.93989	0.308126	25.9278	3.59226	3.32708	2.194	0.736697	0.673604	77.6505	12.8203	0.781686	0.0709094	0.252936	0.145746
271	Fresh Olive	June	175	6959	0.075428	3.34906	2.2825	0.0988915	99.785	-8.74	28.085	2.88642	156.007	10.2566	0.344841	1.39571	0.876424	0.0150551	0.137562	0.572651	0.182476	0.335596	0.146277	1.22473	0.117558	0.532134	3.17321	0.118207	24.4326	3.68309	2.40673	2.18538	0.562146	0.817025	53.516	11.0199	0.542384	0.0699453	0.221717	0.116324
272	Fresh Olive	June	250	6959	0.101809	5.76793	2.22945	0.0923022	93.095	-6.015	27.43	2.22511	138.017	56.6745	0.520713	1.96267	0.514221	0.253974	0.574598	0.543886	0.211859	0.254143	0.2223	3.10013	2.71234	0.220759	41.2016	1.81477	27.4435	3.21764	3.06945	3.99053	1.2047	1.03488	150.743	50.8096	1.08492	0.0745823	0.334152	0.104986
273	Fresh Olive	June	250	6959	0.0676982	3.07513	1.88018	0.0783095	100.865	-9.095	30.115	3.36127	120.424	56.6745	0.520713	1.96267	0.514221	0.253974	0.574598	0.543886	0.211859	0.254143	0.2223	3.10013	2.71234	0.220759	41.2016	1.81477	27.4435	3.21764	3.06945	3.99053	1.2047	1.03488	150.743	50.8096	1.08492	0.0745823	0.334152	0.104986
274	Fresh Olive	June	250	6959	0.0847536	4.42153	2.05482	0.0853058	96.98	-7.555	28.7725	2.79319	129.221	56.6745	0.520713	1.96267	0.514221	0.253974	0.574598	0.543886	0.211859	0.254143	0.2223	3.10013	2.71234	0.220759	41.2016	1.81477	27.4435	3.21764	3.06945	3.99053	1.2047	1.03488	150.743	50.8096	1.08492	0.0745823	0.334152	0.104986
275	Fresh Olive	June	250	6959	0.0762259	3.74833	1.9675	0.0818077	98.9225	-8.325	29.4438	3.07723	124.822	56.6745	0.520713	1.96267	0.514221	0.253974	0.574598	0.543886	0.211859	0.254143	0.2223	3.10013	2.71234	0.220759	41.2016	1.81477	27.4435	3.21764	3.06945	3.99053	1.2047	1.03488	150.743	50.8096	1.08492	0.0745823	0.334152	0.104986
276	Fresh Olive	June	250	6959	0.0942024	2.94462	1.87805	0.0939757	102.935	-9.505	29.825	2.38225	153.967	29.0595	0.330576	0.980356	0.500452	0.0067073	0.128871	0.553929	0.0570459	0.269305	0.0325203	1.14904	0.77476	0.361509	4.44241	0.29531	24.5142	2.93716	2.34991	2.10431	0.582759	0.728172	72.1588	11.24	0.621555	0.0678525	0.16704	0.122886
277	Fresh Olive	June	250	6959	0.117612	2.50899	1.99267	0.0895165	102.465	-11.02	34.775	2.31683	157.772	29.0595	0.330576	0.980356	0.500452	0.0067073	0.128871	0.553929	0.0570459	0.269305	0.0325203	1.14904	0.77476	0.361509	4.44241	0.29531	24.5142	2.93716	2.34991	2.10431	0.582759	0.728172	72.1588	11.24	0.621555	0.0678525	0.16704	0.122886
278	Fresh Olive	June	250	6959	0.105907	2.7268	1.93536	0.0917461	102.7	-10.2625	32.3	2.34954	155.87	29.0595	0.330576	0.980356	0.500452	0.0067073	0.128871	0.553929	0.0570459	0.269305	0.0325203	1.14904	0.77476	0.361509	4.44241	0.29531	24.5142	2.93716	2.34991	2.10431	0.582759	0.728172	72.1588	11.24	0.621555	0.0678525	0.16704	0.122886
279	Fresh Olive	June	250	6959	0.11176	2.6179	1.96402	0.0906313	102.582	-10.6412	33.5375	2.33318	156.821	29.0595	0.330576	0.980356	0.500452	0.0067073	0.128871	0.553929	0.0570459	0.269305	0.0325203	1.14904	0.77476	0.361509	4.44241	0.29531	24.5142	2.93716	2.34991	2.10431	0.582759	0.728172	72.1588	11.24	0.621555	0.0678525	0.16704	0.122886
280	Fresh Olive	June	100	8718	0.0772716	5.18102	1.98171	0.0935974	102.535	-10.175	35.69	3.53778	131.927	68.5627	0.432128	1.52022	0.709747	0.0854726	0.738535	1.00449	0.164897	0.302932	0.113221	4.37471	0.839738	0.174919	18.5875	1.59884	26.8862	3.27883	2.82024	2.0609	0.583996	0.847482	135.688	29.2362	0.52468	0.081798	0.467609	0.129991
281	Fresh Olive	June	100	8718	0.0784184	3.44028	2.01176	0.0927321	101.54	-9.795	31.34	3.31882	174.152	68.5627	0.432128	1.52022	0.709747	0.0854726	0.738535	1.00449	0.164897	0.302932	0.113221	4.37471	0.839738	0.174919	18.5875	1.59884	26.8862	3.27883	2.82024	2.0609	0.583996	0.847482	135.688	29.2362	0.52468	0.081798	0.467609	0.129991
282	Fresh Olive	June	100	8718	0.077845	4.31065	1.99673	0.0931647	102.037	-9.985	33.515	3.4283	153.04	68.5627	0.432128	1.52022	0.709747	0.0854726	0.738535	1.00449	0.164897	0.302932	0.113221	4.37471	0.839738	0.174919	18.5875	1.59884	26.8862	3.27883	2.82024	2.0609	0.583996	0.847482	135.688	29.2362	0.52468	0.081798	0.467609	0.129991
283	Fresh Olive	June	100	8718	0.0781317	3.87546	2.00425	0.0929484	101.789	-9.89	32.4275	3.37356	163.596	68.5627	0.432128	1.52022	0.709747	0.0854726	0.738535	1.00449	0.164897	0.302932	0.113221	4.37471	0.839738	0.174919	18.5875	1.59884	26.8862	3.27883	2.82024	2.0609	0.583996	0.847482	135.688	29.2362	0.52468	0.081798	0.467609	0.129991
284	Fresh Olive	June	175	8718	0.074307	2.50734	1.69862	0.0621251	100.945	-11.99	41.025	3.07842	162.868	36.4469	0.497331	1.29112	1.1217	0.0134485	0.124474	0.747421	0.219425	0.316206	0.0273654	3.16668	1.43027	0.356636	8.87974	0.856774	25.9008	4.22509	2.0273	2.02422	0.716698	0.86953	91.2591	18.6554	0.300739	0.0774972	0.213643	0.158286
285	Fresh Olive	June	175	8718	0.112361	4.96861	2.21021	0.0806554	75.025	-6.075	32.52	2.98294	128.334	36.4469	0.497331	1.29112	1.1217	0.0134485	0.124474	0.747421	0.219425	0.316206	0.0273654	3.16668	1.43027	0.356636	8.87974	0.856774	25.9008	4.22509	2.0273	2.02422	0.716698	0.86953	91.2591	18.6554	0.300739	0.0774972	0.213643	0.158286
286	Fresh Olive	June	175	8718	0.093334	3.73797	1.95441	0.0713902	87.985	-9.0325	36.7725	3.03068	145.601	36.4469	0.497331	1.29112	1.1217	0.0134485	0.124474	0.747421	0.219425	0.316206	0.0273654	3.16668	1.43027	0.356636	8.87974	0.856774	25.9008	4.22509	2.0273	2.02422	0.716698	0.86953	91.2591	18.6554	0.300739	0.0774972	0.213643	0.158286
287	Fresh Olive	June	175	8718	0.102848	4.35329	2.08231	0.0760228	81.505	-7.55375	34.6463	3.00681	136.967	36.4469	0.497331	1.29112	1.1217	0.0134485	0.124474	0.747421	0.219425	0.316206	0.0273654	3.16668	1.43027	0.356636	8.87974	0.856774	25.9008	4.22509	2.0273	2.02422	0.716698	0.86953	91.2591	18.6554	0.300739	0.0774972	0.213643	0.158286
288	Fresh Olive	June	175	8718	0.127795	4.5624	2.13267	0.0798843	97.995	-6.85	25.58	2.47701	162.161	115.251	0.533913	1.21491	0.205306	0.355568	0.301924	0.537355	0.234127	0.234378	0.0516941	1.585	0.0935285	0.203656	24.5465	1.08585	24.7823	2.39192	10.5094	5.44127	2.60377	1.39713	193.56	39.2365	0.400704	0.0749711	0.238288	0.134304
289	Fresh Olive	June	175	8718	0.0969303	3.24659	1.73615	0.077077	101.33	-11.06	34.745	3.25022	137.342	115.251	0.533913	1.21491	0.205306	0.355568	0.301924	0.537355	0.234127	0.234378	0.0516941	1.585	0.0935285	0.203656	24.5465	1.08585	24.7823	2.39192	10.5094	5.44127	2.60377	1.39713	193.56	39.2365	0.400704	0.0749711	0.238288	0.134304
290	Fresh Olive	June	250	8718	0.105291	4.94674	2.05933	0.0783345	100.165	-7.92	26.25	2.72819	155.039	115.251	0.533913	1.21491	0.205306	0.355568	0.301924	0.537355	0.234127	0.234378	0.0516941	1.585	0.0935285	0.203656	24.5465	1.08585	24.7823	2.39192	10.5094	5.44127	2.60377	1.39713	193.56	39.2365	0.400704	0.0749711	0.238288	0.134304
291	Fresh Olive	June	250	8718	0.0885885	4.87922	2.04511	0.0724449	99.32	-4.555	25.97	3.4341	133.729	115.251	0.533913	1.21491	0.205306	0.355568	0.301924	0.537355	0.234127	0.234378	0.0516941	1.585	0.0935285	0.203656	24.5465	1.08585	24.7823	2.39192	10.5094	5.44127	2.60377	1.39713	193.56	39.2365	0.400704	0.0749711	0.238288	0.134304
292	Fresh Olive	July	100	5202	0.130003	5.27157	2.13193	0.08699	103.26	-7.97	27.425	2.99464	124.033	41.2424	0.378743	1.20606	0.788982	0.104638	0.11561	0.616994	0.124892	0.260325	0.090365	0.180583	0.479513	0.212106	5.51238	0.19602	25.4922	1.18665	7.78655	3.81559	1.23489	0.983973	92.0095	14.8783	0.394579	0.070719	0.30402	0.123506
293	Fresh Olive	July	100	5202	0.237293	5.41736	2.44371	0.104706	97.545	-7.855	29.815	2.98147	121.172	41.2424	0.378743	1.20606	0.788982	0.104638	0.11561	0.616994	0.124892	0.260325	0.090365	0.180583	0.479513	0.212106	5.51238	0.19602	25.4922	1.18665	7.78655	3.81559	1.23489	0.983973	92.0095	14.8783	0.394579	0.070719	0.30402	0.123506
294	Fresh Olive	July	100	5202										41.2424	0.378743	1.20606	0.788982	0.104638	0.11561	0.616994	0.124892	0.260325	0.090365	0.180583	0.479513	0.212106	5.51238	0.19602	25.4922	1.18665	7.78655	3.81559	1.23489	0.983973	92.0095	14.8783	0.394579	0.070719	0.30402	0.123506
295	Fresh Olive	July	100	5202										41.2424	0.378743	1.20606	0.788982	0.104638	0.11561	0.616994	0.124892	0.260325	0.090365	0.180583	0.479513	0.212106	5.51238	0.19602	25.4922	1.18665	7.78655	3.81559	1.23489	0.983973	92.0095	14.8783	0.394579	0.070719	0.30402	0.123506
296	Fresh Olive	July	175	5202										41.0507	0.889697	1.54631	1.08244	0.0585181	0.199065	0.571074	0.23734	0.328475	0.0578658	1.89978	0.640284	0.263958	6.95227	0.485182	22.4535	6.73745	1.38124	2.25348	0.709682	0.714476	90.5128	17.2347				
297	Fresh Olive	July	175	5202	0.138548	2.99817	1.81741	0.0753398	101.665	-10.95	36.965	2.64939		41.0507	0.889697	1.60043	1.12033	0.0605662	0.206032	0.591062	0.245647	0.339972	0.0598911	1.96628	0.662694	0.273197	7.1956	0.502163	23.2393	6.97327	1.42958	2.33235	0.734521	0.739483	93.6807	17.8379				
298	Fresh Olive	July	175	5202										41.0507	0.889697	1.60043	1.12033	0.0605662	0.206032	0.591062	0.245647	0.339972	0.0598911	1.96628	0.662694	0.273197	7.1956	0.502163	23.2393	6.97327	1.42958	2.33235	0.734521	0.739483	93.6807	17.8379				
299	Fresh Olive	July	175	5202										41.0507	0.889697	1.65644	1.15954	0.0626861	0.213243	0.611749	0.254245	0.351871	0.0619873	2.0351	0.685889	0.282759	7.44744	0.519739	24.0527	7.21733	1.47962	2.41398	0.760229	0.765365	96.9595	18.4622				
300	Fresh Olive	July	175	5202	0.114473	3.01739	1.95558	0.0800057	100.96	-8.805	27.36	3.03934	136.888	55.1242	0.304415	1.14921	0.848342	0.376784	0.388731	0.531028	0.134097	0.328567	0.0451503	1.16957	0.814404	0.472921	9.60055	1.17315	32.1625	3.86034	7.99214	4.2705	1.35844	1.18676	123.292	23.0955	0.430787	0.0621015	0.191556	0.0988376
301	Fresh Olive	July	175	5202	0.145707	2.61732	2.08539	0.0748779	100.46	-8.405	26.86	3.21462	138.346	55.1242	0.304415	1.14921	0.848342	0.376784	0.388731	0.531028	0.134097	0.328567	0.0451503	1.16957	0.814404	0.472921	9.60055	1.17315	32.1625	3.86034	7.99214	4.2705	1.35844	1.18676	123.292	23.0955	0.430787	0.0621015	0.191556	0.0988376
302	Fresh Olive	July	175	5202	0.13009	2.81735	2.02048	0.0774418	100.71	-8.605	27.11	3.12698	137.617	55.1242	0.304415	1.14921	0.848342	0.376784	0.388731	0.531028	0.134097	0.328567	0.0451503	1.16957	0.814404	0.472921	9.60055	1.17315	32.1625	3.86034	7.99214	4.2705	1.35844	1.18676	123.292	23.0955	0.430787	0.0621015	0.191556	0.0988376
303	Fresh Olive	July	175	5202	0.137899	2.71734	2.05294	0.0761598	100.585	-8.505	26.985	3.1708	137.982	55.1242	0.304415	1.14921	0.848342	0.376784	0.388731	0.531028	0.134097	0.328567	0.0451503	1.16957	0.814404	0.472921	9.60055	1.17315	32.1625	3.86034	7.99214	4.2705	1.35844	1.18676	123.292	23.0955	0.430787	0.0621015	0.191556	0.0988376
304	Fresh Olive	July	0	6959	0.241553	3.89494	2.36364	0.104287	95.44	-4.83	19.84	3.61988	125.029	79.6165	0.588645	1.19698	0.182386	0.13118	0.10334	2.13765	0.073386	0.236449	0.0762504	41.9529	1.28393	0.201956	41.0746	0.391909	21.6196	1.08225	6.54705	6.17971	1.36208	1.2626	207.301	90.8587	0.401606	0.0832372	0.307719	0.107486
305	Fresh Olive	July	0	6959	0.202785	3.75572	2.47244	0.10082	95.05	-4.84	20.08		122.708	79.6165	0.588645	1.19698	0.182386	0.13118	0.10334	2.13765	0.073386	0.236449	0.0762504	41.9529	1.28393	0.201956	41.0746	0.391909	21.6196	1.08225	6.54705	6.17971	1.36208	1.2626	207.301	90.8587	0.401606	0.0832372	0.307719	0.107486
306	Fresh Olive	July	0	6959	0.170739	2.63747	2.44444	0.102646	100.37	-5.41	18.43	4.08572	109.13	79.6165	0.588645	1.19698	0.182386	0.13118	0.10334	2.13765	0.073386	0.236449	0.0762504	41.9529	1.28393	0.201956	41.0746	0.391909	21.6196	1.08225	6.54705	6.17971	1.36208	1.2626	207.301	90.8587	0.401606	0.0832372	0.307719	0.107486
307	Fresh Olive	July	0	6959	0.158573	2.81769	2.49219	0.100099	98.09	-4.93	20.12		106.937	79.6165	0.588645	1.19698	0.182386	0.13118	0.10334	2.13765	0.073386	0.236449	0.0762504	41.9529	1.28393	0.201956	41.0746	0.391909	21.6196	1.08225	6.54705	6.17971	1.36208	1.2626	207.301	90.8587	0.401606	0.0832372	0.307719	0.107486
308	Fresh Olive	July	100	6959	0.11045	3.46849	2.40876	0.0808023	104	-8.31	24.59	2.83342	135.769	103.114	0.371613	1.10589	0.469657	0.532134	0.328439	0.5348	0.140622	0.268282	0.0356862	0.937236	0.509581	0.225846	9.80535	0.304724	25.8327	0.865283	8.40217	4.89435	1.61556	1.38902	161.683	20.2359	0.433266	0.0632196	0.164432	0.107969
309	Fresh Olive	July	100	6959	0.126971	4.10696	2.25631	0.0809291	101.825	-7.585	26.62	2.92655	121.083	103.114	0.371613	1.10589	0.469657	0.532134	0.328439	0.5348	0.140622	0.268282	0.0356862	0.937236	0.509581	0.225846	9.80535	0.304724	25.8327	0.865283	8.40217	4.89435	1.61556	1.38902	161.683	20.2359	0.433266	0.0632196	0.164432	0.107969
310	Fresh Olive	July	100	6959	0.118711	3.78772	2.33254	0.0808657	102.912	-7.9475	25.605	2.87999	128.426	103.114	0.371613	1.10589	0.469657	0.532134	0.328439	0.5348	0.140622	0.268282	0.0356862	0.937236	0.509581	0.225846	9.80535	0.304724	25.8327	0.865283	8.40217	4.89435	1.61556	1.38902	161.683	20.2359	0.433266	0.0632196	0.164432	0.107969
311	Fresh Olive	July	100	6959	0.122841	3.94734	2.29442	0.0808974	102.369	-7.76625	26.1125	2.90327	124.755	103.114	0.371613	1.10589	0.469657	0.532134	0.328439	0.5348	0.140622	0.268282	0.0356862	0.937236	0.509581	0.225846	9.80535	0.304724	25.8327	0.865283	8.40217	4.89435	1.61556	1.38902	161.683	20.2359	0.433266	0.0632196	0.164432	0.107969
312	Fresh Olive	July	100	6959	0.157679	3.66494	2.29945	0.0809548	102.135	-11.05	39.625	2.74022	144.364	111.289	0.605731	1.25869	0.617227	0.178648	0.327054	0.548704	0.229442	0.280638	0.0470626	2.17168	1.27199	0.23527	8.41086	0.439929	23.709	0.78393	2.56192	4.8523	1.50052	1.16894	162.489	14.1637	0.36867	0.0789937	0.337308	0.128471
313	Fresh Olive	July	100	6959	0.117119	4.558	2.40058	0.0876735	100.97	-11.59	41.19	3.03458	126.866	111.289	0.605731	1.25869	0.617227	0.178648	0.327054	0.548704	0.229442	0.280638	0.0470626	2.17168	1.27199	0.23527	8.41086	0.439929	23.709	0.78393	2.56192	4.8523	1.50052	1.16894	162.489	14.1637	0.36867	0.0789937	0.337308	0.128471
314	Fresh Olive	July	100	6959	0.137399	4.11147	2.35001	0.0843142	101.552	-11.32	40.4075	2.8874	135.615	111.289	0.605731	1.25869	0.617227	0.178648	0.327054	0.548704	0.229442	0.280638	0.0470626	2.17168	1.27199	0.23527	8.41086	0.439929	23.709	0.78393	2.56192	4.8523	1.50052	1.16894	162.489	14.1637	0.36867	0.0789937	0.337308	0.128471
315	Fresh Olive	July	100	6959	0.127259	4.33473	2.3753	0.0859938	101.261	-11.455	40.7988	2.96099	131.24	111.289	0.605731	1.25869	0.617227	0.178648	0.327054	0.548704	0.229442	0.280638	0.0470626	2.17168	1.27199	0.23527	8.41086	0.439929	23.709	0.78393	2.56192	4.8523	1.50052	1.16894	162.489	14.1637	0.36867	0.0789937	0.337308	0.128471
316	Fresh Olive	July	175	6959		5.27157	2.46863	0.0905819	100.275	-5.15	26.185	3.82898	147.205	69.8532	0.603462	1.43057	0.520823	0.0946566	0.0876932	0.903338	0.0624051	0.23867	0.0949317	0.200611	0.58161	0.155399	11.1008	0.140678	23.0021	1.66817	5.80483	3.39533	0.957748	0.849153	121.746	18.9298	0.280819	0.0701829	0.184863	0.077383
317	Fresh Olive	July	175	6959		5.41736																																		
318	Fresh Olive	July	175	6959	0.195032	2.18798	2.00192	0.0795545	102.16	-0.1	21.615	2.96453	169.299	89.4183	0.31161	1.30311	0.940326	0.0260613	0.121961	0.548132	0.118804	0.280554	0.0241035	1.24242	0.514353	0.316012	7.46649	0.400049	28.1329	3.00446	4.06738	1.57531	0.586006	0.733076	141.131	16.0968				
319	Fresh Olive	July	175	6959	0.192824	1.28551	2.16638	0.0864093	97.89	-7	25.82	2.87456	158.065	102	0.297981	1.29842	0.709976	0.0496893	0.101928	0.539353	0.128186	0.28191	0.0476345	0.467618	0.136106	0.194388	6.82261	0.233735	26.9161	2.68444	0.675163	1.99432	0.526154	0.771712	146.877	10.8442	0.300362	0.0639542	0.252284	0.0987338
320	Fresh Olive	July	175	6959	0.111588	3.79721	2.27612	0.0831379	95.79	-7.575	23.14	2.54953		45.5963	0.33028	1.35441	1.34433	0.177592	0.433666	0.553613	0.297584	0.359699	0.0263534	0.809855	0.341881	0.520108	3.8493	0.319652	25.386	3.76818	0.630277	3.38398	1.51005	1.03377	92.0269	9.57772	0.372286	0.0763716	0.251577	0.139182
321	Fresh Olive	July	175	6959	0.113058	3.53873	2.30885	0.0914153	96.545	-9.025	31.365	2.5508	154.69	51.6764	0.370616	1.30055	1.29965	0.124689	0.36528	0.579606	0.299171	0.349264	0.0507202	0.845411	0.27335	0.234854	2.79201	0.221067	23.6058	8.40229	0.990172	3.94641	1.79626	1.126	100.65	13.2647	0.391259	0.083056	0.248275	0.149551
322	Fresh Olive	July	250	6959	0.110218	4.99305	2.48282	0.0909635	25.29	-0.645	11.05	3.31789	117.486	85.7179	0.301438	1.44564	0.45093	0.397355	0.419889	0.533414	0.0791773	0.249036	0.0774692	0.636302	1.11321	0.204011	19.9919	0.470353	27.7173	2.36111	6.85628	3.4891	0.752218	0.888202	154.152	30.0496	0.332999	0.0697932	0.33544	0.127723
323	Fresh Olive	July	250	6959	0.131661	4.56379	2.48321	0.0967646	100.335	-4.765	19.925	3.45853	146.793	85.7179	0.301438	1.44564	0.45093	0.397355	0.419889	0.533414	0.0791773	0.249036	0.0774692	0.636302	1.11321	0.204011	19.9919	0.470353	27.7173	2.36111	6.85628	3.4891	0.752218	0.888202	154.152	30.0496	0.332999	0.0697932	0.33544	0.127723
324	Fresh Olive	July	250	6959	0.12094	4.77842	2.48302	0.0938641	62.8125	-2.705	15.4875	3.38821	132.139	85.7179	0.301438	1.44564	0.45093	0.397355	0.419889	0.533414	0.0791773	0.249036	0.0774692	0.636302	1.11321	0.204011	19.9919	0.470353	27.7173	2.36111	6.85628	3.4891	0.752218	0.888202	154.152	30.0496	0.332999	0.0697932	0.33544	0.127723
325	Fresh Olive	July	250	6959	0.1263	4.6711	2.48311	0.0953143	81.5738	-3.735	17.7062	3.42337	139.466	85.7179	0.301438	1.44564	0.45093	0.397355	0.419889	0.533414	0.0791773	0.249036	0.0774692	0.636302	1.11321	0.204011	19.9919	0.470353	27.7173	2.36111	6.85628	3.4891	0.752218	0.888202	154.152	30.0496	0.332999	0.0697932	0.33544	0.127723
326	Fresh Olive	July	250	6959	0.0959555	4.78706	2.13074	0.0846576	101.925	-8.435	25.915	2.69664	156.21	30.6199	0.299454	1.12981	0.928511	0.0328513	0.601183	0.578267	0.0820586	0.30912	0.105906	0.878181	0.0864015	0.423524	3.28271	0.271652	22.8218	3.27181	0.522698	1.93095	0.607129	0.597949	69.382	8.37893	0.451177	0.079301	0.360558	0.141731
327	Fresh Olive	July	250	6959	0.127503		2.36576	0.0962252	98.885	-6.735	25.375	4.03724	170.295	30.6199	0.299454	1.12981	0.928511	0.0328513	0.601183	0.578267	0.0820586	0.30912	0.105906	0.878181	0.0864015	0.423524	3.28271	0.271652	22.8218	3.27181	0.522698	1.93095	0.607129	0.597949	69.382	8.37893	0.451177	0.079301	0.360558	0.141731
328	Fresh Olive	July	250	6959	0.111729	4.78706	2.24825	0.0904414	100.405	-7.585	25.645	3.36694	163.253	30.6199	0.299454	1.12981	0.928511	0.0328513	0.601183	0.578267	0.0820586	0.30912	0.105906	0.878181	0.0864015	0.423524	3.28271	0.271652	22.8218	3.27181	0.522698	1.93095	0.607129	0.597949	69.382	8.37893	0.451177	0.079301	0.360558	0.141731
329	Fresh Olive	July	250	6959	0.119616	4.78706	2.307	0.0933333	99.645	-7.16	25.51	3.70209	166.774	30.6199	0.299454	1.12981	0.928511	0.0328513	0.601183	0.578267	0.0820586	0.30912	0.105906	0.878181	0.0864015	0.423524	3.28271	0.271652	22.8218	3.27181	0.522698	1.93095	0.607129	0.597949	69.382	8.37893	0.451177	0.079301	0.360558	0.141731
330	Fresh Olive	July	100	8718	0.155415	3.33773	1.83977	0.0820289	101.68	-5.745	18.74	3.94057	130.457	101.853	0.516313	1.61979	1.07043	0.0219074	0.094977	0.712296	0.187178	0.312706	0.0587788	3.31921	0.512051	0.27929	5.76372	0.158478	25.0155	0.176717	3.49454	2.98249	0.805927	0.797104	149.752	13.0335	0.275303	0.0683869	0.340207	0.114633
331	Fresh Olive	July	100	8718	0.396318	2.92329	2.07866	0.0931481	101.38	-7.12	23.12	3.42816	192.662	101.853	0.516313	1.61979	1.07043	0.0219074	0.094977	0.712296	0.187178	0.312706	0.0587788	3.31921	0.512051	0.27929	5.76372	0.158478	25.0155	0.176717	3.49454	2.98249	0.805927	0.797104	149.752	13.0335	0.275303	0.0683869	0.340207	0.114633
332	Fresh Olive	July	100	8718	0.275867	3.13051	1.95922	0.0875885	101.53	-6.4325	20.93	3.68437	161.559	101.853	0.516313	1.61979	1.07043	0.0219074	0.094977	0.712296	0.187178	0.312706	0.0587788	3.31921	0.512051	0.27929	5.76372	0.158478	25.0155	0.176717	3.49454	2.98249	0.805927	0.797104	149.752	13.0335	0.275303	0.0683869	0.340207	0.114633
333	Fresh Olive	July	100	8718	0.336092	3.0269	2.01894	0.0903683	101.455	-6.77625	22.025	3.55626	177.111	101.853	0.516313	1.61979	1.07043	0.0219074	0.094977	0.712296	0.187178	0.312706	0.0587788	3.31921	0.512051	0.27929	5.76372	0.158478	25.0155	0.176717	3.49454	2.98249	0.805927	0.797104	149.752	13.0335	0.275303	0.0683869	0.340207	0.114633
334	Fresh Olive	July	175	8718	0.103372	3.2076	2.15609	0.0851598	98.78	-11.375	45.81	2.73163	129.788	99.0958	0.379659	1.27047	0.847691	0.190139	0.530628	0.549819	0.255881	0.309404	0.0750401	0.906923	0.92736	0.289736	10.9893	1.67042	24.3344	3.73912	2.26769	2.52442	0.975613	0.937767	153.067	18.1928	0.544024	0.0820194	0.36919	0.167122
335	Fresh Olive	July	175	8718	0.0948485	3.14333	2.15488	0.0779596	102.315	-11.235	36.86	2.56838	144.283	99.0958	0.379659	1.27047	0.847691	0.190139	0.530628	0.549819	0.255881	0.309404	0.0750401	0.906923	0.92736	0.289736	10.9893	1.67042	24.3344	3.73912	2.26769	2.52442	0.975613	0.937767	153.067	18.1928	0.544024	0.0820194	0.36919	0.167122
336	Fresh Olive	July	175	8718	0.0991103	3.17547	2.15548	0.0815597	100.548	-11.305	41.335	2.65001	137.036	99.0958	0.379659	1.27047	0.847691	0.190139	0.530628	0.549819	0.255881	0.309404	0.0750401	0.906923	0.92736	0.289736	10.9893	1.67042	24.3344	3.73912	2.26769	2.52442	0.975613	0.937767	153.067	18.1928	0.544024	0.0820194	0.36919	0.167122
337	Fresh Olive	July	175	8718	0.0969794	3.1594	2.15518	0.0797596	101.431	-11.27	39.0975	2.60919	140.659	99.0958	0.379659	1.27047	0.847691	0.190139	0.530628	0.549819	0.255881	0.309404	0.0750401	0.906923	0.92736	0.289736	10.9893	1.67042	24.3344	3.73912	2.26769	2.52442	0.975613	0.937767	153.067	18.1928	0.544024	0.0820194	0.36919	0.167122
338	Fresh Olive	July	175	8718	0.118719	3.45572	2.29979	0.086071	99.31	-7.575	25.18			76.0519	0.482054	1.27921	0.346301	0.254241	0.503626	0.546695	0.116204	0.238157	0.0247444	0.859621	1.68654	0.200271	14.9436	1.13438	20.9577	2.77181	7.18835	4.07852	1.45603	0.836542	135.957	25.9636	0.283849	0.0895171	0.359557	0.13455
339	Fresh Olive	July	175	8718	0.120276	4.47657	2.0816	0.0795703	97.595	-9.735	37.905	3.46159	145.685	76.0519	0.482054	1.27921	0.346301	0.254241	0.503626	0.546695	0.116204	0.238157	0.0247444	0.859621	1.68654	0.200271	14.9436	1.13438	20.9577	2.77181	7.18835	4.07852	1.45603	0.836542	135.957	25.9636	0.283849	0.0895171	0.359557	0.13455
340	Fresh Olive	July	175	8718	0.119498	3.96615	2.1907	0.0828206	98.4525	-8.655	31.5425	3.46159	145.685	76.0519	0.482054	1.27921	0.346301	0.254241	0.503626	0.546695	0.116204	0.238157	0.0247444	0.859621	1.68654	0.200271	14.9436	1.13438	20.9577	2.77181	7.18835	4.07852	1.45603	0.836542	135.957	25.9636	0.283849	0.0895171	0.359557	0.13455
341	Fresh Olive	July	175	8718	0.119887	4.22136	2.13615	0.0811955	98.0238	-9.195	34.7238	3.46159	145.685	76.0519	0.482054	1.27921	0.346301	0.254241	0.503626	0.546695	0.116204	0.238157	0.0247444	0.859621	1.68654	0.200271	14.9436	1.13438	20.9577	2.77181	7.18835	4.07852	1.45603	0.836542	135.957	25.9636	0.283849	0.0895171	0.359557	0.13455
342	Fresh Olive	July	250	8718	0.104689	5.57944	2.22456	0.0816831	100.7	-6.1	21.095	2.28245	156.654	19.9784	0.363145	1.16867	1.02042	0.0346253	0.118938	0.551576	0.107333	0.366544	0.0628933	0.831583	0.119901	0.33551	1.07675	1.4081	24.4304	3.3263	3.69588	2.3274	0.608238	0.670412	62.603	9.26602	1.04068	0.0708598	0.483292	0.138656
343	Fresh Olive	July	250	8718	0.222304	4.33472	2.3773	0.0930033	98.7	-5.825	23.025	3.34982	150.339	19.9784	0.363145	1.16867	1.02042	0.0346253	0.118938	0.551576	0.107333	0.366544	0.0628933	0.831583	0.119901	0.33551	1.07675	1.4081	24.4304	3.3263	3.69588	2.3274	0.608238	0.670412	62.603	9.26602	1.04068	0.0708598	0.483292	0.138656
344	Fresh Olive	July	250	8718	0.163496	4.95708	2.30093	0.0873432	99.7	-5.9625	22.06	2.81613	153.497	19.9784	0.363145	1.16867	1.02042	0.0346253	0.118938	0.551576	0.107333	0.366544	0.0628933	0.831583	0.119901	0.33551	1.07675	1.4081	24.4304	3.3263	3.69588	2.3274	0.608238	0.670412	62.603	9.26602	1.04068	0.0708598	0.483292	0.138656
345	Fresh Olive	July	250	8718	0.1929	4.6459	2.33911	0.0901733	99.2	-5.89375	22.5425	3.08298	151.918	19.9784	0.363145	1.16867	1.02042	0.0346253	0.118938	0.551576	0.107333	0.366544	0.0628933	0.831583	0.119901	0.33551	1.07675	1.4081	24.4304	3.3263	3.69588	2.3274	0.608238	0.670412	62.603	9.26602	1.04068	0.0708598	0.483292	0.138656

l-2-methylbuty	Butyl acetate	Hexanal	methylbutan-1-	methylbutan-1-	(E)-2-hexenal	3-octanone	Octanal	(E)-2-heptenal	Heptan-2-ol	1-Hexanol	Nonanal	(E)-2-nonenal	(E)-2-hexenol	Acetic acid	Propanoic acid	Octan-1-ol	Butanoic acid	Heptanoic acid	Volatile Compo	olatile compou	st defect volati	Fruity	Bitter	Pungent	Frostbitten Olives
0.0160532	0.1019	11.9361	0.154229	0.188127	27.2429	0.192247	0.0580386	0.14985	0.98713	4.89079	0.878153	0.659323	4.76109	1.85702	0.32917	0.480112	0.402987	2.75389	59.0222	48.8308	4.51645	3	1.5	2.5	1.5
0.0160532	0.1019	11.9361	0.154229	0.188127	27.2429	0.192247	0.0580386	0.14985	0.98713	4.89079	0.878153	0.659323	4.76109	1.85702	0.32917	0.480112	0.402987	2.75389	59.0222	48.8308	4.51645	5	1.4	3.2	0
0.0160532	0.1019	11.9361	0.154229	0.188127	27.2429	0.192247	0.0580386	0.14985	0.98713	4.89079	0.878153	0.659323	4.76109	1.85702	0.32917	0.480112	0.402987	2.75389	59.0222	48.8308	4.51645	3	1.5	2.5	1
0.0160532	0.1019	11.9361	0.154229	0.188127	27.2429	0.192247	0.0580386	0.14985	0.98713	4.89079	0.878153	0.659323	4.76109	1.85702	0.32917	0.480112	0.402987	2.75389	59.0222	48.8308	4.51645	1	1.5	2.5	0
0.0193543	0.139962	11.6386	0.17565	0.203205	24.2628	0.192076	0.074421	0.142859	1.64263	5.23662	1.01543	1.12604	4.64672	1.76321	0.341433	0.480691	0.415938	2.7357	57.4481	45.7848	5.38285	4.9	1.1	2.4	0
0.0193543	0.139962	11.6386	0.17565	0.203205	24.2628	0.192076	0.074421	0.142859	1.64263	5.23662	1.01543	1.12604	4.64672	1.76321	0.341433	0.480691	0.415938	2.7357	57.4481	45.7848	5.38285	3.5	1.5	1.8	0
0.0193543	0.139962	11.6386	0.17565	0.203205	24.2628	0.192076	0.074421	0.142859	1.64263	5.23662	1.01543	1.12604	4.64672	1.76321	0.341433	0.480691	0.415938	2.7357	57.4481	45.7848	5.38285	3	2	2	0
0.0193543	0.139962	11.6386	0.17565	0.203205	24.2628	0.192076	0.074421	0.142859	1.64263	5.23662	1.01543	1.12604	4.64672	1.76321	0.341433	0.480691	0.415938	2.7357	57.4481	45.7848	5.38285	3.7	0	0.5	0
0.0161407	0.080457	7.39615	0.151255	0.185317	18.8958	0.192636	0.00699154	0.469238	0.737558	4.03503	0.860018	0.473237	4.4876	1.76602	0.332346	0.497532	0.419098	2.7534	44.8052	34.8146	4.32256	3	3	4	0
0.0161407	0.080457	7.39615	0.151255	0.185317	18.8958	0.192636	0.00699154	0.469238	0.737558	4.03503	0.860018	0.473237	4.4876	1.76602	0.332346	0.497532	0.419098	2.7534	44.8052	34.8146	4.32256	5.3	4.2	5.1	0
0.0161407	0.080457	7.39615	0.151255	0.185317	18.8958	0.192636	0.00699154	0.469238	0.737558	4.03503	0.860018	0.473237	4.4876	1.76602	0.332346	0.497532	0.419098	2.7534	44.8052	34.8146	4.32256	3	2.5	3	2
0.0161407	0.080457	7.39615	0.151255	0.185317	18.8958	0.192636	0.00699154	0.469238	0.737558	4.03503	0.860018	0.473237	4.4876	1.76602	0.332346	0.497532	0.419098	2.7534	44.8052	34.8146	4.32256	1.9	1.7	2.4	2.2
0.036533	0.148799	14.3405	0.201723	0.220656	36.641	0.204638	0.107914	0.229966	0.731848	5.27264	1.59999	0.423212	5.22433	2.02605	0.359878	0.50559	0.39525	2.80878	73.2576	61.4785	5.49548	3.5	2	2	1.5
0.036533	0.148799	14.3405	0.201723	0.220656	36.641	0.204638	0.107914	0.229966	0.731848	5.27264	1.59999	0.423212	5.22433	2.02605	0.359878	0.50559	0.39525	2.80878	73.2576	61.4785	5.49548	2.3	0	0	2
0.036533	0.148799	14.3405	0.201723	0.220656	36.641	0.204638	0.107914	0.229966	0.731848	5.27264	1.59999	0.423212	5.22433	2.02605	0.359878	0.50559	0.39525	2.80878	73.2576	61.4785	5.49548	2.9	2	0.8	1.3
0.0144944	0.0794319	5.67991	0.191485	0.169921	13.6741	0.198431	0.0123166	0.170596	0.573798	3.20448	1.03545	0.811302	4.35603	1.7305	0.322885	0.484381	0.43074	2.91392	37.0528	26.9145	3.9669	3.5	2	3.5	0
0.0144944	0.0794319	5.67991	0.191485	0.169921	13.6741	0.198431	0.0123166	0.170596	0.573798	3.20448	1.03545	0.811302	4.35603	1.7305	0.322885	0.484381	0.43074	2.91392	37.0528	26.9145	3.9669	5	4	5.3	2.1
0.0144944	0.0794319	5.67991	0.191485	0.169921	13.6741	0.198431	0.0123166	0.170596	0.573798	3.20448	1.03545	0.811302	4.35603	1.7305	0.322885	0.484381	0.43074	2.91392	37.0528	26.9145	3.9669	3	0	0	0
0.0144944	0.0794319	5.67991	0.191485	0.169921	13.6741	0.198431	0.0123166	0.170596	0.573798	3.20448	1.03545	0.811302	4.35603	1.7305	0.322885	0.484381	0.43074	2.91392	37.0528	26.9145	3.9669	3.5	3.5	3.5	0
0.0113628	0.0724602	8.31542	0.197778	0.210569	17.2905	0.194727	0.00945325	0.191654	0.857942	4.02474	0.869113	0.708688	5.18259	1.58194	0.316835	0.485207	0.429224	2.72868	44.6673	34.8132	4.01263	4	2.53	3	2
0.0113628	0.0724602	8.31542	0.197778	0.210569	17.2905	0.194727	0.00945325	0.191654	0.857942	4.02474	0.869113	0.708688	5.18259	1.58194	0.316835	0.485207	0.429224	2.72868	44.6673	34.8132	4.01263	3.4	2.9	2	0
0.0113628	0.0724602	8.31542	0.197778	0.210569	17.2905	0.194727	0.00945325	0.191654	0.857942	4.02474	0.869113	0.708688	5.18259	1.58194	0.316835	0.485207	0.429224	2.72868	44.6673	34.8132	4.01263	2.7	2.5	2.8	0.2
0.0113628	0.0724602	8.31542	0.197778	0.210569	17.2905	0.194727	0.00945325	0.191654	0.857942	4.02474	0.869113	0.708688	5.18259	1.58194	0.316835	0.485207	0.429224	2.72868	44.6673	34.8132	4.01263	2.5	3	3	0
0.0136473	0.143581	9.34119	0.0845272	0.0690384	29.6869	0.189541	0.0442464	0.216086	1.22081	4.52485	0.919121	0.63785	5.10345	1.79381	0.321691	0.501576	0.427029	2.81262	59.1054	48.6564	4.79527	3.5	1.5	2.5	0
0.0136473	0.143581	9.34119	0.0845272	0.0690384	29.6869	0.189541	0.0442464	0.216086	1.22081	4.52485	0.919121	0.63785	5.10345	1.79381	0.321691	0.501576	0.427029	2.81262	59.1054	48.6564	4.79527	2.1	1.5	2.6	0
0.0136473	0.143581	9.34119	0.0845272	0.0690384	29.6869	0.189541	0.0442464	0.216086	1.22081	4.52485	0.919121	0.63785	5.10345	1.79381	0.321691	0.501576	0.427029	2.81262	59.1054	48.6564	4.79527	3	2.3	3	0
0.0136473	0.143581	9.34119	0.0845272	0.0690384	29.6869	0.189541	0.0442464	0.216086	1.22081	4.52485	0.919121	0.63785	5.10345	1.79381	0.321691	0.501576	0.427029	2.81262	59.1054	48.6564	4.79527				
0.0163415	0.094751	19.2312	0.205077	0.221182	31.1078	0.196471	0.0442283	0.176892	1.8066	5.63957	1.01103	0.904215	5.02718	1.71867	0.328735	0.480533	0.430469	2.76745	72.6404	61.0058	5.39863	5.1	1.1	2.1	0
0.0163415	0.094751	19.2312	0.205077	0.221182	31.1078	0.196471	0.0442283	0.176892	1.8066	5.63957	1.01103	0.904215	5.02718	1.71867	0.328735	0.480533	0.430469	2.76745	72.6404	61.0058	5.39863	4.5	1.5	1.7	0
0.0163415	0.094751	19.2312	0.205077	0.221182	31.1078	0.196471	0.0442283	0.176892	1.8066	5.63957	1.01103	0.904215	5.02718	1.71867	0.328735	0.480533	0.430469	2.76745	72.6404	61.0058	5.39863	2	2	3.5	0
0.0163415	0.094751	19.2312	0.205077	0.221182	31.1078	0.196471	0.0442283	0.176892	1.8066	5.63957	1.01103	0.904215	5.02718	1.71867	0.328735	0.480533	0.430469	2.76745	72.6404	61.0058	5.39863	3.7	0	0	0
0.0163415	0.094751	19.2312	0.205077	0.221182	31.1078	0.196471	0.0442283	0.176892	1.8066	5.63957	1.01103	0.904215	5.02718	1.71867	0.328735	0.480533	0.430469	2.76745	72.6404	61.0058	5.39863	3.5	1.5	4.5	3.2
0.0163415	0.094751	19.2312	0.205077	0.221182	31.1078	0.196471	0.0442283	0.176892	1.8066	5.63957	1.01103	0.904215	5.02718	1.71867	0.328735	0.480533	0.430469	2.76745	72.6404	61.0058	5.39863	2	1.5	0	1.5
0.0268456	0.0862898	8.93009	0.230194	0.239754	19.8129	0.194711	0.0162398	0.174426	1.30435	4.24159	0.953569	0.716591	5.99625	1.77361	0.33019	0.483025	0.408713	2.81798	50.5503	38.9808	4.80861	3	2	2	1
0.0268456	0.0862898	8.93009	0.230194	0.239754	19.8129	0.194711	0.0162398	0.174426	1.30435	4.24159	0.953569	0.716591	5.99625	1.77361	0.33019	0.483025	0.408713	2.81798	50.5503	38.9808	4.80861	4.8	3.3	3.5	0
0.0268456	0.0862898	8.93009	0.230194	0.239754	19.8129	0.194711	0.0162398	0.174426	1.30435	4.24159	0.953569	0.716591	5.99625	1.77361	0.33019	0.483025	0.408713	2.81798	50.5503	38.9808	4.80861	3	2	2	0.5
0.0268456	0.0862898	8.93009	0.230194	0.239754	19.8129	0.194711	0.0162398	0.174426	1.30435	4.24159	0.953569	0.716591	5.99625	1.77361	0.33019	0.483025	0.408713	2.81798	50.5503	38.9808	4.80861	2	2.5	2.5	0
0.0240042	0.139557	16.9698	0.106231	0.105853	37.9719	0.212515	0.0762577	0.154466	0.218492	4.57607	1.10612	0.781152	4.94353	1.6018	0.314487	0.487493	0.437282	2.74558	74.0662	64.4613	3.64382	3	1.5	2.5	2
0.0240042	0.139557	16.9698	0.106231	0.105853	37.9719	0.212515	0.0762577	0.154466	0.218492	4.57607	1.10612	0.781152	4.94353	1.6018	0.314487	0.487493	0.437282	2.74558	74.0662	64.4613	3.64382	2.91	1.455	2.425	1.94
0.0240042	0.139557	16.9698	0.106231	0.105853	37.9719	0.212515	0.0762577	0.154466	0.218492	4.57607	1.10612	0.781152	4.94353	1.6018	0.314487	0.487493	0.437282	2.74558	74.0662	64.4613	3.64382	3.105	1.5525	2.5875	2.07
0.0240042	0.139557	16.9698	0.106231	0.105853	37.9719	0.212515	0.0762577	0.154466	0.218492	4.57607	1.10612	0.781152	4.94353	1.6018	0.314487	0.487493	0.437282	2.74558	74.0662	64.4613	3.64382	3.01185	1.50593	2.50988	2.0079
0.0146012	0.0884631	12.0708	0.1482	0.170857	24.2663	0.190442	0.0770634	0.20246	0.20246	4.76434	0.884875	0.791199	4.78912	1.73622	0.319421	0.481683	0.401237	2.75999	55.2868	45.8906	3.64086	3.5	2.5	3.5	0
0.0146012	0.0884631	12.0708	0.1482	0.170857	24.2663	0.190442	0.0770634	0.20246	0.20246	4.76434	0.884875	0.791199	4.78912	1.73622	0.319421	0.481683	0.401237	2.75999	55.2868	45.8906	3.64086	5.2	2.2	5.1	2.1
0.0146012	0.0884631	12.0708	0.1482	0.170857	24.2663	0.190442	0.0770634	0.20246	0.20246	4.76434	0.884875	0.791199	4.78912	1.73622	0.319421	0.481683	0.401237	2.75999	55.2868	45.8906	3.64086	3	2.1	3.6	0
0.0146012	0.0884631	12.0708	0.1482	0.170857	24.2663	0.190442	0.0770634	0.20246	0.20246	4.76434	0.884875	0.791199	4.78912	1.73622	0.319421	0.481683	0.401237	2.75999	55.2868	45.8906	3.64086	2.5	4	3.5	0
0.0295559	0.132995	20.8024	0.261096	0.355119	33.9672	0.198311	0.0786161	0.15706	2.91536	10.9078	0.890435	0.728033	11.7605	1.55484	0.299525	0.480916	0.41657	2.82114	91.2587	77.4379	6.84645	2.5	2	2.5	1
0.0295559	0.132995	20.8024	0.261096	0.355119	33.9672	0.198311	0.0786161	0.15706	2.91536	10.9078	0.890435	0.728033	11.7605	1.55484	0.299525	0.480916	0.41657	2.82114	91.2587	77.4379	6.84645	2	1	1	1.5
0.0295559	0.132995	20.8024	0.261096	0.355119	33.9672	0.198311	0.0786161	0.15706	2.91536	10.9078	0.890435	0.728033	11.7605	1.55484	0.299525	0.480916	0.41657	2.82114	91.2587	77.4379	6.84645				
0.0295559	0.132995	20.8024	0.261096	0.355119	33.9672	0.198311	0.0786161	0.15706	2.91536	10.9078	0.890435	0.728033	11.7605	1.55484	0.299525	0.480916	0.41657	2.82114	91.2587	77.4379	6.84645	2.9	1.5	2.4	1.2
0.0324261	0.183209	19.4852	0.0835156	0.0804399	46.2607	0.200199	0.0805122	0.246059	1.51822	5.28124	0.937407	0.530668	5.11068	1.84765	0.344781	0.510336	0.444674	2.78915	87.1451	76.1378	5.21097	2	1.5	1.5	2
0.0324261	0.183209	19.4852	0.0835156	0.0804399	46.2607	0.200199	0.0805122	0.246059	1.51822	5.28124	0.937407	0.530668	5.11068	1.84765	0.344781	0.510336	0.444674	2.78915	87.1451	76.1378	5.21097	2.5	1	3	2.2
0.0324261	0.183209	19.4852	0.0835156	0.0804399	46.2607	0.200199	0.0805122	0.246059	1.51822	5.28124	0.937407	0.530668	5.11068	1.84765	0.344781	0.510336	0.444674	2.78915	87.1451	76.1378	5.21097				
0.0324261	0.183209	19.4852	0.0835156	0.0804399	46.2607	0.200199	0.0805122	0.246059	1.51822	5.28124	0.937407	0.530668	5.11068	1.84765	0.344781	0.510336	0.444674	2.78915	87.1451	76.1378	5.21097	1.5	0	3.5	4
0.0241543	0.209859	19.6966	0.196138	0.208911	48.6259	0.229428	0.0680519	0.141299	1.36543	5.57026	1.3596	0.795594	5.7151	2.02763	0.370629	0.496414	0.416148	2.92392	92.7362	79.6079	5.81798	3.5	2	3	3
0.0241543	0.209859	19.6966	0.196138	0.208911	48.6259	0.229428	0.0680519	0.141299	1.36543	5.57026	1.3596	0.795594	5.7151	2.02763	0.370629	0.496414	0.416148	2.92392	92.7362	79.6079	5.81798	2	0.1	0.2	3
0.0241543	0.209859	19.6966	0.196138	0.208911	48.6259	0.229428	0.0680519	0.141299	1.36543	5.57026	1.3596	0.795594	5.7151	2.02763	0.370629	0.496414	0.416148	2.92392	92.7362	79.6079	5.81798				
0.0241543	0.209859	19.6966	0.196138	0.208911	48.6259	0.229428	0.0680519	0.141299	1.36543	5.57026	1.3596	0.795594	5.7151	2.02763	0.370629	0.496414	0.416148	2.92392	92.7362	79.6079	5.81798	3	0.9	0.6	1.9
0.0132005	0.0825549	12.5385	0.0807161	0.0899508	22.0324	0.199147	0.0095083	0.182338	0.151822	4.32248	1.14816	0.847053	4.76417	1.46151	0.30089	0.488609	0.340785	2.72948	52.4465	43.6576	3.24367	2.6	1	1	1
0.0132005	0.0825549	12.5385	0.0807161	0.0899508	22.0324	0.199147	0.0095083	0.182338	0.151822	4.32248	1.14816	0.847053	4.76417	1.46151	0.30089	0.488609	0.340785	2.72948	52.4465	43.6576	3.24367	1.5	0	0	1
0.0132005	0.0825549	12.5385	0.0807161	0.0899508	22.0324	0.199147	0.0095083	0.182338	0.151822	4.32248	1.14816	0.847053	4.76417	1.46151	0.30089	0.488609	0.340785	2.72948	52.4465	43.6576	3.24367	2.38056	0.9156	0.9156	0.9156
0.0132005	0.0825549	12.5385	0.0807161	0.0899508	22.0324	0.199147	0.0095083	0.182338	0.151822	4.32248	1.14816	0.847053	4.76417	1.46151	0.30089	0.488609	0.340785	2.72948	52.4465	43.6576	3.24367	1.3734	0	0	0.9156
0.0121781	0.0803708	18.2072	0.158795	0.1447	26.8381	0.195422	0.0157642	0.215577	0.382359	5.96417	1.01281	0.439386	6.00771	1.53981	0.293651	0.476467	0.337995	2.70251	66.0905	57.0172	3.53295	3.5	1.5	1.5	1
0.0121781	0.0803708	18.2072	0.158795	0.1447	26.8381	0.195422	0.0157642	0.215577	0.382359	5.96417	1.01281	0.439386	6.00771	1.53981	0.293651	0.476467	0.337995	2.70251	66.0905	57.0172	3.53295	2.1	0.7	1.4	0.9
0.0121781	0.0803708	18.2072	0.158795	0.1447	26.8381	0.195422	0.0157642	0.215577	0.382359	5.96417	1.01281	0.439386	6.00771	1.53981	0.293651	0.476467	0.337995	2.70251	66.0905	57.0172	3.53295	3.2046	1.3734	1.3734	0.9156
0.0121781	0.0803708	18.2072	0.158795	0.1447	26.8381	0.195422	0.0157642	0.215577	0.382359	5.96417	1.01281	0.439386	6.00771	1.53981	0.293651	0.476467	0.337995	2.70251	66.0905	57.0172	3.53295	3.6	1.3	2.7	1
0.013353	0.104068	12.7293	0.0767483	0.073088	23.8378	0.197899	0.0246566	0.684431	1.4636	2.85849	0.881582	0.397486	4.70355	1.53007	0.308105	0.480953	0.337973	2.71422	54.2218	44.1292	4.90327	3	2	2.5	2.5
0.013353	0.104068	12.7293	0.0767483	0.073088	23.8378	0.197899	0.0246566	0.684431	1.4636	2.85849	0.881582	0.397486	4.70355	1.53007	0.308105	0.480953	0.337973	2.71422	54.2218	44.1292	4.90327	3.29616	1.19028	2.47212	0.9156
0.013353	0.104068	12.7293	0.0767483	0.073088	23.8378	0.197899	0.0246566	0.684431	1.4636	2.85849	0.881582	0.397486	4.70355	1.53007	0.308105	0.480953	0.337973	2.71422	54.2218	44.1292	4.90327	2.7468	1.8312	2.289	2.289
0.013353	0.104068	12.7293	0.0767483	0.073088	23.8378	0.197899	0.0246566	0.684431	1.4636	2.85849	0.881582	0.397486	4.70355	1.53007	0.308105	0.480953	0.337973	2.71422	54.2218	44.1292	4.90327	2.1	1	0.6	1.9
0.0160283	0.122557	10.7662	0.128298	0.114436	25.5813	0.193704	0.0607894	0.272274	1.12051	3.88365	1.12559	0.688239	4.91807	1.4989	0.309206	0.494251	0.342756	2.7409	55.5045	45.1492	4.52523	1.1	1	2.3	1.2
0.0160283	0.122557	10.7662	0.128298	0.114436	25.5813	0.193704	0.0607894	0.272274	1.12051	3.88365	1.12559	0.688239	4.91807	1.4989	0.309206	0.494251	0.342756	2.7409	55.5045	45.1492	4.52523	4	4	4	3
0.0160283	0.122557	10.7662	0.128298	0.114436	25.5813	0.193704	0.0607894	0.272274	1.12051	3.88365	1.12559	0.688239	4.91807	1.4989	0.309206	0.494251	0.342756	2.7409	55.5045	45.1492	4.52523	2.8	0.5	0.6	2.5
0.0160283	0.122557	10.7662	0.128298	0.114436	25.5813	0.193704	0.0607894	0.272274	1.12051	3.88365	1.12559	0.688239	4.91807	1.4989	0.309206	0.494251	0.342756	2.7409	55.5045	45.1492	4.52523	4.1	1	1.7	0
0.0160283	0.122557	10.7662	0.128298	0.114436	25.5813	0.193704	0.0607894	0.272274	1.12051	3.88365	1.12559	0.688239	4.91807	1.4989	0.309206	0.494251	0.342756	2.7409	55.5045	45.1492	4.52523	3.3	1.5	1.9	2.5
0.0169508	0.141261	11.7543	0.0537018	0.0518857	25.7121	0.195488	0.0286949	0.193338	1.21234	3.69865	1.01481	0.637607	4.53602	1.51375	0.306758	0.481644	0.340257	2.70529	55.3901	45.7011	4.25379	2.8	0	0	2.6
0.0169508	0.141261	11.7543	0.0537018	0.0518857	25.7121	0.195488	0.0286949	0.193338	1.21234	3.69865	1.01481	0.637607	4.53602	1.51375	0.306758	0.481644	0.340257	2.70529	55.3901	45.7011	4.25379	3.02148	1.3734	1.73964	2.289
0.0169508	0.141261	11.7543	0.0537018	0.0518857	25.7121	0.195488	0.0286949	0.193338	1.21234	3.69865	1.01481	0.637607	4.53602	1.51375	0.306758	0.481644	0.340257	2.70529	55.3901	45.7011	4.25379	2.1	1	0.6	1.9
0.0169508	0.141261	11.7543	0.0537018	0.0518857	25.7121	0.195488	0.0286949	0.193338	1.21234	3.69865	1.01481	0.637607	4.53602	1.51375	0.306758	0.481644	0.340257	2.70529	55.3901	45.7011	4.25379	3.1	1	1.5	2.5
0.0149655	0.0908553	17.0281	0.0888928	0.0886019	29.0665	0.200979	0.0530093	0.277312	1.91008	4.58116	1.00611	0.634587	5.42227	1.56867	0.306438	0.485944	0.340973	2.71542	66.6005	56.098	5.12078	2.3	1	1.8	2.5
0.0149655	0.0908553	17.0281	0.0888928	0.0886019	29.0665	0.200979	0.0530093	0.277312	1.91008	4.58116	1.00611	0.634587	5.42227	1.56867	0.306438	0.485944	0.340973	2.71542	66.6005	56.098	5.12078	2.1	1	0.6	1.9
0.0149655	0.0908553	17.0281	0.0888928	0.0886019	29.0665	0.200979	0.0530093	0.277312	1.91008	4.58116	1.00611	0.634587	5.42227	1.56867	0.306438	0.485944	0.340973	2.71542	66.6005	56.098	5.12078	3.1	1	1.5	2.5
0.0149655	0.0908553	17.0281	0.0888928	0.0886019	29.0665	0.200979	0.0530093	0.277312	1.91008	4.58116	1.00611	0.634587	5.42227	1.56867	0.306438	0.485944	0.340973	2.71542	66.6005	56.098	5.12078	1.8	0	1	2.1
0.0114978	0.0528276	13.9289	0.169798	0.19645	17.7757	0.204654	0.176948	0.31701	0.0662562	6.78841	1.04162	0.772124	7.19415	1.48869	0.306182	0.486574	0.343604	2.73564	55.0924	45.6872	3.46237	1.64808	0	0.9156	1.92276
0.00941096	0.0600564	15.6412	0.176255	0.180646	19.8322	0.206409	0.0090618	0.165894	0.349307	7.79882	1.02869	0.702392	7.98544	1.52703	0.295256	0.479867	0.340599	2.7113	60.7882	51.2577	3.46597	2.2	0.1	0.3	1.4
0.0158733	0.069357	19.2584	0.129184	0.130788	24.8316	0.199629	0.146221	0.208371	0.262873	5.17601	0.928476	0.517109	5.21051	1.73923	0.31312	0.484653	0.344908	2.71665	63.7314	54.4765	3.65971	2.6	1	1.8	0
0.0161456	0.0725006	17.3405	0.0731803	0.0825754	25.6637	0.194839	0.0170534	0.204091	0.232591	5.2225	0.970197	0.808053	5.33896	1.4656	0.301029	0.481085	0.33539	2.72156	62.3325	53.5657	3.22226	2.522	0.97	1.746	0
0.0219654	0.119195	19.5741	0.0601993	0.0702751	41.6869	0.198597	0.0401881	0.457704	1.509	4.31771	1.16048	0.444908	5.11245	1.5999	0.309603	0.487694	0.346201	2.75705	81.1317	70.6911	5.1436	2.6	0.2	0.5	1.9
0.0134278	0.130429	18.5198	0.0353552	0.0404908	44.2163	0.199803	0.0193419	0.280026	0.532229	4.26306	0.994975	0.548498	4.77025	1.49419	0.309735	0.480702	0.343027	2.72733	80.4324	71.7694	3.64688	2.522	0.194	0.485	1.843
0.0132705	0.0632482	16.4115	0.0891534	0.0933253	23.7024	0.194253	0.0337214	0.223712	0.393837	5.00122	0.837325	0.488482	5.5019	1.55947	0.293725	0.479436	0.34511	2.73131	59.1526	50.617	3.38151	3.5	2.5	3	0
0.0132705	0.0632482	16.4115	0.0891534	0.0933253	23.7024	0.194253	0.0337214	0.223712	0.393837	5.00122	0.837325	0.488482	5.5019	1.55947	0.293725	0.479436	0.34511	2.73131	59.1526	50.617	3.38151	3	1.5	2.5	0
0.0132705	0.0632482	16.4115	0.0891534	0.0933253	23.7024	0.194253	0.0337214	0.223712	0.393837	5.00122	0.837325	0.488482	5.5019	1.55947	0.293725	0.479436	0.34511	2.73131	59.1526	50.617	3.38151	3.2046	2.289	2.7468	0
0.0132705	0.0632482	16.4115	0.0891534	0.0933253	23.7024	0.194253	0.0337214	0.223712	0.393837	5.00122	0.837325	0.488482	5.5019	1.55947	0.293725	0.479436	0.34511	2.73131	59.1526	50.617	3.38151	2.4	1.4	0	0
0.0167787	0.0910103	27.5917	0.142579	0.124209	35.6419	0.209137	0.0787077	0.242073	1.71199	5.15982	1.49168	0.719265	5.57068	1.76013	0.303499	0.482862	0.358656	2.75723	85.9194	73.9642	5.63516	2	0	0.3	2
0.0167787	0.0910103	27.5917	0.142579	0.124209	35.6419	0.209137	0.0787077	0.242073	1.71199	5.15982	1.49168	0.719265	5.57068	1.76013	0.303499	0.482862	0.358656	2.75723	85.9194	73.9642	5.63516	2.19744	1.28184	0	0
0.0167787	0.0910103	27.5917	0.142579	0.124209	35.6419	0.209137	0.0787077	0.242073	1.71199	5.15982	1.49168	0.719265	5.57068	1.76013	0.303499	0.482862	0.358656	2.75723	85.9194	73.9642	5.63516	2.4	2.4	2.4	2.4
0.0167787	0.0910103	27.5917	0.142579	0.124209	35.6419	0.209137	0.0787077	0.242073	1.71199	5.15982	1.49168	0.719265	5.57068	1.76013	0.303499	0.482862	0.358656	2.75723	85.9194	73.9642	5.63516	2.2	0	0.7	2
0.015384	0.112812	22.7024	0.0399888	0.0415079	40.7985	0.196339	0.0502165	0.187948	1.41381	6.05189	0.784989	0.446537	5.67695	1.44066	0.292393	0.482854	0.338689	2.74772	84.54	75.2298	4.1407	2.7	0.9	1	0
0.015384	0.112812	22.7024	0.0399888	0.0415079	40.7985	0.196339	0.0502165	0.187948	1.41381	6.05189	0.784989	0.446537	5.67695	1.44066	0.292393	0.482854	0.338689	2.74772	84.54	75.2298	4.1407	1.8	1.4	2.3	0
0.015384	0.112812	22.7024	0.0399888	0.0415079	40.7985	0.196339	0.0502165	0.187948	1.41381	6.05189	0.784989	0.446537	5.67695	1.44066	0.292393	0.482854	0.338689	2.74772	84.54	75.2298	4.1407	2.47212	0.82404	0.9156	0
0.015384	0.112812	22.7024	0.0399888	0.0415079	40.7985	0.196339	0.0502165	0.187948	1.41381	6.05189	0.784989	0.446537	5.67695	1.44066	0.292393	0.482854	0.338689	2.74772	84.54	75.2298	4.1407	2.4	2.4	2.4	2.4
0.015384	0.112812	22.7024	0.0399888	0.0415079	40.7985	0.196339	0.0502165	0.187948	1.41381	6.05189	0.784989	0.446537	5.67695	1.44066	0.292393	0.482854	0.338689	2.74772	84.54	75.2298	4.1407	1.5	0	0.9	0
0.015384	0.112812	22.7024	0.0399888	0.0415079	40.7985	0.196339	0.0502165	0.187948	1.41381	6.05189	0.784989	0.446537	5.67695	1.44066	0.292393	0.482854	0.338689	2.74772	84.54	75.2298	4.1407	1.6	0	3	0
0.015384	0.112812	22.7024	0.0399888	0.0415079	40.7985	0.196339	0.0502165	0.187948	1.41381	6.05189	0.784989	0.446537	5.67695	1.44066	0.292393	0.482854	0.338689	2.74772	84.54	75.2298	4.1407	1.3734	0	0.82404	0
0.015384	0.112812	22.7024	0.0399888	0.0415079	40.7985	0.196339	0.0502165	0.187948	1.41381	6.05189	0.784989	0.446537	5.67695	1.44066	0.292393	0.482854	0.338689	2.74772	84.54	75.2298	4.1407	2.4	2.4	2.4	2.4
0.0145413	0.0929786	15.9387	0.0700423	0.0729402	27.3811	0.19361	0.0141899	0.202627	0.224365	4.574	1.22855	0.739324	4.73061	1.5764	0.314606	0.486526	0.340204	2.73325	61.8656	52.6244	3.55765	2		0.7	1
0.0145413	0.0929786	15.9387	0.0700423	0.0729402	27.3811	0.19361	0.0141899	0.202627	0.224365	4.574	1.22855	0.739324	4.73061	1.5764	0.314606	0.486526	0.340204	2.73325	61.8656	52.6244	3.55765	2.8		1.2	0.8
0.0145413	0.0929786	15.9387	0.0700423	0.0729402	27.3811	0.19361	0.0141899	0.202627	0.224365	4.574	1.22855	0.739324	4.73061	1.5764	0.314606	0.486526	0.340204	2.73325	61.8656	52.6244	3.55765	1.8312		0.64092	0.9156
0.0145413	0.0929786	15.9387	0.0700423	0.0729402	27.3811	0.19361	0.0141899	0.202627	0.224365	4.574	1.22855	0.739324	4.73061	1.5764	0.314606	0.486526	0.340204	2.73325	61.8656	52.6244	3.55765	2.4	2.4	2.4	2.4
0.0143264	0.101853	10.9013	0.113165	0.0929438	24.2032	0.194723	0.00747024	0.273883	1.34475	4.53633	1.02827	0.803538	4.67622	1.54018	0.295849	0.482565	0.341936	2.71756	54.5109	44.3171	4.50719	3.5	1.2	0.8	1.6
0.0143264	0.101853	10.9013	0.113165	0.0929438	24.2032	0.194723	0.00747024	0.273883	1.34475	4.53633	1.02827	0.803538	4.67622	1.54018	0.295849	0.482565	0.341936	2.71756	54.5109	44.3171	4.50719	3.7	1.9	2.3	1.3
0.0143264	0.101853	10.9013	0.113165	0.0929438	24.2032	0.194723	0.00747024	0.273883	1.34475	4.53633	1.02827	0.803538	4.67622	1.54018	0.295849	0.482565	0.341936	2.71756	54.5109	44.3171	4.50719	3.2046	1.09872	0.73248	1.46496
0.0143264	0.101853	10.9013	0.113165	0.0929438	24.2032	0.194723	0.00747024	0.273883	1.34475	4.53633	1.02827	0.803538	4.67622	1.54018	0.295849	0.482565	0.341936	2.71756	54.5109	44.3171	4.50719	2.4	2.4	2.4	2.4
0.0139869	0.0993022	18.4724	0.133655	0.118945	30.3122	0.20376	0.0183623	0.490895	0.595578	4.59771	1.16253	0.528152	5.1209	1.66122	0.300793	0.484957	0.352045	2.72585	68.5589	58.5032	4.33193	3.7	1.9	2.3	1.3
0.0139869	0.0993022	18.4724	0.133655	0.118945	30.3122	0.20376	0.0183623	0.490895	0.595578	4.59771	1.16253	0.528152	5.1209	1.66122	0.300793	0.484957	0.352045	2.72585	68.5589	58.5032	4.33193	3.589	1.843	2.231	1.261

0.0139869	0.0993022	18.4724	0.133655	0.118945	30.3122	0.20376	0.0183623	0.490895	0.595578	4.59771	1.16253	0.528152	5.1209	1.66122	0.300793	0.484957	0.352045	2.72585	68.5589	58.5032	4.33193	3.8295	1.9665	2.3805	1.3455
0.0139869	0.0993022	18.4724	0.133655	0.118945	30.3122	0.20376	0.0183623	0.490895	0.595578	4.59771	1.16253	0.528152	5.1209	1.66122	0.300793	0.484957	0.352045	2.72585	68.5589	58.5032	4.33193	3.71462	1.90751	2.30908	1.30514
0.0126175	0.0334004	6.68082	0.121074	0.0909457	9.16253	0.199874	0.0232676	0.302016	1.39054	3.39796	0.822817	0.394555	4.43712	1.46121	0.308314	0.480219	0.342208	2.72207	33.139	23.6784	4.2674	2.3	0.8	0.8	1.2
0.0126175	0.0334004	6.68082	0.121074	0.0909457	9.16253	0.199874	0.0232676	0.302016	1.39054	3.39796	0.822817	0.394555	4.43712	1.46121	0.308314	0.480219	0.342208	2.72207	33.139	23.6784	4.2674	2.231	0.776	0.776	1.164
0.0126175	0.0334004	6.68082	0.121074	0.0909457	9.16253	0.199874	0.0232676	0.302016	1.39054	3.39796	0.822817	0.394555	4.43712	1.46121	0.308314	0.480219	0.342208	2.72207	33.139	23.6784	4.2674	2.3805	0.828	0.828	1.242
0.0126175	0.0334004	6.68082	0.121074	0.0909457	9.16253	0.199874	0.0232676	0.302016	1.39054	3.39796	0.822817	0.394555	4.43712	1.46121	0.308314	0.480219	0.342208	2.72207	33.139	23.6784	4.2674	2.30908	0.80316	0.80316	1.20474
0.012417	0.0346081	11.996	0.0765618	0.0774414	21.3072	0.203413	0.0158344	0.290365	0.940931	4.32647	0.979377	0.829678	4.62394	1.5543	0.314391	0.484804	0.341174	2.7192	52.1666	42.2536	4.06572				
0.012417	0.0346081	11.996	0.0765618	0.0774414	21.3072	0.203413	0.0158344	0.290365	0.940931	4.32647	0.979377	0.829678	4.62394	1.5543	0.314391	0.484804	0.341174	2.7192	52.1666	42.2536	4.06572				
0.012417	0.0346081	11.996	0.0765618	0.0774414	21.3072	0.203413	0.0158344	0.290365	0.940931	4.32647	0.979377	0.829678	4.62394	1.5543	0.314391	0.484804	0.341174	2.7192	52.1666	42.2536	4.06572				
0.012417	0.0346081	11.996	0.0765618	0.0774414	21.3072	0.203413	0.0158344	0.290365	0.940931	4.32647	0.979377	0.829678	4.62394	1.5543	0.314391	0.484804	0.341174	2.7192	52.1666	42.2536	4.06572				
0.0150333	0.039748	10.1741	0.0443408	0.0393278	16.1893	0.198228	0.0186895	0.444631	1.32436	3.91438	0.861265	0.665743	4.45739	1.40841	0.301263	0.483663	0.341871	2.71356	44.3883	34.7351	4.36968	1	1.2	1.5	1.8
0.0150333	0.039748	10.1741	0.0443408	0.0393278	16.1893	0.198228	0.0186895	0.444631	1.32436	3.91438	0.861265	0.665743	4.45739	1.40841	0.301263	0.483663	0.341871	2.71356	44.3883	34.7351	4.36968	0.97	1.164	1.455	1.746
0.0150333	0.039748	10.1741	0.0443408	0.0393278	16.1893	0.198228	0.0186895	0.444631	1.32436	3.91438	0.861265	0.665743	4.45739	1.40841	0.301263	0.483663	0.341871	2.71356	44.3883	34.7351	4.36968	1.035	1.242	1.5525	1.863
0.0150333	0.039748	10.1741	0.0443408	0.0393278	16.1893	0.198228	0.0186895	0.444631	1.32436	3.91438	0.861265	0.665743	4.45739	1.40841	0.301263	0.483663	0.341871	2.71356	44.3883	34.7351	4.36968	1.00395	1.20474	1.50593	1.80711
0.0100825	0.03021	11.6282	0.0497461	0.0434266	11.5468	0.193161	0.0208963	0.225476		3.8303	0.836773	0.651525	4.48279	1.48074	0.298739	0.475203	0.332951	2.71419	39.5441	31.4881	2.72241	3	2.2	2.8	0
0.0100825	0.03021	11.6282	0.0497461	0.0434266	11.5468	0.193161	0.0208963	0.225476		3.8303	0.836773	0.651525	4.48279	1.48074	0.298739	0.475203	0.332951	2.71419	39.5441	31.4881	2.72241	2.5	0	2.5	0
0.0100825	0.03021	11.6282	0.0497461	0.0434266	11.5468	0.193161	0.0208963	0.225476		3.8303	0.836773	0.651525	4.48279	1.48074	0.298739	0.475203	0.332951	2.71419	39.5441	31.4881	2.72241	2.7468	2.01432	2.56368	0
0.0100825	0.03021	11.6282	0.0497461	0.0434266	11.5468	0.193161	0.0208963	0.225476		3.8303	0.836773	0.651525	4.48279	1.48074	0.298739	0.475203	0.332951	2.71419	39.5441	31.4881	2.72241	2.4	2.4	2.4	2.4
0.00883132	0.040395	17.3724	0.0499878	0.045183	20.2353	0.196353	0.00525099	0.327899	0.346811	4.31417	0.966938	1.13451	4.44588	1.41119	0.29621	0.48639	0.349966	2.70211	55.5658	46.3678	3.35163	2.5	1	1	0
0.00883132	0.040395	17.3724	0.0499878	0.045183	20.2353	0.196353	0.00525099	0.327899	0.346811	4.31417	0.966938	1.13451	4.44588	1.41119	0.29621	0.48639	0.349966	2.70211	55.5658	46.3678	3.35163	2	2.6	0.5	0
0.00883132	0.040395	17.3724	0.0499878	0.045183	20.2353	0.196353	0.00525099	0.327899	0.346811	4.31417	0.966938	1.13451	4.44588	1.41119	0.29621	0.48639	0.349966	2.70211	55.5658	46.3678	3.35163	2.289	0.9156	0.9156	0
0.00883132	0.040395	17.3724	0.0499878	0.045183	20.2353	0.196353	0.00525099	0.327899	0.346811	4.31417	0.966938	1.13451	4.44588	1.41119	0.29621	0.48639	0.349966	2.70211	55.5658	46.3678	3.35163	2.4	2.4	2.4	2.4
0.013908	0.0775452	18.7463	0.0577963	0.0618147	31.7658	0.204347	0.00191761	0.370265	0.754152	4.91866	0.887096	0.437468	4.91299	1.50448	0.313438	0.479673	0.326825	2.69767	69.526	60.3437	3.81079	2.3	1	2	0
0.013908	0.0775452	18.7463	0.0577963	0.0618147	31.7658	0.204347	0.00191761	0.370265	0.754152	4.91866	0.887096	0.437468	4.91299	1.50448	0.313438	0.479673	0.326825	2.69767	69.526	60.3437	3.81079	2.10588	0.9156	1.8312	0
0.013908	0.0775452	18.7463	0.0577963	0.0618147	31.7658	0.204347	0.00191761	0.370265	0.754152	4.91866	0.887096	0.437468	4.91299	1.50448	0.313438	0.479673	0.326825	2.69767	69.526	60.3437	3.81079	2.4	2.4	2.4	2.4
0.013908	0.0775452	18.7463	0.0577963	0.0618147	31.7658	0.204347	0.00191761	0.370265	0.754152	4.91866	0.887096	0.437468	4.91299	1.50448	0.313438	0.479673	0.326825	2.69767	69.526	60.3437	3.81079	2.19744	2.19744	2.19744	2.19744
0.0129312	0.0321868	9.05235	0.0451934	0.0435522	16.6969	0.204241	0.0473545	0.198082	0.150219	4.05828	1.20013	0.769078	4.47329	1.44885	0.308447	0.48493	0.331311	2.71731	43.0199	34.2809	3.38922				
0.0106611	0.0290454	14.9167	0.0447885	0.0422892	22.0998	0.205094	0.0111883	0.358326	0.860145	4.78168	0.810096	0.729195	4.98883	1.50303	0.326416	0.48413	0.345037	2.70168	55.9765	46.787	3.82646				
0.0223341	0.0365751	13.9084	0.0497649	0.047514	28.9292	0.206114	0.00777528	0.27427	2.0645	5.01529	0.879781	0.827087	5.39054	1.94162	0.345457	0.490273	0.353712	2.70558	64.1983	53.2435	5.45533	2	0.7	0	0
0.0120064	0.0309091	9.96076	0.0864953	0.0768193	11.9666	0.200989	0.13384	0.337848	0.454778	3.69405	1.01663	0.54607	4.36062	1.42464	0.305809	0.486317	0.338841	2.70079	39.2547	29.9821	3.69956	1.9	1	1	0
0.0120064	0.0309091	9.96076	0.0864953	0.0768193	11.9666	0.200989	0.13384	0.337848	0.454778	3.69405	1.01663	0.54607	4.36062	1.42464	0.305809	0.486317	0.338841	2.70079	39.2547	29.9821	3.69956	1.7	0	0	1.3
0.0120064	0.0309091	9.96076	0.0864953	0.0768193	11.9666	0.200989	0.13384	0.337848	0.454778	3.69405	1.01663	0.54607	4.36062	1.42464	0.305809	0.486317	0.338841	2.70079	39.2547	29.9821	3.69956	1.89652	0	0	1.45028
0.0120064	0.0309091	9.96076	0.0864953	0.0768193	11.9666	0.200989	0.13384	0.337848	0.454778	3.69405	1.01663	0.54607	4.36062	1.42464	0.305809	0.486317	0.338841	2.70079	39.2547	29.9821	3.69956	2.11964	1.1156	1.1156	0
0.0156894	0.03214	15.6387	0.0771274	0.0715298	18.4916	0.228186	0.0281528	0.211862	2.74955	4.86306	0.919879	0.649266	5.31499	1.86195	0.324151	0.4864	0.345609	2.73922	56.5727	44.3083	6.02091	1.6	0	0	1.5
0.0156894	0.03214	15.6387	0.0771274	0.0715298	18.4916	0.228186	0.0281528	0.211862	2.74955	4.86306	0.919879	0.649266	5.31499	1.86195	0.324151	0.4864	0.345609	2.73922	56.5727	44.3083	6.02091	1.46496	0	0	1.3734
0.0156894	0.03214	15.6387	0.0771274	0.0715298	18.4916	0.228186	0.0281528	0.211862	2.74955	4.86306	0.919879	0.649266	5.31499	1.86195	0.324151	0.4864	0.345609	2.73922	56.5727	44.3083	6.02091	1.63431	0	0	1.53217
0.0156894	0.03214	15.6387	0.0771274	0.0715298	18.4916	0.228186	0.0281528	0.211862	2.74955	4.86306	0.919879	0.649266	5.31499	1.86195	0.324151	0.4864	0.345609	2.73922	56.5727	44.3083	6.02091	1.78496	0	0	1.6734
0.0108668	0.0277804	5.80714	0.0623292	0.0752774	7.30591	0.196973	0.0229968	0.150325	0.52114	3.25487	0.848386	0.610665	4.37552	1.50187	0.306999	0.478825	0.334944	2.70776	29.8811	20.7434	3.31452	2.9	0.8	0.8	1.9
0.0108668	0.0277804	5.80714	0.0623292	0.0752774	7.30591	0.196973	0.0229968	0.150325	0.52114	3.25487	0.848386	0.610665	4.37552	1.50187	0.306999	0.478825	0.334944	2.70776	29.8811	20.7434	3.31452				
0.0108668	0.0277804	5.80714	0.0623292	0.0752774	7.30591	0.196973	0.0229968	0.150325	0.52114	3.25487	0.848386	0.610665	4.37552	1.50187	0.306999	0.478825	0.334944	2.70776	29.8811	20.7434	3.31452				
0.0108668	0.0277804	5.80714	0.0623292	0.0752774	7.30591	0.196973	0.0229968	0.150325	0.52114	3.25487	0.848386	0.610665	4.37552	1.50187	0.306999	0.478825	0.334944	2.70776	29.8811	20.7434	3.31452				
0.0108668	0.0277804	5.80714	0.0623292	0.0752774	7.30591	0.196973	0.0229968	0.150325	0.52114	3.25487	0.848386	0.610665	4.37552	1.50187	0.306999	0.478825	0.334944	2.70776	29.8811	20.7434	3.31452				
0.0103814	0.0296295	6.20932	0.0793248	0.0717872	8.58253	0.201365	0.00907258	0.32861	1.7621	3.46789	0.823565	0.751353	4.25614	1.57086	0.311944	0.481758	0.3253	2.70455	32.8152	22.5159	4.64651	2	1.5	2.2	2.1
0.0103814	0.0296295	6.20932	0.0793248	0.0717872	8.58253	0.201365	0.00907258	0.32861	1.7621	3.46789	0.823565	0.751353	4.25614	1.57086	0.311944	0.481758	0.3253	2.70455	32.8152	22.5159	4.64651	1	0	1.5	1.1
0.0103814	0.0296295	6.20932	0.0793248	0.0717872	8.58253	0.201365	0.00907258	0.32861	1.7621	3.46789	0.823565	0.751353	4.25614	1.57086	0.311944	0.481758	0.3253	2.70455	32.8152	22.5159	4.64651	1.1156	0	1.6734	1.22716
0.0103814	0.0296295	6.20932	0.0793248	0.0717872	8.58253	0.201365	0.00907258	0.32861	1.7621	3.46789	0.823565	0.751353	4.25614	1.57086	0.311944	0.481758	0.3253	2.70455	32.8152	22.5159	4.64651	2.2312	1.6734	2.45432	2.34276
0.0163719	0.0400194	9.15185	0.0806118	0.0792201	18.7199	0.205512	0.025016	0.190429	0.675634	4.38339	2.48498	4.04884	4.61522	1.91277	0.336305	0.491668	0.447302	2.82221	51.6277	36.8704	5.56181				
0.0163719	0.0400194	9.15185	0.0806118	0.0792201	18.7199	0.205512	0.025016	0.190429	0.675634	4.38339	2.48498	4.04884	4.61522	1.91277	0.336305	0.491668	0.447302	2.82221	51.6277	36.8704	5.56181				
0.0163719	0.0400194	9.15185	0.0806118	0.0792201	18.7199	0.205512	0.025016	0.190429	0.675634	4.38339	2.48498	4.04884	4.61522	1.91277	0.336305	0.491668	0.447302	2.82221	51.6277	36.8704	5.56181				
0.0163719	0.0400194	9.15185	0.0806118	0.0792201	18.7199	0.205512	0.025016	0.190429	0.675634	4.38339	2.48498	4.04884	4.61522	1.91277	0.336305	0.491668	0.447302	2.82221	51.6277	36.8704	5.56181				
0.0169922	0.143253	9.42756	0.0660557	0.0490498	24.677	0.198422	0.068119	0.158771	1.14191	4.70391	1.00395	0.643887	6.21939	2.40775	0.38326	0.490868	0.458386	2.80617	56.1925	45.0278	5.34689	3.8	2	2	0
0.0169922	0.143253	9.42756	0.0660557	0.0490498	24.677	0.198422	0.068119	0.158771	1.14191	4.70391	1.00395	0.643887	6.21939	2.40775	0.38326	0.490868	0.458386	2.80617	56.1925	45.0278	5.34689	4	1	0	0
0.0169922	0.143253	9.42756	0.0660557	0.0490498	24.677	0.198422	0.068119	0.158771	1.14191	4.70391	1.00395	0.643887	6.21939	2.40775	0.38326	0.490868	0.458386	2.80617	56.1925	45.0278	5.34689	3	1.4	1.6	0
0.0169922	0.143253	9.42756	0.0660557	0.0490498	24.677	0.198422	0.068119	0.158771	1.14191	4.70391	1.00395	0.643887	6.21939	2.40775	0.38326	0.490868	0.458386	2.80617	56.1925	45.0278	5.34689	1	1.5	2.5	0
0.0246506	0.206797	10.7796	0.0650852	0.0386082	36.0508	0.194518	0.0965459	0.202078	0.850787	4.58415	0.984515	0.664321	4.48132	1.72383	0.329832	0.497776	0.434655	2.76984	66.2563	55.8959	4.57185	5.8	1	2	0
0.0246506	0.206797	10.7796	0.0650852	0.0386082	36.0508	0.194518	0.0965459	0.202078	0.850787	4.58415	0.984515	0.664321	4.48132	1.72383	0.329832	0.497776	0.434655	2.76984	66.2563	55.8959	4.57185	5.626	0.97	1.94	0
0.0246506	0.206797	10.7796	0.0650852	0.0386082	36.0508	0.194518	0.0965459	0.202078	0.850787	4.58415	0.984515	0.664321	4.48132	1.72383	0.329832	0.497776	0.434655	2.76984	66.2563	55.8959	4.57185	6.003	1.035	2.07	0
0.0246506	0.206797	10.7796	0.0650852	0.0386082	36.0508	0.194518	0.0965459	0.202078	0.850787	4.58415	0.984515	0.664321	4.48132	1.72383	0.329832	0.497776	0.434655	2.76984	66.2563	55.8959	4.57185	5.82291	1.00395	2.0079	0
0.0246506	0.206797	10.7796	0.0650852	0.0386082	36.0508	0.194518	0.0965459	0.202078	0.850787	4.58415	0.984515	0.664321	4.48132	1.72383	0.329832	0.497776	0.434655	2.76984	66.2563	55.8959	4.57185	6.21311	1.07123	2.14245	0
0.0144126	0.0945133	10.1127	0.0288607	0.0288006	27.3636	0.191902	0.0785022	0.215936	0.845429	4.66623	0.966743	0.748575	4.40584	1.92084	0.338113	0.488974	0.456343	2.80189	57.0204	46.5483	4.80973	3.3	2	2.5	0
0.0144126	0.0945133	10.1127	0.0288607	0.0288006	27.3636	0.191902	0.0785022	0.215936	0.845429	4.66623	0.966743	0.748575	4.40584	1.92084	0.338113	0.488974	0.456343	2.80189	57.0204	46.5483	4.80973	5	2.9	2.5	0
0.0144126	0.0945133	10.1127	0.0288607	0.0288006	27.3636	0.191902	0.0785022	0.215936	0.845429	4.66623	0.966743	0.748575	4.40584	1.92084	0.338113	0.488974	0.456343	2.80189	57.0204	46.5483	4.80973				
0.0144126	0.0945133	10.1127	0.0288607	0.0288006	27.3636	0.191902	0.0785022	0.215936	0.845429	4.66623	0.966743	0.748575	4.40584	1.92084	0.338113	0.488974	0.456343	2.80189	57.0204	46.5483	4.80973	2.5	3	3	0
0.0126597	0.114216	11.5356	0.04965	0.0414557	35.4099	0.192825	0.0481548	0.163766	0.912406	4.46557	0.885533	0.634043	4.89645	1.71011	0.335017	0.487685	0.437274	2.74496	66.1359	56.3075	4.21007	4.5	2.5	2.5	0
0.0126597	0.114216	11.5356	0.04965	0.0414557	35.4099	0.192825	0.0481548	0.163766	0.912406	4.46557	0.885533	0.634043	4.89645	1.71011	0.335017	0.487685	0.437274	2.74496	66.1359	56.3075	4.21007	2.5	0	0	0
0.0126597	0.114216	11.5356	0.04965	0.0414557	35.4099	0.192825	0.0481548	0.163766	0.912406	4.46557	0.885533	0.634043	4.89645	1.71011	0.335017	0.487685	0.437274	2.74496	66.1359	56.3075	4.21007	4.2	3.2	0	0
0.0126597	0.114216	11.5356	0.04965	0.0414557	35.4099	0.192825	0.0481548	0.163766	0.912406	4.46557	0.885533	0.634043	4.89645	1.71011	0.335017	0.487685	0.437274	2.74496	66.1359	56.3075	4.21007				
0.0219516	0.130999	10.4003	0.042948	0.0354501	24.4919	0.188284	0.0899655	0.166888	1.1719	4.74251	0.834618	0.495685	4.51465	1.84925	0.358107	0.48205	0.41419	2.72418	54.3581	44.1493	4.88266	3.5	2	2	0
0.0219516	0.130999	10.4003	0.042948	0.0354501	24.4919	0.188284	0.0899655	0.166888	1.1719	4.74251	0.834618	0.495685	4.51465	1.84925	0.358107	0.48205	0.41419	2.72418	54.3581	44.1493	4.88266	5.1	1.2	3.3	0
0.0219516	0.130999	10.4003	0.042948	0.0354501	24.4919	0.188284	0.0899655	0.166888	1.1719	4.74251	0.834618	0.495685	4.51465	1.84925	0.358107	0.48205	0.41419	2.72418	54.3581	44.1493	4.88266	3	2.4	4	0
0.0219516	0.130999	10.4003	0.042948	0.0354501	24.4919	0.188284	0.0899655	0.166888	1.1719	4.74251	0.834618	0.495685	4.51465	1.84925	0.358107	0.48205	0.41419	2.72418	54.3581	44.1493	4.88266	3.5	3.5	3.5	0
0.0106638	0.125985	8.82027	0.0916847	0.0518124	11.574	0.19425	0.201678	0.334358	1.08594	12.4836	0.885345	0.754879	13.744	3.16668	0.410662	0.490513	0.465558	2.84859	60.1256	46.6219	6.79695	3.5	1	2.5	0
0.0106638	0.125985	8.82027	0.0916847	0.0518124	11.574	0.19425	0.201678	0.334358	1.08594	12.4836	0.885345	0.754879	13.744	3.16668	0.410662	0.490513	0.465558	2.84859	60.1256	46.6219	6.79695	3.5	1.1	1.9	0
0.0106638	0.125985	8.82027	0.0916847	0.0518124	11.574	0.19425	0.201678	0.334358	1.08594	12.4836	0.885345	0.754879	13.744	3.16668	0.410662	0.490513	0.465558	2.84859	60.1256	46.6219	6.79695	2.8	1	2	0
0.0106638	0.125985	8.82027	0.0916847	0.0518124	11.574	0.19425	0.201678	0.334358	1.08594	12.4836	0.885345	0.754879	13.744	3.16668	0.410662	0.490513	0.465558	2.84859	60.1256	46.6219	6.79695	2.5	1	3	0
0.0183495	0.206596	12.5629	0.056592	0.0454321	36.5393	0.188675	0.0836056	0.20388	0.593267	6.91457	0.96354	0.295389	10.1074	1.7711	0.307987	0.484646	0.368002	2.70783	75.8591	66.1242	4.33192	3.5	2	2.5	0
0.0183495	0.206596	12.5629	0.056592	0.0454321	36.5393	0.188675	0.0836056	0.20388	0.593267	6.91457	0.96354	0.295389	10.1074	1.7711	0.307987	0.484646	0.368002	2.70783	75.8591	66.1242	4.33192	2	1.2	2.6	0
0.0183495	0.206596	12.5629	0.056592	0.0454321	36.5393	0.188675	0.0836056	0.20388	0.593267	6.91457	0.96354	0.295389	10.1074	1.7711	0.307987	0.484646	0.368002	2.70783	75.8591	66.1242	4.33192	3.7	2.1	3.5	0
0.0183495	0.206596	12.5629	0.056592	0.0454321	36.5393	0.188675	0.0836056	0.20388	0.593267	6.91457	0.96354	0.295389	10.1074	1.7711	0.307987	0.484646	0.368002	2.70783	75.8591	66.1242	4.33192				
0.0278494	0.216444	13.653	0.174888	0.158468	34.0706	0.202279	0.0798574	0.19574	0.883367	12.6537	1.01352	1.10312	14.5596	1.96078	0.341715	0.485955	0.453794	2.85149	87.9709	74.9369	6.28721	4.5	1.7	1.8	0
0.0135174	0.149881	8.82121	0.0514868	0.0397313	35.1932	0.191111	0.0437801	0.217388	0.456031	4.47765	0.970333	0.512958	4.49739	1.61742	0.31157	0.499913	0.423829	2.79558	62.5692	52.9895	3.93613	2.3	0	0	0
0.0197942	0.146843	8.9465	0.171255	0.104986	9.51854	0.200574	0.0988059	0.330325	2.02453	19.624	0.933895	0.976868	15.1882	1.6427	0.368662	0.487191	0.457017	2.8597	66.9523	53.2772	6.63456				
0.0296918	0.12215	5.437	0.204126	0.115123	4.893	0.201317	0.0743874	0.296001	0.604249	27.4058	0.979171	1.02791	16.1368	1.95704	0.363547	0.510835	0.462242	2.82667	67.0596	53.8726	6.09267	3	1.2	2	0
0.0223418	0.156341	17.2473	0.0483469	0.0380906	50.0908	0.195558	0.109595	0.135846	4.77517	6.2529	0.927965	0.920713	4.73121	1.723	0.341575	0.481465	0.424362	2.75971	92.6692	78.3222	8.47796				
0.0225994	0.152414	13.2779	0.0390256	0.038143	34.4177	0.194703	0.0949746	0.155202	3.23355	5.22598	0.856571	0.60576	4.54203	1.67203	0.316751	0.483925	0.419989	2.74392	69.6521	57.4636	6.75308				
0.023043	0.139425	9.67298	0.0731563	0.0570222	29.5733	0.192735	0.0805844	0.226387	1.91366	4.58768	0.858444	0.982168	4.46229	1.69339	0.320045	0.483457	0.422223	2.74109	59.7261	48.2963	5.59153	3.5	2.5	2.5	0
0.023043	0.139425	9.67298	0.0731563	0.0570222	29.5733	0.192735	0.0805844	0.226387	1.91366	4.58768	0.858444	0.982168	4.46229	1.69339	0.320045	0.483457	0.422223	2.74109	59.7261	48.2963	5.59153	4.9	2.4	3.6	0
0.023043	0.139425	9.67298	0.0731563	0.0570222	29.5733	0.192735	0.0805844	0.226387	1.91366	4.58768	0.858444	0.982168	4.46229	1.69339	0.320045	0.483457	0.422223	2.74109	59.7261	48.2963	5.59153	3	2	2	0
0.023043	0.139425	9.67298	0.0731563	0.0570222	29.5733	0.192735	0.0805844	0.226387	1.91366	4.58768	0.858444	0.982168	4.46229	1.69339	0.320045	0.483457	0.422223	2.74109	59.7261	48.2963	5.59153	2	2.5	2.5	0
0.0254354	0.157638	14.7321	0.0369344	0.0393213	44.3037	0.192455	0.0942901	0.197526	1.81804	6.95452	0.912786	0.654846	6.96163	1.76283	0.349669	0.480751	0.438813	2.74437	84.1734	72.9519	5.63112	3.5	1.8	2.5	0
0.0254354	0.157638	14.7321	0.0369344	0.0393213	44.3037	0.192455	0.0942901	0.197526	1.81804	6.95452	0.912786	0.654846	6.96163	1.76283	0.349669	0.480751	0.438813	2.74437	84.1734	72.9519	5.63112				
0.0254354	0.157638	14.7321	0.0369344	0.0393213	44.3037	0.192455	0.0942901	0.197526	1.81804	6.95452	0.912786	0.654846	6.96163	1.76283	0.349669	0.480751	0.438813	2.74437	84.1734	72.9519	5.63112				
0.0254354	0.157638	14.7321	0.0369344	0.0393213	44.3037	0.192455	0.0942901	0.197526	1.81804	6.95452	0.912786	0.654846	6.96163	1.76283	0.349669	0.480751	0.438813	2.74437	84.1734	72.9519	5.63112				
0.024202	0.160116	7.88544	0.0385561	0.0271292	26.7034	0.192748	0.0542901	0.209459	1.00883	4.12005	1.26239	0.727806	4.48934	2.13634	0.354219	0.513115	0.454089	2.84476	54.3215	43.1982	5.24171	3.5	2.5	3.5	0
0.024202	0.160116	7.88544	0.0385561	0.0271292	26.7034	0.192748	0.0542901	0.209459	1.00883	4.12005	1.26239	0.727806	4.48934	2.13634	0.354219	0.513115	0.454089	2.84476	54.3215	43.1982	5.24171	2.7	2.4	4.9	0
0.024202	0.160116	7.88544	0.0385561	0.0271292	26.7034	0.192748	0.0542901	0.209459	1.00883	4.12005	1.26239	0.727806	4.48934	2.13634	0.354219	0.513115	0.454089	2.84476	54.3215	43.1982	5.24171	3	2.2	3	0
0.024202	0.160116	7.88544	0.0385561	0.0271292	26.7034	0.192748	0.0542901	0.209459	1.00883	4.12005	1.26239	0.727806	4.48934	2.13634	0.354219	0.513115	0.454089	2.84476	54.3215	43.1982	5.24171	2.5	4	3.5	0
0.0366873	0.151707	18.2098	0.0518983	0.0463279	49.3784	0.193784	0.114558	0.162427	3.25082	6.86272	0.887526	0.973185	5.63776	1.6978	0.330715	0.481705	0.422765	2.76938	92.8796	80.0887	6.89959	4	1.5	1.5	0
0.0366873	0.151707	18.2098	0.0518983	0.0463279	49.3784	0.193784	0.114558	0.162427	3.25082	6.86272	0.887526	0.973185	5.63776	1.6978	0.330715	0.481705	0.422765	2.76938	92.8796	80.0887	6.89959				
0.0366873	0.151707	18.2098	0.0518983	0.0463279	49.3784	0.193784	0.114558	0.162427	3.25082	6.86272	0.887526	0.973185	5.63776	1.6978	0.330715	0.481705	0.422765	2.76938	92.8796	80.0887	6.89959				
0.0366873	0.151707	18.2098	0.0518983	0.0463279	49.3784	0.193784	0.114558	0.162427	3.25082	6.86272	0.887526	0.973185	5.63776	1.6978	0.330715	0.481705	0.422765	2.76938	92.8796	80.0887	6.89959				
0.0259753	0.171322	18.2016	0.0486686	0.0414103	40.9874	0.18977	0.120617	0.264423	2.60008	9.13196	0.823037	0.844351	9.33727	1.66459	0.342166	0.479959	0.431971	2.74764	89.8285	77.6583	6.37659	2.5	1.5	1.5	0
0.0259753	0.171322	18.2016	0.0486686	0.0414103	40.9874	0.18977	0.120617	0.264423	2.60008	9.13196	0.823037	0.844351	9.33727	1.66459	0.342166	0.479959	0.431971	2.74764	89.8285	77.6583	6.37659	2.789	1.6734	1.6734	0
0.0259753	0.171322	18.2016	0.0486686	0.0414103	40.9874	0.18977	0.120617	0.264423	2.60008	9.13196	0.823037	0.844351	9.33727	1.66459	0.342166	0.479959	0.431971	2.74764	89.8285	77.6583	6.37659	3.11141	1.86685	1.86685	0
0.0259753	0.171322	18.2016	0.0486686	0.0414103	40.9874	0.18977	0.120617	0.264423	2.60008	9.13196	0.823037	0.844351	9.33727	1.66459	0.342166	0.479959	0.431971	2.74764	89.8285	77.6583	6.37659	2.84881	1.70928	1.70928	0
0.0133487	0.151893	8.06136	0.112841	0.0931842	24.4742	0.196359	0.0503974	0.174362	0.835677	8.8573	0.812018	0.425717	14.1997	1.72245	0.339419	0.482195	0.42668	2.80879	66.1505	55.5926	4.93718	3.5	2.5	3	0
0.0129483	0.147336	7.81952	0.109456	0.0903887	23.74	0.190468	0.0488855	0.169131	0.810607	8.59158	0.787657	0.412945	13.7738	1.67077	0.329237	0.467729	0.413879	2.72452	64.166	53.9248	4.78907	2.3	0.4	0.3	0

0.0138159	0.157209	8.34351	0.116791	0.0964456	25.3308	0.203232	0.0521613	0.180464	0.864926	9.1673	0.840438	0.440617	14.6967	1.78273	0.351299	0.499072	0.441613	2.9071	68.4658	57.5384	5.10998	3.5	1	2	0
0.0134015	0.152493	8.09321	0.113287	0.0935523	24.5709	0.197135	0.0505965	0.17505	0.838978	8.89228	0.815225	0.427398	14.2558	1.72925	0.34076	0.4841	0.428365	2.81988	66.4118	55.8122	4.95668				
0.0129994	0.147918	7.85041	0.109888	0.0907457	23.8338	0.191221	0.0490786	0.169799	0.813809	8.62551	0.790768	0.414577	13.8282	1.67737	0.330537	0.469577	0.415514	2.73529	64.4195	54.1378	4.80798				
0.0138705	0.15783	8.37647	0.117252	0.0968266	25.4309	0.204034	0.0523674	0.181177	0.868342	9.20351	0.843758	0.442357	14.7548	1.78978	0.352686	0.501043	0.443358	2.91858	68.7362	57.7656	5.13017				
0.0134544	0.153095	8.12517	0.113734	0.0939218	24.6679	0.197913	0.0507964	0.175742	0.842292	8.92741	0.818445	0.429087	14.3121	1.73608	0.342106	0.486012	0.430057	2.83102	66.6742	56.0327	4.97626				
0.0144241	0.0807199	10.2374	0.0831867	0.0563322	17.5788	0.190943	0.0467682	0.177287	0.542466	3.79745	1.8923	0.452884	4.6847	1.45428	0.315403	0.482038	0.335816	2.72991	46.0568	36.2983	4.4479	2.1	1	1.6	0
0.0144241	0.0807199	10.2374	0.0831867	0.0563322	17.5788	0.190943	0.0467682	0.177287	0.542466	3.79745	1.8923	0.452884	4.6847	1.45428	0.315403	0.482038	0.335816	2.72991	46.0568	36.2983	4.4479	2.3	0.5	0	0
0.0144241	0.0807199	10.2374	0.0831867	0.0563322	17.5788	0.190943	0.0467682	0.177287	0.542466	3.79745	1.8923	0.452884	4.6847	1.45428	0.315403	0.482038	0.335816	2.72991	46.0568	36.2983	4.4479	2.56588	0.5578	0	0
0.0144241	0.0807199	10.2374	0.0831867	0.0563322	17.5788	0.190943	0.0467682	0.177287	0.542466	3.79745	1.8923	0.452884	4.6847	1.45428	0.315403	0.482038	0.335816	2.72991	46.0568	36.2983	4.4479	2.34932	0.510722	0	0
0.0210331	0.0843532	8.93021	0.120076	0.0869628	19.4599	0.192436	0.0570152	0.159382	0.142247	4.83852	0.910719	0.70536	6.28658	1.46314	0.30383	0.48524	0.341566	2.7992	48.357	39.5152	3.18796	3	1.5	2	0
0.0210331	0.0843532	8.93021	0.120076	0.0869628	19.4599	0.192436	0.0570152	0.159382	0.142247	4.83852	0.910719	0.70536	6.28658	1.46314	0.30383	0.48524	0.341566	2.7992	48.357	39.5152	3.18796	3.3468	1.6734	2.2312	0
0.0210331	0.0843532	8.93021	0.120076	0.0869628	19.4599	0.192436	0.0570152	0.159382	0.142247	4.83852	0.910719	0.70536	6.28658	1.46314	0.30383	0.48524	0.341566	2.7992	48.357	39.5152	3.18796	3.06433	1.53217	2.04289	0
0.0210331	0.0843532	8.93021	0.120076	0.0869628	19.4599	0.192436	0.0570152	0.159382	0.142247	4.83852	0.910719	0.70536	6.28658	1.46314	0.30383	0.48524	0.341566	2.7992	48.357	39.5152	3.18796				
0.0152772	0.0982946	11.32	0.0318484	0.0255398	21.3486	0.189677	0.0326623	0.163953	0.515002	4.61827	0.995103	0.88605	5.38095	1.47297	0.314138	0.48405	0.340227	2.82893	51.6589	42.6677	3.58297	3.5	2	2	0
0.0152772	0.0982946	11.32	0.0318484	0.0255398	21.3486	0.189677	0.0326623	0.163953	0.515002	4.61827	0.995103	0.88605	5.38095	1.47297	0.314138	0.48405	0.340227	2.82893	51.6589	42.6677	3.58297	3.395	1.94	1.94	0
0.0152772	0.0982946	11.32	0.0318484	0.0255398	21.3486	0.189677	0.0326623	0.163953	0.515002	4.61827	0.995103	0.88605	5.38095	1.47297	0.314138	0.48405	0.340227	2.82893	51.6589	42.6677	3.58297	3.6225	2.07	2.07	0
0.0152772	0.0982946	11.32	0.0318484	0.0255398	21.3486	0.189677	0.0326623	0.163953	0.515002	4.61827	0.995103	0.88605	5.38095	1.47297	0.314138	0.48405	0.340227	2.82893	51.6589	42.6677	3.58297	3.51382	2.0079	2.0079	0
0.015015	0.100931	7.0663	0.233986	0.143221	8.5083	0.200344	0.0812296	0.202993	0.910987	18.0351	1.11141	1.03386	15.1248	1.53075	0.332264	0.493861	0.435258	2.7994	60.4551	48.7345	5.5809	3.6	1	1	0
0.015015	0.100931	7.0663	0.233986	0.143221	8.5083	0.200344	0.0812296	0.202993	0.910987	18.0351	1.11141	1.03386	15.1248	1.53075	0.332264	0.493861	0.435258	2.7994	60.4551	48.7345	5.5809	3.726	1.035	1.035	0
0.015015	0.100931	7.0663	0.233986	0.143221	8.5083	0.200344	0.0812296	0.202993	0.910987	18.0351	1.11141	1.03386	15.1248	1.53075	0.332264	0.493861	0.435258	2.7994	60.4551	48.7345	5.5809	3.726	1.035	1.035	0
0.015015	0.100931	7.0663	0.233986	0.143221	8.5083	0.200344	0.0812296	0.202993	0.910987	18.0351	1.11141	1.03386	15.1248	1.53075	0.332264	0.493861	0.435258	2.7994	60.4551	48.7345	5.5809	3.85641	1.07123	1.07123	0
0.0180167	0.100199	7.71123	0.0466989	0.0468645	16.3327	0.195009	0.0639586	0.257831	0.33659	3.74514	0.796864	0.666765	4.38214	1.55699	0.310759	0.481215	0.337494	2.72014	41.1363	32.1712	3.48167	4.9	1.4	2.2	0
0.0180167	0.100199	7.71123	0.0466989	0.0468645	16.3327	0.195009	0.0639586	0.257831	0.33659	3.74514	0.796864	0.666765	4.38214	1.55699	0.310759	0.481215	0.337494	2.72014	41.1363	32.1712	3.48167	4	4	4	0
0.0180167	0.100199	7.71123	0.0466989	0.0468645	16.3327	0.195009	0.0639586	0.257831	0.33659	3.74514	0.796864	0.666765	4.38214	1.55699	0.310759	0.481215	0.337494	2.72014	41.1363	32.1712	3.48167	3	1.8	2.5	0
0.0180167	0.100199	7.71123	0.0466989	0.0468645	16.3327	0.195009	0.0639586	0.257831	0.33659	3.74514	0.796864	0.666765	4.38214	1.55699	0.310759	0.481215	0.337494	2.72014	41.1363	32.1712	3.48167	4.1	0	1	0
0.0180167	0.100199	7.71123	0.0466989	0.0468645	16.3327	0.195009	0.0639586	0.257831	0.33659	3.74514	0.796864	0.666765	4.38214	1.55699	0.310759	0.481215	0.337494	2.72014	41.1363	32.1712	3.48167	4.2	2.6	4.4	0
0.0196876	0.109424	8.71015	0.0256298	0.022091	21.2943	0.190874	0.0596497	0.186127	0.213788	4.16466	0.751226	0.461997	4.67105	1.41845	0.303059	0.481625	0.336954	2.70235	46.9289	38.8401	3.06762	3.6	1.6	0	0
0.0196876	0.109424	8.71015	0.0256298	0.022091	21.2943	0.190874	0.0596497	0.186127	0.213788	4.16466	0.751226	0.461997	4.67105	1.41845	0.303059	0.481625	0.336954	2.70235	46.9289	38.8401	3.06762	3.6	0.3	0.3	0
0.0196876	0.109424	8.71015	0.0256298	0.022091	21.2943	0.190874	0.0596497	0.186127	0.213788	4.16466	0.751226	0.461997	4.67105	1.41845	0.303059	0.481625	0.336954	2.70235	46.9289	38.8401	3.06762	4.01616	0.33468	0.33468	0
0.0196876	0.109424	8.71015	0.0256298	0.022091	21.2943	0.190874	0.0596497	0.186127	0.213788	4.16466	0.751226	0.461997	4.67105	1.41845	0.303059	0.481625	0.336954	2.70235	46.9289	38.8401	3.06762	3.6772	0.306433	0.306433	0
0.01403	0.09234	6.77342	0.0447241	0.0398662	18.2516	0.19278	0.00859819	0.226818	1.52636	3.76498	0.776226	0.534237	4.41932	1.46648	0.309107	0.484664	0.336466	2.77064	42.9617	33.2093	4.36008	2.8	0.7	0.5	0
0.01403	0.09234	6.77342	0.0447241	0.0398662	18.2516	0.19278	0.00859819	0.226818	1.52636	3.76498	0.776226	0.534237	4.41932	1.46648	0.309107	0.484664	0.336466	2.77064	42.9617	33.2093	4.36008	2.4	0	0	0
0.01403	0.09234	6.77342	0.0447241	0.0398662	18.2516	0.19278	0.00859819	0.226818	1.52636	3.76498	0.776226	0.534237	4.41932	1.46648	0.309107	0.484664	0.336466	2.77064	42.9617	33.2093	4.36008	3.12368	0.78092	0.5578	0
0.01403	0.09234	6.77342	0.0447241	0.0398662	18.2516	0.19278	0.00859819	0.226818	1.52636	3.76498	0.776226	0.534237	4.41932	1.46648	0.309107	0.484664	0.336466	2.77064	42.9617	33.2093	4.36008	2.86004	0.71501	0.510722	0
0.0150613	0.0829242	6.91956	0.110997	0.079141	13.4281	0.199802	0.0265784	0.200946	0.291944	4.4493	2.90908	0.570512	5.77136	1.54343	0.319096	0.487927	0.353891	2.76376	41.4268	30.5683	5.42878				
0.017358	0.108926	8.99185	0.0958816	0.068975	19.6924	0.198966	0.0260849	0.185829	1.69862	4.3327	2.96373	0.669726	5.45318	1.50195	0.320008	0.483596	0.347984	2.77465	50.8224	38.4701	6.79329	3.5	0.1	0.7	0
0.0153804	0.0737932	5.29924	0.0544889	0.0397791	9.97058	0.191815	0.0417827	0.328709	0.153469	4.44416	1.13767	0.497808	5.83169	1.37306	0.302605	0.484768	0.34064	2.81747	34.4038	25.5457	3.54035	2.9	0.2	0.6	0
0.015661	0.0890146	6.78969	0.0343792	0.0284912	14.0453	0.197423	0.0651274	0.173421	0.899163	3.99106	0.975956	0.706295	4.9462	1.47008	0.315891	0.484409	0.350402	2.85834	39.4475	29.7723	4.09743				
0.0168391	0.112979	24.6294	0.0742874	0.0721371	40.3101	0.198442	0.0360396	0.201831	0.732776	5.7992	0.866646	0.519021	6.34995	1.59338	0.311297	0.481953	0.349129	2.72642	86.6331	77.0886	3.84349	3.2	1.2	0.5	0
0.0150957	0.091859	19.2856	0.040833	0.0382743	33.7811	0.201773	0.0334586	0.224235	1.19909	4.60967	0.933501	0.484613	4.85177	1.53375	0.306941	0.483234	0.345231	2.73763	72.148	62.5281	4.26906				
0.020277	0.0764504	7.15784	0.0736372	0.0731218	13.8679	0.196611	0.024223	0.161274	1.77962	4.33024	0.815895	0.65091	5.35913	1.43511	0.306708	0.484844	0.349696	2.74904	41.5112	30.7151	4.71826	3.5	1.5	1.5	0
0.020277	0.0764504	7.15784	0.0736372	0.0731218	13.8679	0.196611	0.024223	0.161274	1.77962	4.33024	0.815895	0.65091	5.35913	1.43511	0.306708	0.484844	0.349696	2.74904	41.5112	30.7151	4.71826	2.2	0.7	1.7	0
0.020277	0.0764504	7.15784	0.0736372	0.0731218	13.8679	0.196611	0.024223	0.161274	1.77962	4.33024	0.815895	0.65091	5.35913	1.43511	0.306708	0.484844	0.349696	2.74904	41.5112	30.7151	4.71826	3.4	1.3	2.4	0
0.020277	0.0764504	7.15784	0.0736372	0.0731218	13.8679	0.196611	0.024223	0.161274	1.77962	4.33024	0.815895	0.65091	5.35913	1.43511	0.306708	0.484844	0.349696	2.74904	41.5112	30.7151	4.71826				
0.0147949	0.100683	12.3811	0.0874018	0.0694647	25.2844	0.195229	0.135958	0.304562	0.90676	5.30534	1.34896	0.737237	6.54	1.54044	0.302507	0.476747	0.357242	2.74017	59.8083	49.5109	4.55583	2.8	0	0	0
0.0147949	0.100683	12.3811	0.0874018	0.0694647	25.2844	0.195229	0.135958	0.304562	0.90676	5.30534	1.34896	0.737237	6.54	1.54044	0.302507	0.476747	0.357242	2.74017	59.8083	49.5109	4.55583	1.9	0	0	0
0.0147949	0.100683	12.3811	0.0874018	0.0694647	25.2844	0.195229	0.135958	0.304562	0.90676	5.30534	1.34896	0.737237	6.54	1.54044	0.302507	0.476747	0.357242	2.74017	59.8083	49.5109	4.55583	3.12368	0	0	0
0.0147949	0.100683	12.3811	0.0874018	0.0694647	25.2844	0.195229	0.135958	0.304562	0.90676	5.30534	1.34896	0.737237	6.54	1.54044	0.302507	0.476747	0.357242	2.74017	59.8083	49.5109	4.55583	2.86004	0	0	0
0.0182916	0.0934551	11.2199	0.054074	0.0371239	17.8013	0.192935	0.0874484	0.189271	0.401206	4.61032	3.65821	0.45114	5.78045	1.41501	0.304817	0.481042	0.335715	2.71663	51.0525	39.412	6.35597				
0.0182916	0.0934551	11.2199	0.054074	0.0371239	17.8013	0.192935	0.0874484	0.189271	0.401206	4.61032	3.65821	0.45114	5.78045	1.41501	0.304817	0.481042	0.335715	2.71663	51.0525	39.412	6.35597				
0.0182916	0.0934551	11.2199	0.054074	0.0371239	17.8013	0.192935	0.0874484	0.189271	0.401206	4.61032	3.65821	0.45114	5.78045	1.41501	0.304817	0.481042	0.335715	2.71663	51.0525	39.412	6.35597				
0.0182916	0.0934551	11.2199	0.054074	0.0371239	17.8013	0.192935	0.0874484	0.189271	0.401206	4.61032	3.65821	0.45114	5.78045	1.41501	0.304817	0.481042	0.335715	2.71663	51.0525	39.412	6.35597				
0.0164092	0.120804	15.4918	0.0230388	0.0219112	38.7249	0.188764	0.0278738	0.145075	0.622488	4.46018	0.77546	0.506231	4.76744	1.48373	0.308911	0.47782	0.342393	2.83615	72.0916	63.4443	3.38409	2.5	0	1	0
0.0164092	0.120804	15.4918	0.0230388	0.0219112	38.7249	0.188764	0.0278738	0.145075	0.622488	4.46018	0.77546	0.506231	4.76744	1.48373	0.308911	0.47782	0.342393	2.83615	72.0916	63.4443	3.38409	3	0.6	1.7	0
0.0164092	0.120804	15.4918	0.0230388	0.0219112	38.7249	0.188764	0.0278738	0.145075	0.622488	4.46018	0.77546	0.506231	4.76744	1.48373	0.308911	0.47782	0.342393	2.83615	72.0916	63.4443	3.38409	2.789	0	1.1156	0
0.0164092	0.120804	15.4918	0.0230388	0.0219112	38.7249	0.188764	0.0278738	0.145075	0.622488	4.46018	0.77546	0.506231	4.76744	1.48373	0.308911	0.47782	0.342393	2.83615	72.0916	63.4443	3.38409	2.55361	0	1.02144	0
0.0146287	0.101094	15.4638	0.0535565	0.0366273	30.9761	0.188866	0.0253614	0.271463	0.519716	4.8788	0.807492	0.353923	4.79143	1.48854	0.293039	0.480902	0.348308	2.7432	64.6851	56.1101	3.47709	4.2	1.6	1.8	0
0.0146287	0.101094	15.4638	0.0535565	0.0366273	30.9761	0.188866	0.0253614	0.271463	0.519716	4.8788	0.807492	0.353923	4.79143	1.48854	0.293039	0.480902	0.348308	2.7432	64.6851	56.1101	3.47709	4.074	1.552	1.746	0
0.0146287	0.101094	15.4638	0.0535565	0.0366273	30.9761	0.188866	0.0253614	0.271463	0.519716	4.8788	0.807492	0.353923	4.79143	1.48854	0.293039	0.480902	0.348308	2.7432	64.6851	56.1101	3.47709	4.347	1.656	1.863	0
0.0146287	0.101094	15.4638	0.0535565	0.0366273	30.9761	0.188866	0.0253614	0.271463	0.519716	4.8788	0.807492	0.353923	4.79143	1.48854	0.293039	0.480902	0.348308	2.7432	64.6851	56.1101	3.47709	4.21659	1.60632	1.80711	0
0.0105018	0.0985316	13.0087	0.0343531	0.0296543	18.8411	0.196201	0.0422153	0.268244	1.05912	4.08729	1.06179	0.711243	4.8896	1.70759	0.318425	0.492266	0.34733	2.72457	50.8215	40.8267	4.55385				
0.0105018	0.0985316	13.0087	0.0343531	0.0296543	18.8411	0.196201	0.0422153	0.268244	1.05912	4.08729	1.06179	0.711243	4.8896	1.70759	0.318425	0.492266	0.34733	2.72457	50.8215	40.8267	4.55385				
0.0105018	0.0985316	13.0087	0.0343531	0.0296543	18.8411	0.196201	0.0422153	0.268244	1.05912	4.08729	1.06179	0.711243	4.8896	1.70759	0.318425	0.492266	0.34733	2.72457	50.8215	40.8267	4.55385				
0.0105018	0.0985316	13.0087	0.0343531	0.0296543	18.8411	0.196201	0.0422153	0.268244	1.05912	4.08729	1.06179	0.711243	4.8896	1.70759	0.318425	0.492266	0.34733	2.72457	50.8215	40.8267	4.55385				

0.0153939	0.0795357	17.0712	0.0397262	0.0367145	23.8478	0.197333	0.0448435	0.203838	0.743447	4.47764	0.876445	0.638508	4.70578	1.42423	0.310408	0.492093	0.342742	2.7315	59.0625	50.1024	3.59857	1.7	1	2	0
0.0153939	0.0795357	17.0712	0.0397262	0.0367145	23.8478	0.197333	0.0448435	0.203838	0.743447	4.47764	0.876445	0.638508	4.70578	1.42423	0.310408	0.492093	0.342742	2.7315	59.0625	50.1024	3.59857	1	0	0.6	0
0.0153939	0.0795357	17.0712	0.0397262	0.0367145	23.8478	0.197333	0.0448435	0.203838	0.743447	4.47764	0.876445	0.638508	4.70578	1.42423	0.310408	0.492093	0.342742	2.7315	59.0625	50.1024	3.59857	1.89652	1.1156	2.2312	0
0.0153939	0.0795357	17.0712	0.0397262	0.0367145	23.8478	0.197333	0.0448435	0.203838	0.743447	4.47764	0.876445	0.638508	4.70578	1.42423	0.310408	0.492093	0.342742	2.7315	59.0625	50.1024	3.59857	1.73645	1.02144	2.04289	0
0.0156931	0.0867258	5.53906	0.0328648	0.0321509	9.61787	0.189806	0.0257198	0.231186	0.215254	3.3786	0.917291	1.43627	4.32576	1.44967	0.30437	0.485649	0.336748	2.69921	32.2199	22.8613	3.27792	2.3	1.7	2.5	0
0.0156931	0.0867258	5.53906	0.0328648	0.0321509	9.61787	0.189806	0.0257198	0.231186	0.215254	3.3786	0.917291	1.43627	4.32576	1.44967	0.30437	0.485649	0.336748	2.69921	32.2199	22.8613	3.27792	2.6	1.3	2.9	0
0.0156931	0.0867258	5.53906	0.0328648	0.0321509	9.61787	0.189806	0.0257198	0.231186	0.215254	3.3786	0.917291	1.43627	4.32576	1.44967	0.30437	0.485649	0.336748	2.69921	32.2199	22.8613	3.27792	2.56588	1.89652	2.789	0
0.0156931	0.0867258	5.53906	0.0328648	0.0321509	9.61787	0.189806	0.0257198	0.231186	0.215254	3.3786	0.917291	1.43627	4.32576	1.44967	0.30437	0.485649	0.336748	2.69921	32.2199	22.8613	3.27792	2.34932	1.73645	2.55361	0
0.012652	0.0834678	18.1885	0.0441531	0.0372049	25.3905	0.192876	0.0222824	0.276281	1.76008	4.06067	0.836868	0.623613	4.5643	1.45143	0.297599	0.477105	0.338276	2.70644	62.1332	52.204	4.62445	1.5	0.6	1.1	0
0.012652	0.0834678	18.1885	0.0441531	0.0372049	25.3905	0.192876	0.0222824	0.276281	1.76008	4.06067	0.836868	0.623613	4.5643	1.45143	0.297599	0.477105	0.338276	2.70644	62.1332	52.204	4.62445	2.5	0.7	0	0
0.012652	0.0834678	18.1885	0.0441531	0.0372049	25.3905	0.192876	0.0222824	0.276281	1.76008	4.06067	0.836868	0.623613	4.5643	1.45143	0.297599	0.477105	0.338276	2.70644	62.1332	52.204	4.62445	1.6734	0.66936	1.22716	0
0.012652	0.0834678	18.1885	0.0441531	0.0372049	25.3905	0.192876	0.0222824	0.276281	1.76008	4.06067	0.836868	0.623613	4.5643	1.45143	0.297599	0.477105	0.338276	2.70644	62.1332	52.204	4.62445	1.53217	0.612866	1.12359	0
0.0168975	0.0961584	12.5713	0.0221341	0.0221234	19.4105	0.198812	0.059248	0.265008	0.418783	4.06817	0.940941	1.04795	4.46811	1.526	0.331507	0.486374	0.345767	2.75379	49.963	40.5181	3.6653	2.7	2	2.5	0
0.0168975	0.0961584	12.5713	0.0221341	0.0221234	19.4105	0.198812	0.059248	0.265008	0.418783	4.06817	0.940941	1.04795	4.46811	1.526	0.331507	0.486374	0.345767	2.75379	49.963	40.5181	3.6653	2.3	2	0	0
0.0168975	0.0961584	12.5713	0.0221341	0.0221234	19.4105	0.198812	0.059248	0.265008	0.418783	4.06817	0.940941	1.04795	4.46811	1.526	0.331507	0.486374	0.345767	2.75379	49.963	40.5181	3.6653	3.01212	2.2312	2.789	0
0.0168975	0.0961584	12.5713	0.0221341	0.0221234	19.4105	0.198812	0.059248	0.265008	0.418783	4.06817	0.940941	1.04795	4.46811	1.526	0.331507	0.486374	0.345767	2.75379	49.963	40.5181	3.6653	2.7579	2.04289	2.55361	0
0.00886813	0.0597776	9.2074	0.0238402	0.0223803	16.9792	0.191939	0.0248215	0.272097	0.256637	4.46948	1.03343	0.571201	4.59853	1.45532	0.313695	0.487635	0.348746	2.75949	43.6977	35.2546	3.3286				

																						4	0.8	0.6	0
0.013377	0.080083	16.7891	0.0187447	0.0218758	26.3637	0.198558	0.0268576	0.194537	0.710008	5.20744	0.933845	0.78579	4.99829	1.5028	0.319515	0.485573	0.338575	2.71905	62.423	53.3585	3.71954				
0.0144257	0.110812	21.0367	0.0284586	0.0279503	34.1342	0.201688	0.0655053	0.357025	0.676727	4.78945	0.801044	0.525728	4.61738	1.67878	0.34052	0.491502	0.333298	2.72105	73.7916	64.5777	3.9494	1.8	1	0.9	0
0.0140837	0.11106	21.0882	0.045633	0.0377673	33.0497	0.199164	0.0415385	0.26375	0.479007	5.25875	0.789754	0.47259	4.97487	1.50738	0.334814	0.489917	0.339653	2.71758	73.0874	64.3716	3.46461	2.00808	1.1156	1.00404	0
0.0130146	0.0941208	10.6772	0.0219695	0.020868	17.0178	0.19262	0.0229346	0.153703	0.664362	3.51784	0.827011	0.514683	4.48847	1.46576	0.308994	0.481744	0.338582	2.71412	44.4018	35.7014	3.57289	2	1.1	2.2	0
0.0130146	0.0941208	10.6772	0.0219695	0.020868	17.0178	0.19262	0.0229346	0.153703	0.664362	3.51784	0.827011	0.514683	4.48847	1.46576	0.308994	0.481744	0.338582	2.71412	44.4018	35.7014	3.57289	3.3	2.5	3	0
0.0130146	0.0941208	10.6772	0.0219695	0.020868	17.0178	0.19262	0.0229346	0.153703	0.664362	3.51784	0.827011	0.514683	4.48847	1.46576	0.308994	0.481744	0.338582	2.71412	44.4018	35.7014	3.57289	2.2312	1.22716	2.45432	0
0.0130146	0.0941208	10.6772	0.0219695	0.020868	17.0178	0.19262	0.0229346	0.153703	0.664362	3.51784	0.827011	0.514683	4.48847	1.46576	0.308994	0.481744	0.338582	2.71412	44.4018	35.7014	3.57289	2.04289	1.12359	2.24718	0
0.0157462	0.110156	14.6303	0.0379704	0.0353972	19.3706	0.197233	0.0423664	0.160042	0.514315	4.60794	0.987327	0.703579	5.21144	1.46165	0.328116	0.484349	0.340587	2.71534	52.9873	43.8203	3.6567	2.7	1.4	1.5	0
0.0157462	0.110156	14.6303	0.0379704	0.0353972	19.3706	0.197233	0.0423664	0.160042	0.514315	4.60794	0.987327	0.703579	5.21144	1.46165	0.328116	0.484349	0.340587	2.71534	52.9873	43.8203	3.6567	2.4	0	0	0
0.0157462	0.110156	14.6303	0.0379704	0.0353972	19.3706	0.197233	0.0423664	0.160042	0.514315	4.60794	0.987327	0.703579	5.21144	1.46165	0.328116	0.484349	0.340587	2.71534	52.9873	43.8203	3.6567	3.01212	1.56184	1.6734	0
0.0157462	0.110156	14.6303	0.0379704	0.0353972	19.3706	0.197233	0.0423664	0.160042	0.514315	4.60794	0.987327	0.703579	5.21144	1.46165	0.328116	0.484349	0.340587	2.71534	52.9873	43.8203	3.6567	2.7579	1.43002	1.53217	0
0.0147398	0.083584	16.2827	0.0245616	0.0257967	30.5529	0.197255	0.0744807	0.259838	1.03775	5.3224	1.03907	0.826782	4.93352	1.62013	0.324752	0.487931	0.357716	2.75925	67.0237	57.0916	4.4804				
0.0147398	0.083584	16.2827	0.0245616	0.0257967	30.5529	0.197255	0.0744807	0.259838	1.03775	5.3224	1.03907	0.826782	4.93352	1.62013	0.324752	0.487931	0.357716	2.75925	67.0237	57.0916	4.4804				
0.0147398	0.083584	16.2827	0.0245616	0.0257967	30.5529	0.197255	0.0744807	0.259838	1.03775	5.3224	1.03907	0.826782	4.93352	1.62013	0.324752	0.487931	0.357716	2.75925	67.0237	57.0916	4.4804				
0.0147398	0.083584	16.2827	0.0245616	0.0257967	30.5529	0.197255	0.0744807	0.259838	1.03775	5.3224	1.03907	0.826782	4.93352	1.62013	0.324752	0.487931	0.357716	2.75925	67.0237	57.0916	4.4804				
0.0128429	0.131896	12.8007	0.0220905	0.0185593	20.2047	0.19629	0.063853	0.204154	0.903448	3.75076	1.05294	0.770819	4.39528	1.50532	0.348105	0.489299	0.341382	2.72356	51.0984	41.1515	4.21233	2.8	1.5	2.4	0
0.0128429	0.131896	12.8007	0.0220905	0.0185593	20.2047	0.19629	0.063853	0.204154	0.903448	3.75076	1.05294	0.770819	4.39528	1.50532	0.348105	0.489299	0.341382	2.72356	51.0984	41.1515	4.21233	3.12368	1.6734	2.67744	0
0.0128429	0.131896	12.8007	0.0220905	0.0185593	20.2047	0.19629	0.063853	0.204154	0.903448	3.75076	1.05294	0.770819	4.39528	1.50532	0.348105	0.489299	0.341382	2.72356	51.0984	41.1515	4.21233	2.56368	1.3734	2.19744	0
0.0128429	0.131896	12.8007	0.0220905	0.0185593	20.2047	0.19629	0.063853	0.204154	0.903448	3.75076	1.05294	0.770819	4.39528	1.50532	0.348105	0.489299	0.341382	2.72356	51.0984	41.1515	4.21233				
0.0193743	0.101192	8.32051	0.0227012	0.0213532	13.815	0.193073	0.0463287	0.16409	0.114788	3.67069	0.940454	0.603637	4.34043	1.68632	0.324612	0.489354	0.336465	2.72654	38.8044	30.1466	3.44178	2.1	1	1.5	0
0.0193743	0.101192	8.32051	0.0227012	0.0213532	13.815	0.193073	0.0463287	0.16409	0.114788	3.67069	0.940454	0.603637	4.34043	1.68632	0.324612	0.489354	0.336465	2.72654	38.8044	30.1466	3.44178	2.5	1.5	0	0
0.0193743	0.101192	8.32051	0.0227012	0.0213532	13.815	0.193073	0.0463287	0.16409	0.114788	3.67069	0.940454	0.603637	4.34043	1.68632	0.324612	0.489354	0.336465	2.72654	38.8044	30.1466	3.44178	2.789	1.6734	0	0
0.0193743	0.101192	8.32051	0.0227012	0.0213532	13.815	0.193073	0.0463287	0.16409	0.114788	3.67069	0.940454	0.603637	4.34043	1.68632	0.324612	0.489354	0.336465	2.72654	38.8044	30.1466	3.44178	1.92276	0.9156	1.3734	0
0.0136081	0.107277	19.4859	0.060084	0.047712	22.6166	0.201838	0.0722254	0.20271	2.44405	5.71434	1.20203	0.917762	5.9987	1.61201	0.325514	0.493108	0.351718	2.73294	66.3335	53.8155	6.1485	4.3	1	1	0
0.0136081	0.107277	19.4859	0.060084	0.047712	22.6166	0.201838	0.0722254	0.20271	2.44405	5.71434	1.20203	0.917762	5.9987	1.61201	0.325514	0.493108	0.351718	2.73294	66.3335	53.8155	6.1485	4.79708	1.1156	1.1156	0
0.0136081	0.107277	19.4859	0.060084	0.047712	22.6166	0.201838	0.0722254	0.20271	2.44405	5.71434	1.20203	0.917762	5.9987	1.61201	0.325514	0.493108	0.351718	2.73294	66.3335	53.8155	6.1485	3.93708	0.9156	0.9156	0
0.0136081	0.107277	19.4859	0.060084	0.047712	22.6166	0.201838	0.0722254	0.20271	2.44405	5.71434	1.20203	0.917762	5.9987	1.61201	0.325514	0.493108	0.351718	2.73294	66.3335	53.8155	6.1485				
